# Stable Hemiaminals with a Cyano Group and a Triazole Ring

**DOI:** 10.3390/molecules190811160

**Published:** 2014-07-30

**Authors:** Anna Kwiecień, Maciej Barys, Zbigniew Ciunik

**Affiliations:** Faculty of Chemistry, University of Wrocław, F. Joliot-Curie 14, Wrocław 50-383, Poland; E-Mails: barysiak@o2.pl (M.B.); ciunik@wchuwr.pl (Z.C.)

**Keywords:** stable hemiaminal, stable intermediate, heteroatom hyperconjugation, crystal structure, 4-amino-1,2,4-triazole, formylbenzonitrile

## Abstract

Under neutral conditions the reactions between 4-amino-1,2,4-triazole and cyano-substituted benzaldehyde derivatives yield stable hemiaminals. Addition of small amounts of acid catalyst promotes further step of dehydration resulting in formation of Schiff bases. Four new hemiaminals and the corresponding imines have been obtained. The molecular stability of the hemiaminal intermediates results from both the 1,2,4-triazole moiety and electron withdrawing substituents on the phenyl ring, so no further stabilisation by intramolecular interaction is required. Hemiaminal molecules possess stereogenic centres on carbon and nitrogen atoms. The chirality of these centres is strongly correlated with the conformation of the molecules due to heteroatom hyperconjugation effects.

## 1. Introduction

Hemiaminals (previously incorrectly named carbinolamines [1]) are usually unstable intermediates in the reaction between carbonyl compounds (aldehydes or ketones) and amines [2]. These compounds contain a tetrahedral carbon atom connected with a hydroxyl group and nitrogen atom. Due to instability of these species they were typically obtained and characterised by very sophisticated methods, *i.e.* polarography [3], in FTIR liquid cell [4] or by spectroscopy at low temperature [5].

Rebek *et al.* [6,7] were able to detect presence of hemiaminal inside a synthetic receptor, or a deep cavitand with an introverted aldehyde group. Isolation from the external environment and hydrogen bonding with the cavitand rim enabled NMR measurements of the half-lives of those intermediates. The obtained values spread between 30 min and over 100 h at ambient temperature. What is more, no hemiaminal was observed when the analogous reaction was conducted between amine and receptor with an extroverted aldehyde functionality. Further density functional calculations [8] confirmed that formation of hydrogen bonds within the cavitand plays an important role in stabilisation of a host-guest complex and the hemiaminal intermediate. The theoretical study also matches well with the experimental possibility to trap a hemiaminal inside the cavity, but not outside it in the solution.

Hemiaminal moieties were also detected in the process of post-synthetic modifications of porous metal-organic frameworks (MOFs) [9,10]. Kawamichi *et al.* [9] trapped these intermediates inside the pores of a coordination network. Mounted on a diffractometer, the crystal of MOF with amino groups facing pores was immersed in a gentle flow of an aldehyde solution and hemiaminal was formed *in situ*. Upon cooling the crystal for X-ray measurement the short-lived intermediate was kinetically trapped, which allowed determination of its crystal structure. On the other hand, Morris *et al.* [10] confirmed occurrence of hemiaminal intermediates using solid-state ^15^N-NMR. A zirconium metal-organic framework with amino functionality was post-synthetically modified by treatment with an aldehyde solution. Within the pores the presence of kinetic (hemiaminal) and thermodynamic (aziridine) products as well as protonated amine was detected with an approximate ratio of 5:2:3, respectively.

Dolotko *et al.* [11] were able to observe a short-lived hemiaminal intermediate in a *mechanochemical reaction* (solvent-free) between *p*-toluidine and *o*-vanillin. Yufit and Howard [12] unexpectedly obtained and determined the crystal structure of the intermediate of the reaction between a secondary amine and a cyclic ketone using *in situ* low-temperature co-crystallisation of non-solid components. This phenomenon can be attributed to two pathways concerning kinetics of single steps of the reaction mechanism, *i.e.* at room temperature the dehydration rate is much slower or decreases more upon cooling than the hemiaminal formation rate.

The first stable hemiaminal was obtained in the reaction between 4-cyclohexyl-3-thiosemicarbazide and di-2-pirydyl ketone [13] The compound was further characterised by ^1^H-NMR spectroscopy and X-ray crystallography. Crystals suitable for diffraction experiments were obtained by slow evaporation of a solution. The molecular structure of hemiaminals is stabilised by intramolecular hydrogen bonds.

All the previously mentioned reports refer to open-chain hemiaminals. However some cyclic hemiaminals were also reported. For example the naturally occurring echinocandins possess a reactive hemiaminal group at the ornithine-5-position [14,15]. Some hemiaminals belonging to a neothiobinupharidine family were isolated from *Nuphar luteum* [16]. The hemiaminal functionality very often appears as an intermediate in biologically important enzymatic processes leading to Schiff-base formation [17,18,19]. Other hemiaminals were also obtained as fluoroacyl [20] and *N*-acylpyrrole [5] derivatives.

Another class of stable hemiaminals constitute modified hydrocarbon polycyclic cages such as trishomocubane derivatives [21,22,23,24,25,26]. These compounds are interesting because of their biological activity such as modulation of cocaine-induced effects [21], inhibition of nitric oxide synthase [24] and improving of blood-brain barrier permeability for some non-steroidal anti-inflammatory drugs [23].

Hemiaminals can be also stabilised by coordinating to metal ions. El Mail *et al.* [27] obtained a rhodium complex with an uncoordinated hemiaminal group within a rigid seven-membered metallocyclic ring. Several crystal structures of hemiaminal functionalities acting as *O*- or *N*-donor ligands have been reported [28]

In our previous paper [29] we have presented seven new hemiaminals obtained from 4-amino-1,2,4-triazole and nitro-substituted benzaldehydes. The stability of these compounds emerges from both the electron-withdrawing groups on the phenyl ring and the presence of an electron-rich 1,2,4-triazole ring. This is consistent with Sayer and Jencks’ [30] discovery that hemiaminal formation equilibrium constants increase with the presence of electron-withdrawing groups on the benzaldehyde ring. To determine the influence of *meta*- and *para*- substituents on the reactivity of a functional group the Hammett equation is commonly used [1]. On the basis of Hammett constant similarities [31] between nitro- and cyano- groups several formylbenzonitriles were chosen for further investigation of the influence of substituents on phenyl ring on 4-amino-1,2,4-triazole derivative hemiaminal formation.

**Figure 1:**
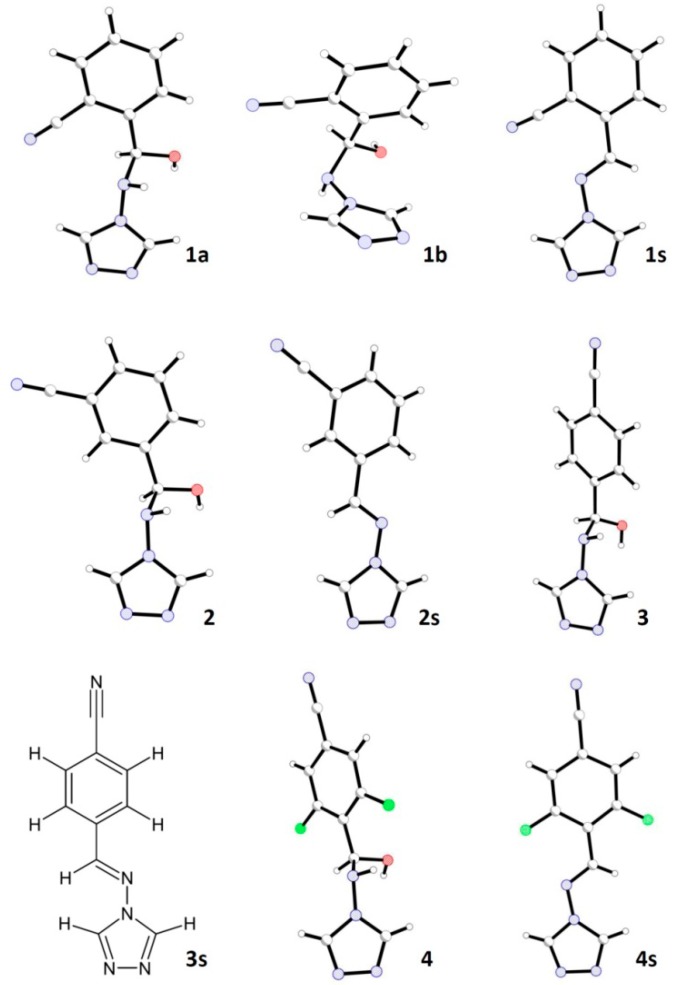
Molecular structures of the new compounds.

The aim of this study was to synthesise and spectroscopically and structurally characterise stable hemiaminals obtained in a very simple way from appropriate substrates in acetonitrile. Five crystals of stable hemiaminals (Figure 1) containing 1,2,4-triazole rings and 2-formylbenzonitrile (**1a** and **1b**, two pseudo-polymorphic crystals), 3-formylbenzonitrile (**2**), 4-formylbenzonitrile (**3**) and 3,5-difluoro-4-formylbenzonitrile (**4**), respectively, were obtained from a 1:1 molar ratio acetonitrile solution. The corresponding Schiff bases (compounds **1s**, **2s**, **3s**, **4s**, Figure 1) were obtained from the same substrates under different conditions, from acidic ethanol solutions.

All the obtained new organic compounds were characterized by elemental analysis, NMR spectroscopy (^1^H, ^13^C, HMQC, HMBC), IR spectroscopy, ESI-MS and for crystalline compounds, X-ray single crystal measurements.

## 2. Results and Discussion

### 2.1. General Remarks

Recently we have shown that reaction between 4-amino-1,2,4-triazole and various benzaldehyde derivatives can lead to both hemiaminals [29] and Schiff bases [32,33,34]. Key factors determining the reaction product are the nature of the substituent on the benzaldehyde ring and the reaction conditions. For electron-donating substituents and reactions catalysed by acid in refluxing alcohol Schiff bases are usually obtained [35]. For electron-withdrawing substituents and reactions carried out in an aprotic solvent at room temperature under neutral conditions hemiaminals were isolated as the sole products.

**Scheme 1:**
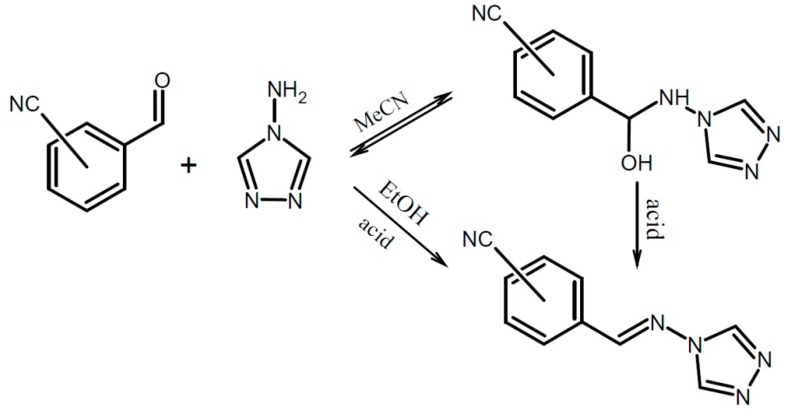
General procedure for hemiaminal and Schiff base synthesis.

Reactions between 4-amino-1,2,4-triazole and benzaldehydes containing electron-withdrawing cyano groups were performed under neutral or acidic conditions to further investigate the role of the substituents and procedures on the nature of the obtained products. To obtain hemiaminals the reaction mixture was typically stirred at room temperature (or slightly higher, up to 50 °C) for 2 h. The products **1a**, **1b**, **2**, **3**, **4** were obtained as single crystals directly from the mother solution upon slow solvent evaporation. The corresponding imines **1s**, **2s**, **3s**, **4s** were synthesised in refluxing ethanol (3–4 h), with addition of hydrochloric acid as a catalyst. Three of them were deposited in crystalline state and thus structurally characterised with X-ray diffraction methods. Compound **3s** was not obtained as suitable single crystals hence it was only characterised spectroscopically. The general procedure for hemiaminal and imine syntheses is depicted in Scheme 1.

### 2.2. Crystal Structures Description

**Figure 2:**
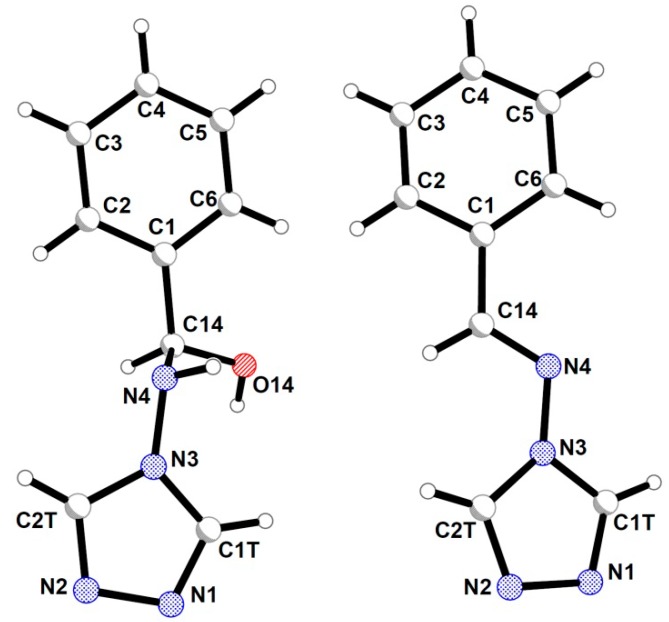
General atom numbering pattern in the crystals of hemiaminals and imines.

The molecular structure of both hemiaminals and Schiff bases consists of two aromatic rings (phenyl and triazole) directly connected with the C1-C14-N4-N3 atom chain linkage (Figure 2). The abovementioned chemical units differ in the hybridization of the C14 and N4 atoms. For hemiaminals these two atoms are tetrahedral with sp^3^ hybridisation which enables formation of stereoisomers; whereas in imines they are double-bonded atoms with sp^2^ hybridisation. The corresponding bond lengths are listed in Table 1 that presents selected geometrical parameters for the analysed compounds.

**Table 1:** Selected geometrical parameters for hemiaminals (**1a**, **1b**, **3**, **4**) and corresponding imines (**1s**, **2s**, **4s**).

	Geometrical	Bond lengths [Å]				Torsion angles [°]		Phenyl/triazole dihedral angle [°]
	parameter							
		C1-C14	C14-N4	N4-N3	C14-O14	N3-N4-C14-C1	N3-N4-C14-O14	
Compound								
**1a**		1.516(1)	1.474(1)	1.408(1)	1.399(1)	167.4(1)	−71.9(1)	8.2(1)
**1b**		1.520(2)	1.468(2)	1.412(2)	1.418(2)	−54.0(1)	68.8(1)	60.4(1)
**2**		1.518(2)	1.470(2)	1.416(2)	1.394(2)	166.1(2)	−72.4(2)	3.9(1)
**3** / OH group disordered in two positions								
position A		1.511(3)	1.469(3)	1.409(2)	1.302(3)	178.0(2)	−59.5(3)	20.4(1)
position B					1.246(3)		39.7(3)	
**4**								
molecule 1		1.525(2)	1.459(3)	1.410(2)	1.394(2)	179.1(2)	−55.8(2)	11.6(2)
molecule 2		1.517(2)	1.468(2)	1.408(2)	1.410(2)	168.0(2)	−71.4(2)	54.3(8)
**1s**		1.465(2)	1.272(2)	1.393(2)	–	179.6(1)	–	27.9(1)
**2s**								
molecule 1		1.464(2)	1.284(2)	1.391(2)	–	178.0(2)	–	11.4(1)
molecule 2		1.469(2)	1.278(2)	1.386(2)	–	177.9(2)	–	6.5(1)
**4s**								
molecule 1		1.463(3)	1.276(3)	1.390(3)	–	179.7(2)	–	18.4(1)
molecule 2		1.465(3)	1.278(3)	1.397(3)	–	−177.9(2)	–	16.3(1)

**Figure 3:**
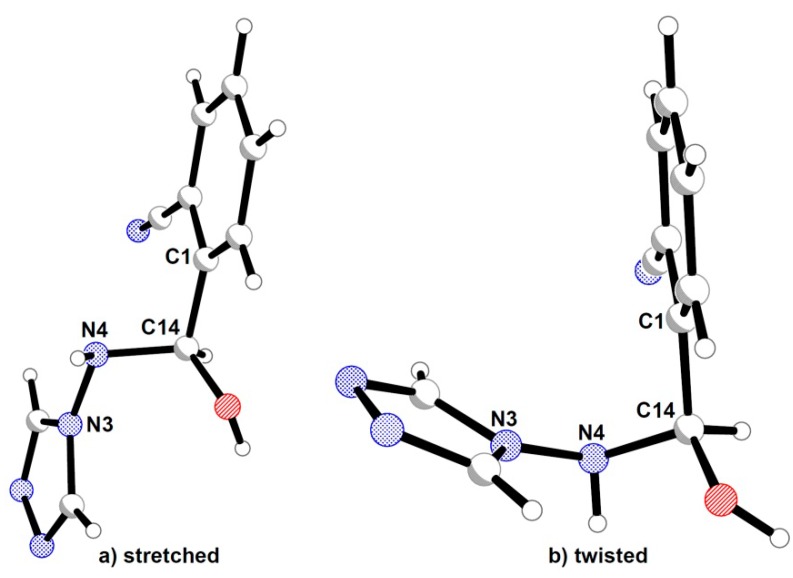
(**a**) Stretched and (**b**) twisted conformer of a hemiaminal.

Within a crystal structure hemiaminals containing a triazole ring and a cyano-substituted phenyl moiety occur in the form of two conformers, determined by the N3-N4-C14-C1 torsion angle. In the first isomer, the stretched one (Figure 3a) the values of the above torsion angle are close to 180° (Table 1) and thus result in an antiperiplanar (*ap*) conformation. Furthermore in the compounds **1a**, **2**, **3** and molecule 1 of **4** the planes defined by triazole and phenyl ring are almost parallel to each other (Table 1). For the twisted molecule (Figure 3b) the N3-N4-C14-C1 chain of atoms is in a synclinal (*sc*) conformation and the aromatic planes are nearly perpendicular. Among the studied compounds only one (compound **1b**) was obtained as a twisted isomer. Moreover from the reaction of 4-amino-1,2,4-triazole and 2-formylbenzonitrile crystals of both stretched and twisted conformers were isolated and characterised. Values of analogues N3-N4-C14-C1 torsion angle in imines oscillate around 180°, which indicates the *E-* geometry of the double N4-C14 bond.

As was noted before, the carbon atom C14 and nitrogen atom N4 have sp^3^ hybridisation and thus can form stereoisomers. From all possible combinations only *RS* (and mirror images *SR*) are present within crystal structures of hemiaminals, namely in stretched conformers. The twisted conformer possess *RR* (and *SS*) configuration.

**Figure 4:**
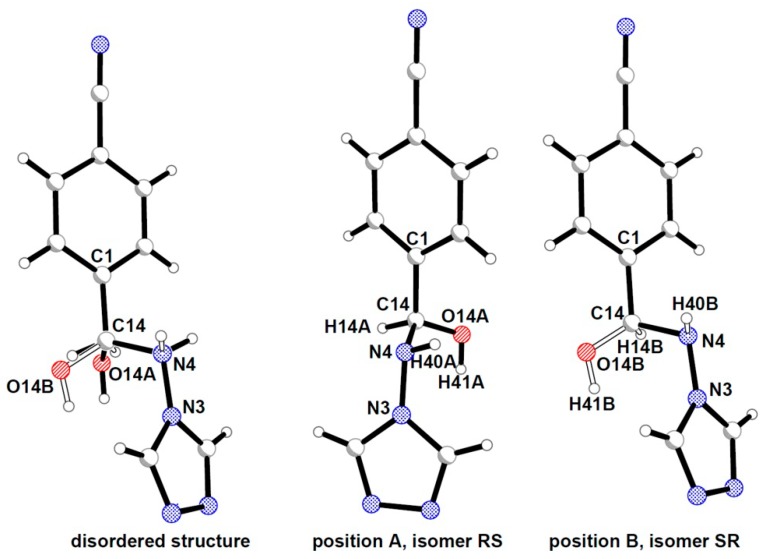
Model of the disorder in the crystal structure of **3**.

In the crystal structure of compound **3** the hydroxyl group is split into two positions, with occupations of 60% and 40%, respectively, and both form hydrogen bond with the same acceptor atom. This disordered substituent at the asymmetric C14 carbon atom generates the chance of formation of all possible stereoisomers (namely *RS*, *SR*, *RR*, and *SS*). However, for the stretched conformation of analog hemiaminals only the *RS* (and *SR*) configuration was detected. This observation leads to the conclusion that the hydrogen atoms connected directly to C14 and N4 atoms are also split into two positions with the same occupancy as the hydroxyl group (Figure 4).

In the crystal structures of the analysed hemiaminals variety of intermolecular interactions such as O-H∙∙∙N, N-H∙∙∙N/O hydrogen bonds, C-H∙∙∙O/N weaker interactions and stacking are observed. What is noteworthy is that no intramolecular stabilising interaction of these nature is detected.

Apparently one of the above interactions (*i.e.* O-H∙∙∙N_triazole_ type hydrogen bond) is present in all the crystal structures of hemiaminals. By this specific interaction molecules of hemiaminals (except **4**) are bonded together into infinite chains. Furthermore, in all crystal structures these chains are parallel to the shortest lattice vector.

**Figure 5:**
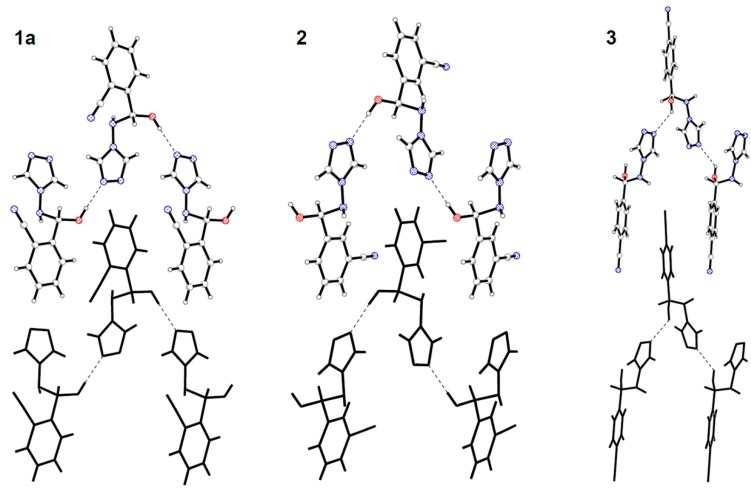
Zipper-like motives in the crystal structures of stretched hemiaminals **1a**, **2** and **3**.

In the crystal structures of hemiaminals in stretched conformation (except **4**) the O-H∙∙∙N type hydrogen bonds links molecules with the opposite configuration into infinite -*RS*-*SR*-*RS*- chains. Chains in turn form a two-dimensional zipper-like motif (Figure 5). In the crystal structure of **3**, both disordered hydroxyl groups form hydrogen bonds with the same nitrogen atom from the triazole ring as an acceptor (for clarity in Figure 5 only one disordered group is presented).

**Figure 6:**
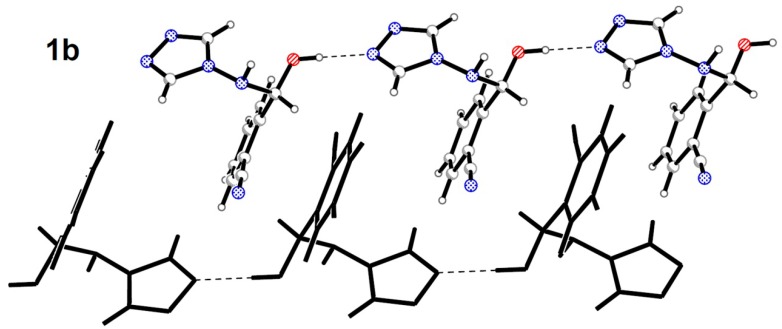
Single column zipper-like motif in the crystal structure of twisted hemiaminal **1b.**

Hemiaminal **1b** also forms in the crystal structure infinite chains of molecules bonded with the O-H∙∙∙N type interactions. However in the case of the twisted conformer molecules of the same configuration are interconnected into -*RR*-*RR*- (and mirror image -*SS*-*SS*-) chains. Two chains with the opposite chirality are parallel to each other and form a single column zipper-like motif along the shortest lattice vector in the crystal (Figure 6). This pattern is additionally stabilised by stacking in pairs between phenyl rings.

**Figure 7:**
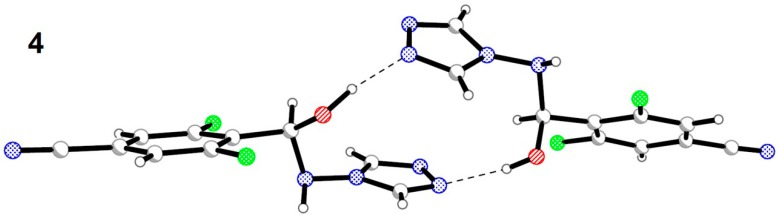
Formation of a dimer in the crystal structure of hemiaminal **4**.

In the crystal structure of hemiaminal **4** the O-H∙∙∙N hydrogen bond type joins two molecules with the same chirality into a dimer (Figure 7).

**Figure 8:**
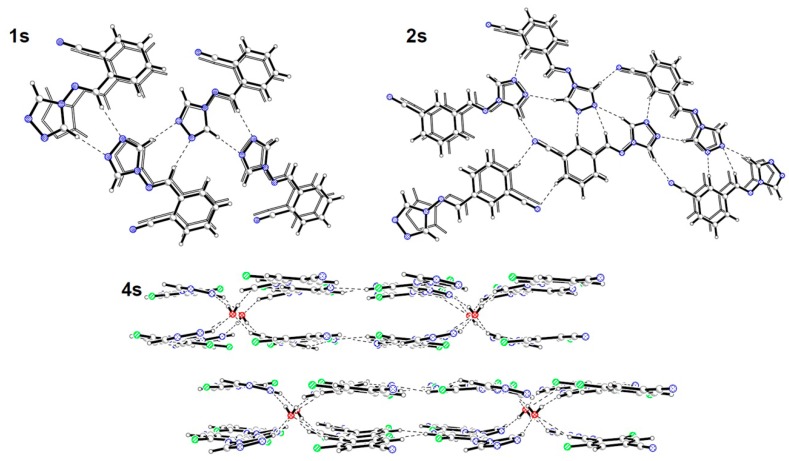
Structural motives in the crystals of imines **1s**, **2s** and **4s**.

In the crystal structures of Schiff bases **1s** and **2s** two types of intermolecular interaction are determined. Along the shortest lattice vector molecules are stacked into piles. Additionally CH∙∙∙N hydrogen bonds form one dimensional infinite chains and two dimensional sheets in compounds **1s** and **2s**, respectively (Figure 8). In the crystal structure of compound **4s** the hydrogen bond network is more complex, due to cocrystallisation with a water molecule, in the stoichiometry hemiaminal to water equal to 2:1. Intermolecular C-H∙∙∙N type interaction joins molecules into two dimensional sheets. Two sheets are interconnected by water molecules into a double layer motif. Between aromatic rings numerous stacking interactions are present. Compound **3s** has not been obtained in the crystalline state.

### 2.3. Discussion

Recently obtained hemiaminals were usually highly unstable compounds requiring additional stabilisation by low temperature [5,12], isolation from the external environment (by molecular cavitands [6,7] or metal-organic frameworks [9,10]) or intramolecular hydrogen bonds [13]. In the studied hemiaminals containing a 1,2,4-triazole and cyano-substituted phenyl ring the stability is the effect of their electronic nature. The cyano group, a strongly electron-withdrawing substituent, affects the possibility of obtaining hemiaminals in a similar manner as the nitro group [29], enhancing the stability of the intermediates [30]. Further stabilisation arises from the presence of the electron-rich 1,2,4-triazole ring.

In the molecular structures of hemiaminals no intramolecular hydrogen bonds were observed. Such finding validates the conclusion that stability of studied intermediates emerges from electronic effects obtained by the proper choice of substituents. Intermolecular O-H∙∙∙N type hydrogen bonds joining molecules of **1a**, **1b**, **2** and **3** into infinite chains are not the key factor responsible for the molecular stability. Rather, the parallel to the shortest lattice vector orientation of these chains implies that such an interaction is a factor controlling crystal growth.

**Scheme 2:**
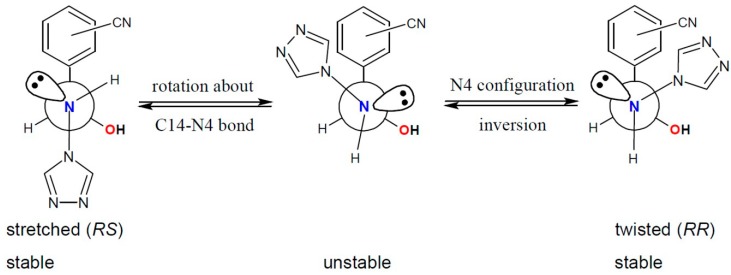
Conformation—configuration conjunction in the hemiaminals.

As was previously discovered in the hemiaminals containing nitro group(s) [29], the conformation of the molecules in the crystal structure is strongly correlated with the configuration on the carbon C14 and nitrogen N4 atoms. The stretched ones exhibit the opposite configuration on these atoms (*i.e. RS* or *SR*) whereas the twisted ones have the same descriptor of chirality (*RR* or *SS*). Such a phenomenon can be attributed to the heteroatom hyperconjugation [36], the anomeric effect [37] in the acyclic molecules, so when in rotation about the C14-N4 single bond the lone election pair comes close to the oxygen atom, the obtained conformer is less stable and thus the inversion of the configuration of N4 nitrogen atom follows (Scheme 2). This conjunction between conformation and configuration is possible due to low energetic barrier of both processes—rotation about single C14-N4 bond and inversion of the configuration on N4 nitrogen atom.

## 3. Experimental Section 

### 3.1. Materials and Methods

All the syntheses were performed form commercially available compounds (Sigma-Aldrich Chemie GmbH, Steinheim, Germany) and solvents (POCh, Gliwice, Poland) without further purification. NMR spectra were measured on Bruker Avance III 500 MHz and Bruker Avance III 600 MHz spectrometers. IR spectra were recorded in KBr pellets on Bruker 66/s FTIR and Bruker Vertex 70 FTIR spectrometers. Mass spectra were measured on Bruker Apex Ultra ESI-MS spectrometer. Elemental analyses were carried out a CHNS Vario EL III analyser (Elementar Analysensystem GmbH, Hanau, Germany).

### 3.2. Crystallography

Single crystal X-Ray diffraction data were collected at a Kuma KM4CCD four-circle diffractometer with Mo Kα radiation and CCD camera (Sapphire), compounds **1b**, **1s**, **2**, **2s**, **3** and **4**; Xcalibur PX four-circle diffractometer with Mo Kα radiation and CCD camera (Onyx), compound **1a**; and Xcalibur four-circle diffractometer with Mo Kα radiation CCD camera (Ruby), compound **4s**. Measurements for all the compounds were carried out at 100 K using an Oxford Cryosystem adapter [38]. Programmes used for data collection and data reduction: CrysAlis CCD, Oxford Diffraction Ltd.; CrysAlis RED, Oxford Diffraction Ltd.; and CrysAlisPro, Agilent Technologies [39]. Structures were solves by direct methods with *SHELXS* [40] program and then refined by a full-matrix least squares method with *SHELXL97* [40] program with anisotropic thermal parameters for nonhydrogen atoms. Molecular graphics were prepared with the XP program [41]. Data for publication were prepared with the programs *SHELXL97* [40] and PLATON [42]. CCDC 1000415–1000422 contain the supplementary crystallographic data for this paper. These data can be obtained free of charge via http://www.ccdc.cam.ac.uk/conts/retrieving.html (or from the CCDC, 12 Union Road, Cambridge CB2 1EZ, UK; Fax: +44 1223 336033; E-mail: deposit@ccdc.cam.ac.uk).

### 3.3. Synthetic Procedures

#### 3.3.1. General Procedure for Synthesis of Hemiaminals

Equimolar amounts of 4-amino-1,2,4-triazole and a suitable formylbenzonitrile were dissolved in acetonitrile and stirred on a magnetic stirrer for two h at room temperature (or slightly heated, up to 50 °C). Crystals obtained upon slow evaporation of the solvent were afterward filtered off, washed with small amount of acetonitrile and then diethyl ether and air-dried.

#### 3.3.2. General Procedure for Synthesis of Schiff Bases.

4-Amino-1,2,4-triazole and an appropriate formylbenzonitrile (in molar ratio 1:1) were dissolved in ethanol with the addition of few drops of an acidic catalyst, namely hydrochloric acid. The reaction mixture was then refluxed for 4 h. Crystals of three imines were obtained upon slow evaporation of the solvent, and one compound was not obtained in a crystalline state. The products were filtered off, washed with small amount of ethanol, diethyl ether and then dried in the air.

#### 3.3.3. *2-[Hydroxy(4H-1,2,4-triazol-4-ylamino)methyl]benzonitriles* (**1a** and **1b**)

Upon standing of the reaction mixture for few days at room temperature, colourless crystal blocks of *2-[hydroxy(4H-1,2,4-triazol-4-ylamino)methyl]benzonitrile* were deposited as two pseudo polymorphic crystals (**1a**, stretched conformation, cocrystal with an acetonitrile molecule; **1b**, twisted conformation) (52 mg, 62%), mp 92 °C. Calculated: C 55.81, H 4.22, N 32.54%; found: C 55.75, H 4.17, N 32.58%. MS: (*m/z*) 216.1 [M + H]^+^. ^1^H-NMR (600 MHz, DMSO, RT) 8.35 (s, 2H, H1T, H2T), 7.86–7.88 (m, 1H, H3), 7.69–7.72 (m, 1H, H5), 7.63–7.65 (m, 1H, H6), 7.53–7.55 (m, 2H, H40, H4), 7.01 (d, ^3^*J*_H41,H14_ = 5.4 Hz, 1H, H41), 5.69 (dd, ^3^*J*_H14,H40_ = 7.2 Hz, ^3^*J*_H14,H41_ = 5.4 Hz, 1H, H14). ^13^C-NMR (150.9 MHz, DMSO, RT): 142.6 (C1T, C2T), 141.6 (C1), 131.9 (C3, C5), 128.0 (C4), 125.7 (C6), 116.2 (C7), 109.4 (C2), 80.8 (C14). IR (KBr, cm^−1^): 3191vs, 3110vs, 3082vs, 2994s, 2854m, 2228s, 1942vw, 1700vw, 1669vw, 1653w, 1636w, 1601w, 1580vw, 1554s, 1504m, 1487w, 1450w, 1356w, 1335w, 1313m, 1287vw, 1268w, 1211s, 1184m, 1161vw, 1111m, 1090vw, 1071vs, 1052vs, 1038m, 976m, 953w, 898m, 880m, 837m, 784w, 758vs, 696m, 639vs, 618m, 575w, 560s, 496w, 473vw, 414vw, 382vw.

Crystal data (**1a**): 2(C_10_H_9_N_5_O) × CH_3_CN, M = 471.50, crystal system: crystal system: monoclinic, space group: *C*2/c, *a* = 24.683(6) Å, *b* = 10.509(3) Å, *c* = 8.826(3) Å, *β* = 95.23(3)°, *V* = 2279.9(2) Å^3^, Z = 4, crystal size: 0.52 × 0.42 × 0.15 mm, *ρ*_c_ = 1.374 g cm^−3^, *μ* = 0.096 mm, *θ*_max_ = 38.51°, reflections: 21879, independent: 6177, *R*_int_ = 0.0233, *R*_1_ = 0.0420, *wR*_2_ = 0.1139, GoF = 1.010.

Crystal data (**1b**): C_10_H_9_N_5_O, *M* = 215.22, crystal system: monoclinic, space group: *P*2_1_/n, *a* = 7.574(3) Å, *b* = 17.464(5) Å, *c* = 7.908(3) Å, *β* = 91.01(3)°, *V* = 1045.8(6) Å^3^, Z = 4, crystal size: 0.41 × 0.27 × 0.21 mm, *ρ*_c_ = 1.367 g cm^−3^, *μ* = 0.096 mm, *θ*_max_ = 36.93°, reflections: 10163, independent: 3550, *R*_int_ = 0.0257, *R*_1_ = 0.0421, *wR*_2_ = 0.1069, GoF = 1.079.

#### 3.3.4. *2-[(E)-(4H-1,2,4-Triazol-4-ylimino)methyl]benzonitrile* (**1s**)

Upon standing of the reaction mixture for few days at room temperature, pale yellow crystal needles of 2-[(*E*)-(4*H*-1,2,4-triazol-4-ylimino)methyl]benzonitrile were deposited (37 mg, 48%), mp 197 °C. Calculated: C 60.91, H 3.58, N 35.51%; found: C 60.78, H 3.47, N 35.25%. MS: (*m/z*) 198.1 [M + H]^+^. ^1^H-NMR (500 MHz, DMSO, RT): 9.22 (s, 2H, H1T, H2T), 9.16 (s, 1H, H14), 8.08–8.10 (m, 1H, H6), 8.04–8.06 (m, 1H, H3), 7.88–7.92 (m, 1H, H4), 7.77–7.80 (m, 1H, H5). ^13^C-NMR (125.8 MHz, DMSO, RT): 154.4 (C14), 139.1 (C1T, C2T), 134.7 (C3), 134.0 (C2), 133.7 (C4), 132.4 (C5), 129.2 (C6), 116.8 (C7), 111.0 (C1). IR (KBr, cm^−1^): 3438m, 3141m, 3082s, 3033m, 2959m, 2931m, 2225m, 1717w, 1696w, 1624w, 1592w, 1569w, 1525m, 1507vs, 1491s, 1469m, 1440w, 1397w, 1346vw, 1328vw, 1315w, 1300m, 1277w, 1218s, 1163vs, 1056vs, 999w, 978s, 959w, 940m, 899w, 890w, 873m, 849w, 774vs, 766vs, 734w, 718vw, 671vw, 623vs, 614s, 567w, 560s, 510m, 505m, 459vw.

Crystal data (**1s**): C_10_H_7_N_5_, *M* = 197.21, crystal system: monoclinic, space group: *P*2_1_/n, *a* = 3.969(2) Å, *b* = 28.422(6) Å, *c* = 8.300(3) Å, *β* = 103.31(3)°, *V* = 911.1(6) Å^3^, Z = 4, crystal size: 0.47 × 0.29 × 0.20 mm, *ρ*_c_ = 1.438 g cm^−3^, *μ* = 0.096 mm, *θ*_max_ = 28.84°, reflections: 6590, independent: 2223, *R*_int_ = 0.0168, *R*_1_ = 0.0363, *wR*_2_ = 0.1135, GoF = 0.947.

#### 3.3.5. *3-[Hydroxy(4H-1,2,4-triazol-4-ylamino)methyl]benzonitrile* (**2**)

Upon standing of the reaction mixture for few days at room temperature, colourless crystal blocks of 3-[hydroxy(4*H*-1,2,4-triazol-4-ylamino)methyl]benzonitrile were obtained (56 mg, 78%), mp 115 °C. Calculated: C 55.81, H 4.22, N 32.54%; found: C 55.77, H 4.06, N 32.64%. MS: (*m/z*) 216.1 [M + H]^+^. ^1^H-NMR (600 MHz, DMSO, RT): 8.42 (s, 2H, H1T, H2T), 7.88–7.89 (m, 1H, H2), 7.80–7.82 (m, 2H, H4, H6), 7.58–7.61 (m, 1H, H5), 7.41 (d, ^3^*J*_H40,H14_ = 6.6 Hz, 1H, H40), 6.81 (d, ^3^*J*_H41,H14_ = 5.5 Hz, 1H, H41), 5.57 (pseudo-triplet, ^3^*J*_H14,H40_ = 6.6 Hz, ^3^*J*_H14,H41_ = 5.5 Hz, 1H, H14). ^13^C-NMR (150.9 MHz, DMSO, RT): 143.9 (C1T, C2T), 141.5 (C1), 131.9 (C4), 131.5 (C6), 130.3 (C2), 129.4 (C5), 118.7 (C7), 111.0 (C3), 82.6 (C14). IR (KBr, cm^−1^): 3190s, 3118s, 2995m, 2945m, 2870m, 2737m, 2228s, 1961vw, 1903vw, 1734vw, 1718vw, 1701vw, 1685vw, 1653vw, 1636vw, 1607vw, 1586w, 1557m, 1505m, 1483m, 1427m, 1368w, 1340w, 1316m, 1303m, 1276m, 1232vw, 1198m, 1169w, 1148s, 1107m, 1075vs, 1061vs, 1001vw, 987m, 954m, 929s, 924s, 888m, 872s, 814m, 779vs, 767s, 708s, 684s, 645vs, 601w, 577m, 554w, 480m, 463vw, 409vw, 384vw.

Crystal data (**2**): C_10_H_9_N_5_O, *M* = 215.22, crystal system: monoclinic, space group: *P*2_1_/c, *a* = 12.037(4) Å, *b* = 10.265(4) Å, *c* = 8.448(3) Å, *β* = 91.58(3)°, *V* = 1043.4(7) Å^3^, Z = 4, crystal size: 0.27 × 0.26 × 0.18 mm, *ρ*_c_ = 1.370 g cm^−3^, *μ* = 0.096 mm, *θ*_max_ = 29.99°, reflections: 12783, independent: 2946, *R*_int_ = 0.0475, *R*_1_ = 0.0433, *wR*_2_ = 0.0924, GoF = 0.987.

#### 3.3.6. *3-[(E)-(4H-1,2,4-Triazol-4-ylimino)methyl]benzonitrile* (**2s**)

Upon standing of the reaction mixture for few days at room temperature, colourless crystal needles of 3-[(*E*)-(4*H*-1,2,4-triazol-4-ylimino)methyl]benzonitrile were deposited (49 mg, 67%), mp 237 °C. Calculated: C 60.91, H 3.58, N 35.51%; found: C 60.95, H 3.69, N 35.70%. MS: (*m/z*) 198.1 [M + H]^+^. ^1^H-NMR (600 MHz, DMSO, RT): 9.16 (s, 2H, H1T, H2T), 9.15 (s, 1H, H14), 8.22–8.23 (m, 1H, H2), 8.15–8.17 (m, 1H, H6), 8.06–8.08 (m, 1H, H4), 7.77–7.80 (m, 1H, H5). ^13^C-NMR (150.9 MHz, DMSO, RT): 156.2 (C14), 139.0 (C1T, C2T), 135.4 (C4), 133.4 (C1), 132.5 (C6), 131.7 (C2), 130.5 (C5), 118.0 (C7), 112.4 (C3). IR (KBr, cm^−1^): 3443w, 3130m, 3079m, 3062m, 3034w, 2956w, 2231s, 1722vw, 1614w, 1578vw, 1529w, 1505vs, 1487m, 1471w, 1430vw, 1391w, 1345vw, 1329w, 1308w, 1286m, 1250vw, 1226w, 1177vs, 1170vs, 1096vw, 1057vs, 1002vw, 977s, 963w, 942m, 929w, 910vw, 871m, 819vw, 797s, 712m, 685vs, 627m, 606w, 596m, 497m, 475w, 457vw.

Crystal data (**1s**): C_10_H_7_N_5_, *M* = 197.21, crystal system: triclinic, space group: *P*1, *a* = 3.811(2) Å, *b* = 10.734(3) Å, *c* = 23.299(5) Å, *α* = 95.60(3)°, *β* = 90.48(3)°, *γ* = 98.63(3)°, *V* = 937.6(6) Å^3^, Z = 4, crystal size: 0.53 × 0.18 × 0.07 mm, *ρ*_c_ = 1.397 g cm^−3^, *μ* = 0.093 mm, *θ*_max_ = 36.95°, reflections: 13312, independent: 6533, *R*_int_ = 0.0267, *R*_1_ = 0.0513, *wR*_2_ = 0.1510, GoF = 0.975.

#### 3.3.7. *4-[Hydroxy(4H-1,2,4-triazol-4-ylamino)methyl]benzonitrile* (**3**)

Upon standing of the reaction mixture for few days at room temperature, colourless crystal blocks of 4-[hydroxy(4*H*-1,2,4-triazol-4-ylamino)methyl]benzonitrile were obtained (70 mg, 87%), mp 98 °C. Calculated: C 55.81, H 4.22, N 32.54%; found: C 55.89, H 4.09, N 32.48%. MS: (*m/z*) 216.1 [M + H]^+^. ^1^H-NMR (600 MHz, DMSO, RT): 8.40 (s, 2H, H1T, H2T), 7.84–7.85 (m, 2H, H3, H5), 7.67–7.68 (m, 2H, H2, H6), 7.43 (d, ^3^*J*_H40,H14_ = 5.5 Hz, 1H, H40), 6.82 (d, ^3^*J*_H41,H14_ = 4.5 Hz, 1H, H41), 5.59 (pseudo-triplet, ^3^*J*_H14,H40_ = 5.5 Hz, ^3^*J*_H14,H41_ = 4.5 Hz, 1H, H14). ^13^C-NMR (150.9 MHz, DMSO, RT): 145.2 (C1), 143.9 (C1T, C2T), 132.1 (C3, C5), 127.7 (C2, C6), 118.7 (C7), 110.8 (C4), 82.8 (C14). IR (KBr, cm^−1^): 3203s, 3111s, 2852m, 2234s, 1952vw, 1830vw, 1773vw, 1734vw, 1700vw, 1685vw, 1653vw, 1610w, 1554m, 1504s, 1460w, 1406m, 1364w, 1340w, 1316m, 1296m, 1270m, 1205s, 1174w, 1163m, 1111vw, 1093vw, 1067vs, 1055vs, 1019s, 977m, 950s, 936w, 910w, 892m, 864s, 846s, 824s, 773m, 700w, 680w, 641vs, 616s, 590w, 552s, 522m, 483w, 416w, 375vw.

Crystal data (3): C_10_H_9_N_5_O, *M* = 215.22, crystal system: monoclinic, space group: *P*2_1_/c, *a* = 12.485(4) Å, *b* = 7.062(3) Å, *c* = 11.406(4) Å, *β* = 96.25(3)°, *V* = 999.7(6) Å^3^, Z = 4, crystal size: 0.40 × 0.30 × 0.19 mm, *ρ*_c_ = 1.430 g cm^−3^, *μ* = 0.101 mm, *θ*_max_ = 28.68°, reflections: 6999, independent: 2412, *R*_int_ = 0.0438, *R*_1_ = 0.0615, *wR*_2_ = 0.1503, GoF = 1.020.

#### 3.3.8. *4-[(4H-1,2,4-Triazol-4-ylimino)methyl]benzonitrile* (**3s**)

Upon standing of the reaction mixture for few days at r.t. 4-[(4*H*-1,2,4-triazol-4-ylimino)methyl]benzonitrile was deposited in non-crystalline state (41 mg, 51%), mp 235 °C. Calculated: C 60.91, H 3.58, N 35.51%; found: C 60.58, H 3.67, N 35.40%. MS: (*m/z*) 198.1 [M + H]^+^. ^1^H-NMR (500 MHz, DMSO, RT): 9.19 (s, 1H, H14), 9.17 (s, 2H, H1T, H2T), 8.00–8.05 (m, 4H, H2, H3, H5, H6). ^13^C-NMR (125.8 MHz, DMSO, RT): 156.4 (C14), 139.0 (C1T, C2T), 136.4 (C1), 133.1 (C3, C5), 128.8 (C2, C6), 118.3 (C7), 114.0 (C4). IR (KBr, cm^−1^): 3505w, 3092m, 2972m, 2373w, 2229s, 1928w, 1669w, 1616w, 1558vw, 1514s, 1495vs, 1468m, 1414w, 1393s, 1344w, 1328w, 1315m, 1296s, 1281s, 1219s, 1175m, 1163vs, 1108m, 1050vs, 1018m, 981m, 956m, 935s, 881m, 846s, 828vs, 771w, 723w, 701w, 670vw, 647w, 616vs, 552vs, 502w, 483s.

#### 3.3.9. *3,5-Difluoro-4-[hydroxy(4H-1,2,4-triazol-4-ylamino)methyl]benzonitrile* (**4**)

Upon standing of the reaction mixture for few days at room temperature, pale yellow crystal plates of 3,5-difluoro-4-[hydroxy(4*H*-1,2,4-triazol-4-ylamino)methyl]benzonitrile were obtained (51 mg, 90%), mp 131 °C. Calculated: C 47.81, H 2.81, N 27.88%; found: C 47.86, H 2.63, N 28.05%. MS: (*m/z*) 252.1 [M + H]^+^. ^1^H-NMR (600 MHz, DMSO, RT): 8.47 (s, 2H, H1T, H2T), 7.82 (d, ^3^*J*_H,F_ = 7.8 Hz, 2H, H3, H5), 7.62 (d, ^3^*J*_H40,H14_ = 7.8 Hz, 1H, H40), 7.07 (d, ^3^*J*_H41,H14_ = 5.4 Hz, 1H, H41), 5.72 (dd, ^3^*J*_H14,H40_ = 7.8 Hz, ^3^*J*_H14,H41_ = 5.4 Hz, 1H, H14). ^13^C-NMR (150.9 MHz, DMSO, RT): 159.6 (dd, ^1^*J*_C,F_ = 252.0 Hz, ^3^*J*_C,F_ = 9.1 Hz, C2, C6), 143.8 (C1T, C2T), 121.5 (t, ^2^*J*_C,F_ = 17.4 Hz, C1), 116.8 (dd, ^2^*J*_C,F_ = 24.9 Hz, ^4^*J*_C,F_ = 6.8 Hz, C3, C5), 116.5 (t, ^4^*J*_C,F_ = 3.0 Hz, C7), 113.0 (t, ^3^*J*_C,F_ = 12.8 Hz, C4), 77.6 (C14). IR (KBr, cm^−1^): 3284vs, 3129vs, 3087s, 3071vs, 3046m, 2530vw, 2240s, 2190vw, 1763w, 1700vw, 1633vs, 1570vs, 1524vs, 1498m, 1484s, 1455m, 1426vs, 1331s, 1318vs, 1290m, 1260m, 1212vs, 1193s, 1178s, 1128s, 1077s, 1066vs, 1028vs, 974m, 956s, 942m, 904vs, 879vs, 858s, 765w, 738m, 709m, 683m, 649s, 636vs, 628vs, 594m, 550s, 535s, 517s, 484w, 446w, 415w.

Crystal data (**4**): 2(C_10_H_7_F_2_N_5_O) × H_2_O, *M* = 520.43, crystal system: triclinic, space group: *P*1, *a* = 7.456(3) Å, *b* = 7.624(3) Å, *c* = 21.534(5) Å, *α* = 80.83(3)°, *β* = 85.83(3)°, *γ* = 67.98(3)°, *V* = 1120.2(7) Å^3^, Z = 4, crystal size: 0.40 × 0.25 × 0.10 mm, *ρ*_c_ = 1.543 g cm^−3^, *μ* = 0.132 mm, *θ*_max_ = 30.00°, reflections: 17252, independent: 6213, *R*_int_ = 0.0361, *R*_1_ = 0.0507, *wR*_2_ = 0.1212, GoF = 1.041.

#### 3.3.10. *3,5-Difluoro-4-[(E)-(4H-1,2,4-triazol-4-ylimino)methyl]benzonitrile* (**4s**)

Upon standing of the reaction mixture for few days at room temperature, colourless crystal needles of 3,5-difluoro-4-[(*E*)-(4*H*-1,2,4-triazol-4-ylimino)methyl]benzonitrile, 2(C_10_H_5_F_2_N_5_) × H_2_O, were deposited (46 mg, 58%), mp 195 °C. Calculated: C 49.59, H 2.50, N 28.92%; found: C 49.38, H 2.41, N 28.89%. MS: (*m/z*) 234.1 [M + H]^+^. ^1^H-NMR (500 MHz, DMSO, RT): 9.28 (s, 2H, H1T, H2T), 9.10 (s, 1H, H14), 8.00 (d, ^3^*J*_H,F_ = 8.5 Hz, 2H, H3, H5). ^13^C-NMR (125.8 MHz, DMSO, RT): 160.5 (dd, ^1^*J*_C,F_ = 259.8 Hz, ^3^J_C,F_ = 6.9 Hz, C2, C6), 147.5 (C14), 139.1 (C1T, C2T), 117.2 (dd, ^2^*J*_C,F_ = 22.6 Hz, ^4^*J*_C,F_ = 6.3 Hz, C3, C5), 116.3 (t, ^4^*J*_C,F_ = 3.8 Hz, C7), 115.3 (t, ^3^*J*_C,F_ = 13.2 Hz, C4), 115.0 (t,^ 2^*J*_C,F_ = 13.8 Hz, C1). IR (KBr, cm^−1^): 3369m, 3155m, 3111m, 3081m, 3060m, 2263vw, 2239m, 1780vw, 1717vw, 1627s, 1560s, 1501s, 1469w, 1426vs, 1406m, 1328m, 1296w, 1227w, 1211vs, 1183w, 1164vs, 1062vs, 1042vs, 975w, 962w, 940m, 890s, 869m, 727w, 669vw, 646m, 636s, 619m, 565w, 542m, 495vw, 474m, 467w, 424m, 381vw.

Crystal data (**4s**): 2(C_10_H_5_F_2_N_5_) × H_2_O, *M* = 484.40, crystal system: monoclinic, space group: *P*2_1_/a, *a* = 11.548(3) Å, *b* = 12.346(3) Å, *c* = 14.988(4) Å, *β* = 94.98(3) °, *V* = 2128.8(9) Å^3^, Z = 4, crystal size: 0.64 × 0.17 × 0.10 mm, *ρ*_c_ = 1.511 g cm^−3^, *μ* = 0.126 mm, *θ*_max_ = 28.81°, reflections: 8793, independent: 4799, *R*_int_ = 0.0464, *R*_1_ = 0.0674, *wR*_2_ = 0.1634, GoF = 1.086.

## 4. Conclusions 

We have discovered a new class of stable hemiaminals containing a 1,2,4-triazole moiety and a phenyl ring with a cyano group as the substituent. The stability can be attributed to the presence of both electron-withdrawing groups on phenyl ring and 1,2,4-triazole electron-rich ring. Molecular structures of these intermediates do not need extra stabilisation either by intramolecular hydrogen bonding, isolation from the external environment nor low temperatures. Syntheses of hemiaminals from 4-amino-1,2,4-triazole and suitable formylbenzonitriles takes place in a one-pot reaction carried out in a neutral solvent (acetonitrile) and at room (or up to 50 °C) temperature.

In the crystal structures the hemiaminal molecules exhibit two stable stereogenic centres, on the C14 carbon atom and on N4 nitrogen atom. Heteroatom hyperconjugation strongly correlates the conformation of the molecules with the configuration on the nitrogen atom, for the purpose of the antiperiplanar (*ap*) relative position of the hydroxyl group and lone electron pair on the N4 nitrogen atom.

## Supplementary Materials

Supplementary materials (containing synthesis, elemental analysis, mass spectrometry, NMR and IR spectra, view of the structures, crystallographic data) can be accessed at: http://www.mdpi.com/1420-3049/19/8/11160/s1.

## Supplementary Files

Supplementary File 1
