# Supplementary material for: Stable Hemiaminals with a Cyano Group and a Triazole Ring
Source: Molecules. 2014 Jul 30;19(8):11160–77. doi: 10.3390/molecules190811160 (PMC6271600; doi:10.3390/molecules190811160)
Supplement: Supplementary File 1 [file molecules-19-11160-s001.pdf]

Supplementary Materials

Stable Hemiaminals with a Cyano Group and a Triazole Ring

Anna Kwiecień *, Maciej Barys and Zbigniew Ciunik

Faculty of Chemistry, University of Wrocław, F. Joliot-Curie 14, Wrocław 50-383, Poland;
E-Mails: barysiak@o2.pl (M.B.); ciunik@wchuwr.pl (Z.C.)

* Author to whom correspondence should be addressed; E-Mail: aniuta04@gmail.com;
Tel.: +48-71-375-7227.

A table of contents

1 General remarks ... S3
2 X-ray crystallography ... S3
3 Synthetic procedures and characterisation of new compounds ... S3
3.1 2-[hydroxy(4*H*-1,2,4-triazol-4-ylamino)methyl]benzonitrile (1a and 1b) ... S3
3.1.1 Synthesis ... S3
3.1.2 Elemental analysis ... S3
3.1.3 Mass spectrometry ... S4
3.1.4 NMR spectroscopy ... S4
3.1.5 IR spectroscopy ... S6
3.1.6 Crystallography ... S7
3.2 2-[(*E*)-(4*H*-1,2,4-triazol-4-ylimino)methyl]benzonitrile (1s) ... S16
3.2.1 Synthesis ... S16
3.2.2 Elemental analysis ... S16
3.2.3 Mass spectrometry ... S16
3.2.4 NMR spectroscopy ... S16
3.2.5 IR spectroscopy ... S19
3.2.6 Crystallography ... S19
3.3 3-[hydroxy(4*H*-1,2,4-triazol-4-ylamino)methyl]benzonitrile (2) ... S24
3.3.1 Synthesis ... S24
3.3.2 Elemental analysis ... S24
3.3.3 Mass spectrometry ... S24
3.3.4 NMR spectroscopy ... S24
3.3.5 IR spectroscopy ... S27
3.3.6 Crystallography ... S27
3.4 3-[(*E*)-(4*H*-1,2,4-triazol-4-ylimino)methyl]benzonitrile (2s) ... S32
3.4.1 Synthesis ... S32
3.4.2 Elemental analysis ... S32

3.4.3 Mass spectrometry S32
3.4.4 NMR spectroscopy S32
3.4.5 IR spectroscopy S35
3.4.6 Crystallography S35
3.5 4-[hydroxy(4*H*-1,2,4-triazol-4-ylamino)methyl]benzonitrile (3) S42
3.5.1 Synthesis S42
3.5.2 Elemental analysis S42
3.5.3 Mass spectrometry S42
3.5.4 NMR spectroscopy S43
3.5.5 IR spectroscopy S45
3.5.6 Crystallography S45
3.6 4-[(4*H*-1,2,4-triazol-4-ylimino)methyl]benzonitrile (3s) S50
3.6.1 Synthesis S50
3.6.2 Elemental analysis S50
3.6.3 Mass spectrometry S51
3.6.4 NMR spectroscopy S51
3.6.5 IR spectroscopy S53
3.7 3,5-difluoro-4-[hydroxy(4*H*-1,2,4-triazol-4-ylamino)methyl]benzonitrile (4) S54
3.7.1 Synthesis S54
3.7.2 Elemental analysis S54
3.7.3 Mass spectrometry S54
3.7.4 NMR spectroscopy S54
3.7.5 IR spectroscopy S57
3.7.6 Crystallography S57
3.8 3,5-difluoro-4-[(*E*)-(4*H*-1,2,4-triazol-4-ylimino)methyl]benzonitrile (4s) S65
3.8.1 Synthesis S65
3.8.2 Elemental analysis S66
3.8.3 Mass spectrometry S66
3.8.4 NMR spectroscopy S66
3.8.5 IR spectroscopy S69
3.8.6 Crystallography S69
4 References S77

1. General Remarks

All the syntheses were performed form commercially available compounds (Aldrich) and solvents (POCh, Aldrich) without further purification. NMR spectra were measured on Bruker Avance III 500 MHz and Bruker Avance III 600 MHz spectrometers. IR spectra were recorded in KBr pellets on Bruker 66/s FTIR (**1**, **2**, **3**, **4**, **1s**, **2s**, **3s**) and Bruker Vertex 70 FTIR (**4s**) spectrometers. Mass spectra were measured on Bruker Apex Ultra ESI-MS spectrometer. Elemental analysis were carried out on Elemental analyser CHNS Vario EL III, Elementar Analysensystem GmbH.

2. X-ray Crystallography

Single crystal X-Ray diffraction data were collected at Kuma KM4CCD four-circle diffractometer with Mo Kα radiation and CCD camera (Sapphire), compounds **1b**, **1s**, **2**, **2s**, **3** and **4**; Xcalibur PX four-circle diffractometer with Mo Kα radiation and CCD camera (Onyx), compound **1a**; and Xcalibur four-circle diffractometer with Mo Kα radiation CCD camera (Ruby), compound **4s**. Measurements for all the compounds were carried out at 100 K using an Oxford Cryosystem adapter [1]. Programmes used for data collection and data reduction: CrysAlis CCD, Oxford Diffraction Ltd.; CrysAlis RED, Oxford Diffraction Ltd.; and CrysAlisPro, Agilent Technologies [2]. Structures were solves by direct methods with SHELXS [3] program and then refined by a full-matrix least squares method with SHELXL97 [3] program with anisotropic thermal parameters for nonhydrogen atoms. Molecular graphics were prepared with the XP program [4]. Data for publication were prepared with the programs SHELXL97 [3], CIFTAB [3] and PLATON [5]. CCDC 1000415-1000422 contain the supplementary crystallographic data for this paper. These data can be obtained free of charge via http://www.ccdc.cam.ac.uk/conts/retrieving.html (or from the CCDC, 12 Union Road, Cambridge CB2 1EZ, UK; Fax: +44 1223 336033; E-mail: deposit@ccdc.cam.ac.uk).

3. Synthetic Procedures and Characterisation of New Compounds

3.1. 2-[hydroxy(4H-1,2,4-triazol-4-ylamino)methyl]benzonitrile (1a and 1b)

3.1.1. Synthesis

Acetonitrilic solution (3 mL) of 2-formylbenzonitrile (52 mg) was added to an acetonitrilic solution (3 mL) of 4-amino-1,2,4-triazole (33 mg). The reaction mixture after complete dissolution was stirred for 2 hours at room temperature (20 °C). The title compound crystallised directly from the mother liquor. Upon standing 2 days at the room temperature, the solution deposited colourless crystal blocks. The crystals were filtered off, washed with a small amount of acetonitrile and diethyl ether then dried in the air to afford 2-[hydroxy(4*H*-1,2,4-triazol-4-ylamino)methyl]benzonitrile—(52 mg, 62%), mp 92 °C.

3.1.2. Elemental Analysis

	**% C**	**% H**	**% N**
Calculated	55.81	4.22	32.54
Found	55.75	4.17	32.58

3.1.3. Mass Spectrometry

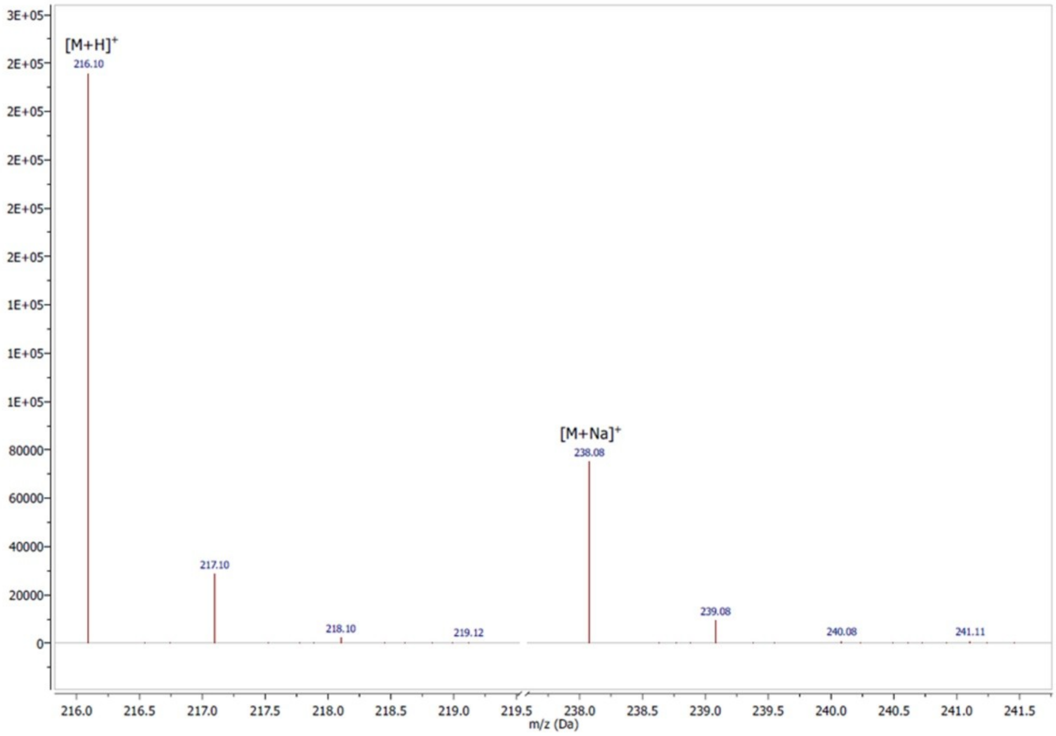

3.1.4. NMR Spectroscopy

^{1}H-NMR

^{1}H-NMR (600 MHz, DMSO, RT) 8.35 (s, 2H, H1T, H2T), 7.86–7.88 (m, 1H, H3), 7.69–7.72 (m, 1H, H5), 7.63–7.65 (m, 1H, H6), 7.53–7.55 (m, 2H, H40, H4), 7.01 (d, $^{3}J_{H41,H14}$ = 5.4 Hz, 1H, H41), 5.69 (dd, $^{3}J_{H14,H40}$ = 7.2 Hz, $^{3}J_{H14,H41}$ = 5.4 Hz, 1H, H14).

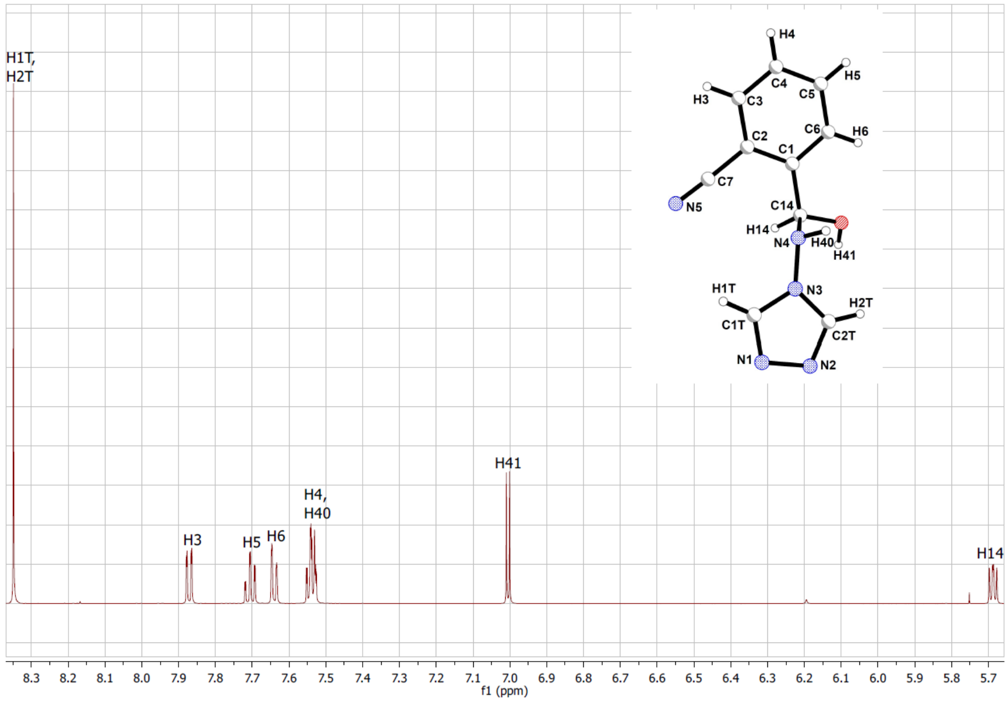

^{13}C-NMR

^{13}C-NMR (150.9 MHz, DMSO, RT): 142.6 (C1T, C2T), 141.6 (C1), 131.9 (C3, C5), 128.0 (C4), 125.7 (C6), 116.2 (C7), 109.4 (C2), 80.8 (C14).

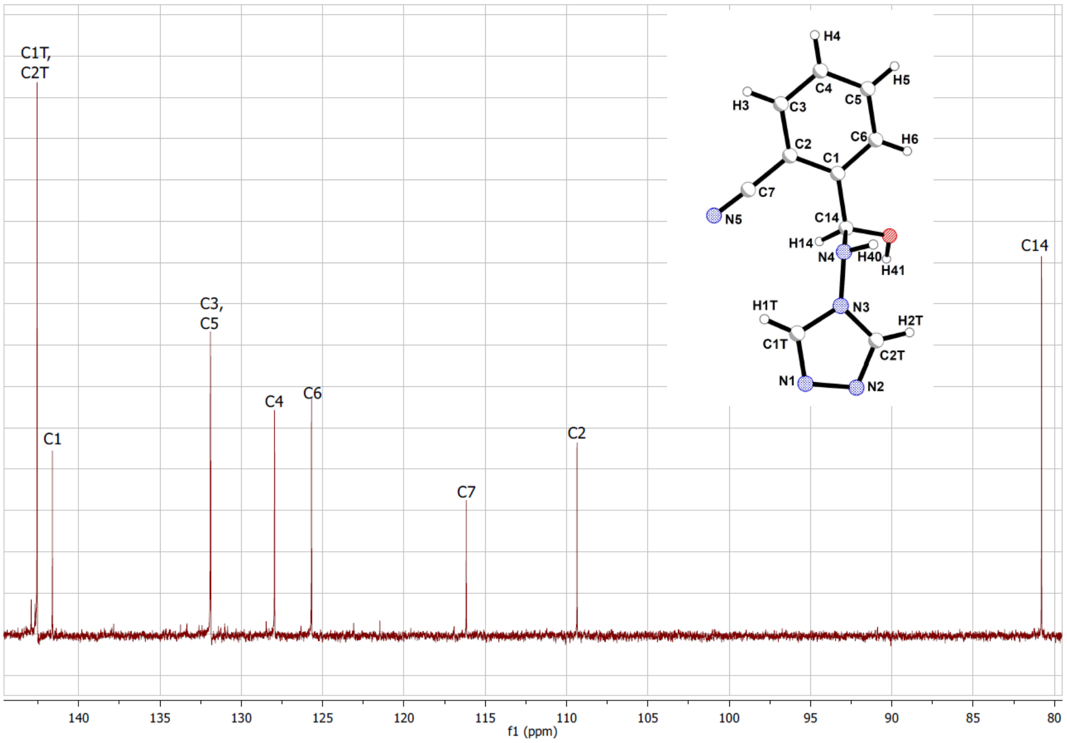

HSQC

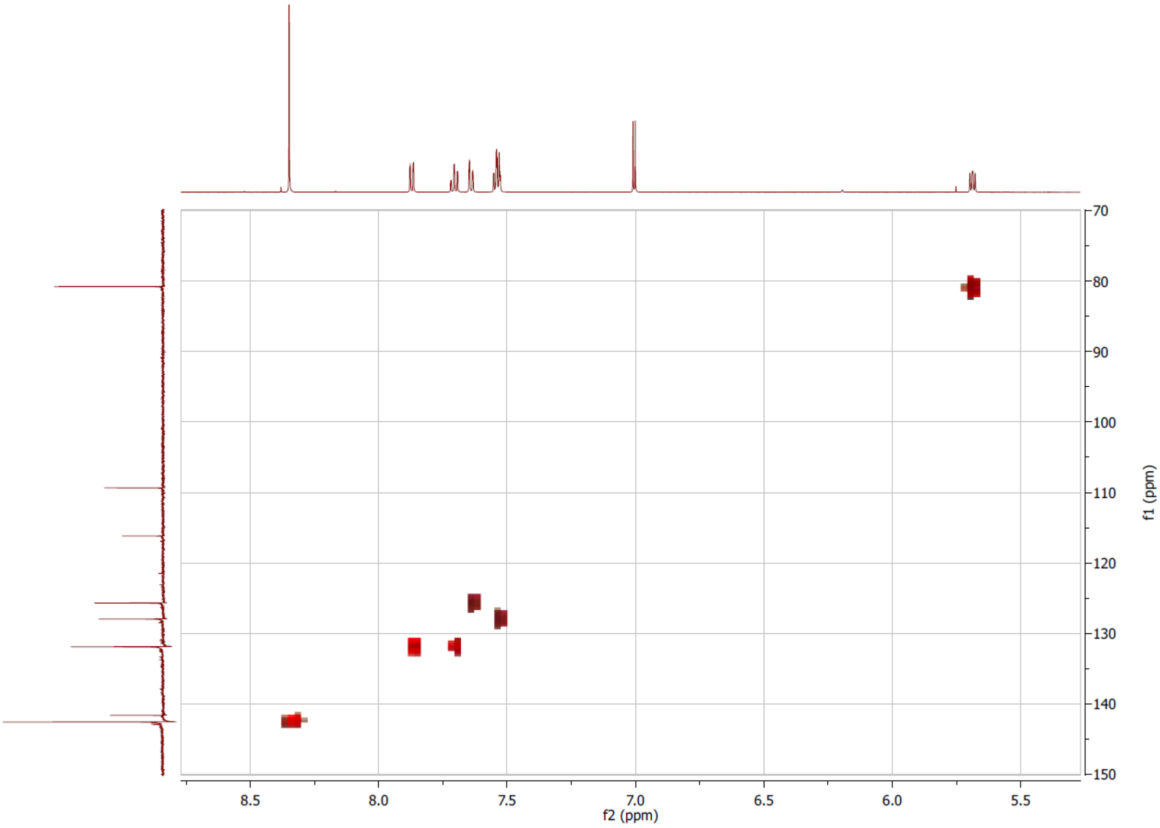

HMBC

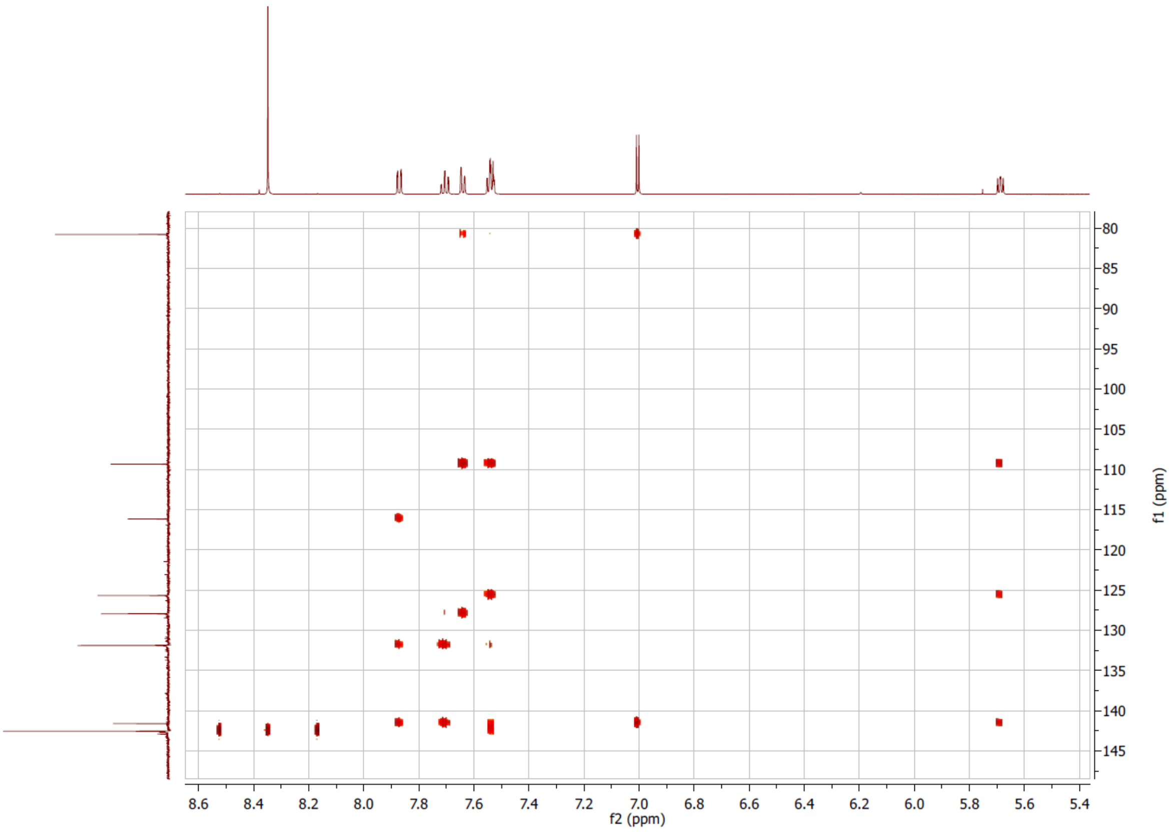

3.1.5. IR Spectroscopy

IR (KBr, cm^{-1}): 3191vs, 3110vs, 3082vs, 2994s, 2854m, 2228s, 1942vw, 1700vw, 1669vw, 1653w, 1636w, 1601w, 1580vw, 1554s, 1504m, 1487w, 1450w, 1356w, 1335w, 1313m, 1287vw, 1268w, 1211s, 1184m, 1161vw, 1111m, 1090vw, 1071vs, 1052vs, 1038m, 976m, 953w, 898m, 880m, 837m, 784w, 758vs, 696m, 639vs, 618m, 575w, 560s, 496w, 473vw, 414vw, 382vw.

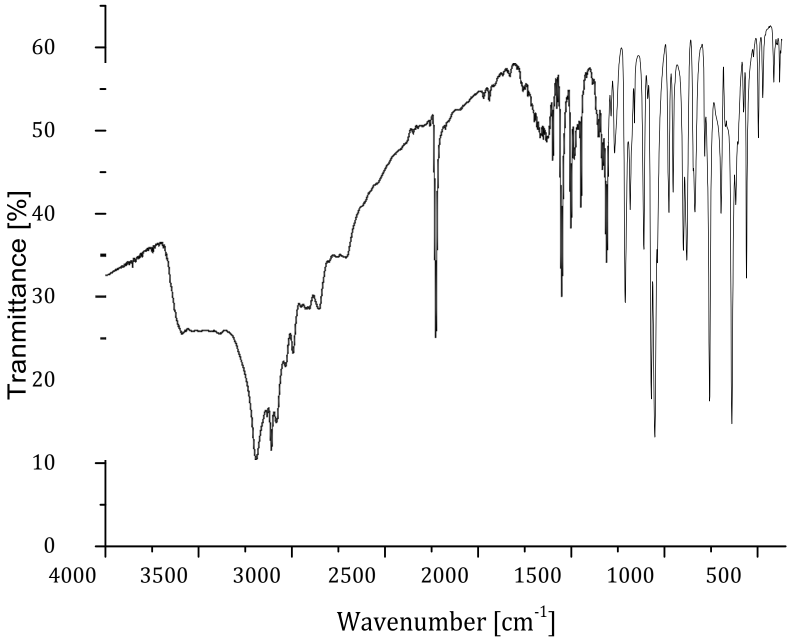

3.1.6. Crystallography

2-[hydroxy(4H-1,2,4-triazol-4-ylamino)methyl]benzonitrile acetonitrile solvate (1a).

Figure 1. Molecular structure and labelling for 2-[hydroxy(4*H*-1,2,4-triazol-4-ylamino)methyl]benzonitrile acetonitrile solvate (1a). Displacement ellipsoids are shown at the 50% probability level.

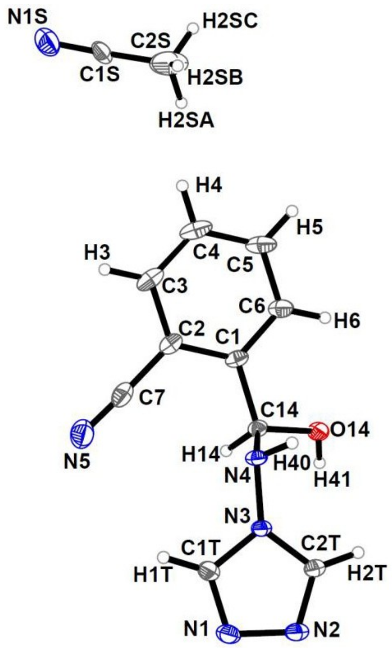

Table 1. Crystal data and structure refinement for 1a.

Identification code	1a	
Empirical formula	C22 H21 N11 O2	
Formula weight	471.50	
Temperature	100(2) K	
Wavelength	0.71073 Å	
Crystal system	Monoclinic	
Space group	C 1 2/c 1	
Unit cell dimensions	a = 24.683(6) Å	α = 90°.
	b = 10.509(3) Å	β = 95.23(3)°.
	c = 8.826(3) Å	γ = 90°.
Volume	2279.9(12) $Å^3$	
Z	4	
Density (calculated)	1.374 Mg/m^3	
Absorption coefficient	0.096 mm^{-1}	
F(000)	984	
Crystal size	0.52 × 0.42 × 0.15 mm^3	
Theta range for data collection	4.74 to 38.51°	
Index ranges	−43 <= h <= 42, −16 <= k <= 18, −15 <= l <= 15	
Reflections collected	21879	
Independent reflections	6177 [R(int) = 0.0233]	
Completeness to theta = 27.00°	99.5%	

Table 1. *Cont.*

Absorption correction	Semi-empirical from equivalents
Max. and min. transmission	1.00000 and 0.91466
Refinement method	Full-matrix least-squares on F^2
Data/restraints/parameters	6177/0/179
Goodness-of-fit on F^2	1.010
Final R indices [I > 2sigma(I)]	R1 = 0.0420, wR2 = 0.1139
R indices (all data)	R1 = 0.0676, wR2 = 0.1216
Largest diff. peak and hole	0.445 and −0.275 e.Å^{-3}

Table 2. Atomic coordinates (×10^4) and equivalent isotropic displacement parameters (Å^2 × 10^3) for 1a. U(eq) is defined as one third of the trace of the orthogonalized U^{ij} tensor.

	**x**	**y**	**z**	**U(eq)**
C(1)	3724(1)	5729(1)	6910(1)	17(1)
C(2)	4176(1)	5577(1)	7975(1)	19(1)
C(3)	4373(1)	4364(1)	8383(1)	27(1)
C(4)	4114(1)	3303(1)	7746(1)	30(1)
C(5)	3668(1)	3442(1)	6693(1)	28(1)
C(6)	3477(1)	4648(1)	6265(1)	22(1)
C(7)	4448(1)	6669(1)	8683(1)	24(1)
N(5)	4676(1)	7515(1)	9266(1)	33(1)
C(14)	3505(1)	7051(1)	6539(1)	15(1)
O(14)	3159(1)	7033(1)	5192(1)	19(1)
N(4)	3230(1)	7442(1)	7877(1)	16(1)
N(3)	3104(1)	8749(1)	7849(1)	15(1)
C(1T)	3370(1)	9616(1)	8770(1)	20(1)
N(1)	3168(1)	10752(1)	8492(1)	21(1)
N(2)	2749(1)	10627(1)	7334(1)	20(1)
C(2T)	2721(1)	9417(1)	6969(1)	19(1)
C(2S)	4726(3)	50(7)	7175(9)	52(2)
C(1S)	5269(2)	−15(6)	7944(6)	24(1)
N(1S)	5709(1)	−103(2)	8608(2)	39(1)

Table 3. Bond lengths [Å] and angles [°] for 1a.

C(1)-C(6)	1.3861(10)
C(1)-C(2)	1.4016(11)
C(1)-C(14)	1.5162(9)
C(2)-C(3)	1.3996(10)
C(2)-C(7)	1.4422(11)
C(3)-C(4)	1.3795(13)
C(4)-C(5)	1.3824(14)
C(5)-C(6)	1.3926(10)
C(7)-N(5)	1.1482(11)
C(14)-O(14)	1.3993(10)
C(14)-N(4)	1.4738(10)
N(4)-N(3)	1.4075(8)

Table 3. *Cont.*

N(3)-C(1T)	1.3512(9)
N(3)-C(2T)	1.3623(9)
C(1T)-N(1)	1.3090(9)
N(1)-N(2)	1.3923(10)
N(2)-C(2T)	1.3123(9)
C(2S)-C(1S)	1.448(3)
C(1S)-N(1S)	1.190(5)
C(6)-C(1)-C(2)	118.37(6)
C(6)-C(1)-C(14)	121.84(7)
C(2)-C(1)-C(14)	119.74(6)
C(3)-C(2)-C(1)	120.87(7)
C(3)-C(2)-C(7)	118.47(7)
C(1)-C(2)-C(7)	120.67(6)
C(4)-C(3)-C(2)	119.60(8)
C(3)-C(4)-C(5)	120.04(7)
C(4)-C(5)-C(6)	120.43(7)
C(1)-C(6)-C(5)	120.68(8)
N(5)-C(7)-C(2)	177.97(8)
O(14)-C(14)-N(4)	113.20(6)
O(14)-C(14)-C(1)	110.44(5)
N(4)-C(14)-C(1)	105.28(5)
N(3)-N(4)-C(14)	111.99(5)
C(1T)-N(3)-C(2T)	105.62(6)
C(1T)-N(3)-N(4)	123.63(6)
C(2T)-N(3)-N(4)	130.75(6)
N(1)-C(1T)-N(3)	110.28(7)
C(1T)-N(1)-N(2)	107.23(6)
C(2T)-N(2)-N(1)	106.91(6)
N(2)-C(2T)-N(3)	109.96(6)
N(1S)-C(1S)-C(2S)	177.7(7)

Table 4. Anisotropic displacement parameters ($Å^2 \times 10^3$) for 1a. The anisotropic displacement factor exponent takes the form: $-2\pi^2[h^2a^{*2}U^{11} + ... + 2\ h\ k\ a^*\ b^*\ U^{12}]$.

	**U11**	**U22**	**U33**	**U23**	**U13**	**U12**
C(1)	22(1)	14(1)	14(1)	3(1)	4(1)	5(1)
C(2)	20(1)	20(1)	19(1)	4(1)	3(1)	6(1)
C(3)	26(1)	26(1)	29(1)	9(1)	4(1)	12(1)
C(4)	40(1)	19(1)	32(1)	6(1)	9(1)	13(1)
C(5)	44(1)	14(1)	26(1)	0(1)	7(1)	6(1)
C(6)	32(1)	15(1)	18(1)	0(1)	2(1)	4(1)
C(7)	20(1)	28(1)	24(1)	9(1)	−1(1)	4(1)
N(5)	29(1)	34(1)	35(1)	9(1)	−7(1)	−5(1)
C(14)	19(1)	13(1)	14(1)	2(1)	0(1)	2(1)

Table 4. *Cont.*

	U^{11}	U^{22}	U^{33}	U^{23}	U^{13}	U^{12}
O(14)	28(1)	14(1)	14(1)	4(1)	−4(1)	0(1)
N(4)	20(1)	10(1)	17(1)	1(1)	2(1)	2(1)
N(3)	18(1)	11(1)	17(1)	0(1)	0(1)	2(1)
C(1T)	24(1)	16(1)	19(1)	−2(1)	−2(1)	0(1)
N(1)	26(1)	14(1)	22(1)	−2(1)	1(1)	0(1)
N(2)	24(1)	13(1)	24(1)	−1(1)	1(1)	3(1)
C(2T)	21(1)	14(1)	21(1)	0(1)	−2(1)	3(1)
C(2S)	47(3)	27(2)	84(4)	8(2)	18(3)	4(2)
C(1S)	18(1)	20(1)	33(1)	−10(1)	1(1)	−3(1)
N(1S)	38(1)	33(1)	44(1)	−13(1)	−7(1)	−3(1)

Table 5. Hydrogen coordinates (×10^4) and isotropic displacement parameters (Å^2 r 10^3) for 1a.

	x	y	z	U(eq)
H(3)	4683	4272	9095	32
H(4)	4242	2477	8031	36
H(5)	3490	2709	6258	33
H(6)	3175	4732	5524	26
H(14)	3816	7641	6414	19
H(41)	3193(4)	7721(10)	4695(11)	29
H(40)	2921(4)	7026(8)	7855(10)	19
H(1T)	3662	9428	9513	24
H(2T)	2471	9059	6203	22
H(2SA)	4701	782	6483	78
H(2SB)	4461	146	7928	78
H(2SC)	4649	−733	6591	78

Table 6. Torsion angles [°] for 1a.

C(6)-C(1)-C(2)-C(3)	−0.24(10)
C(14)-C(1)-C(2)-C(3)	177.40(6)
C(6)-C(1)-C(2)-C(7)	−179.88(7)
C(14)-C(1)-C(2)-C(7)	−2.23(10)
C(1)-C(2)-C(3)-C(4)	−0.95(11)
C(7)-C(2)-C(3)-C(4)	178.70(7)
C(2)-C(3)-C(4)-C(5)	0.99(12)
C(3)-C(4)-C(5)-C(6)	0.14(13)
C(2)-C(1)-C(6)-C(5)	1.38(11)
C(14)-C(1)-C(6)-C(5)	−176.21(7)
C(4)-C(5)-C(6)-C(1)	−1.36(12)
C(6)-C(1)-C(14)-O(14)	−18.36(9)
C(2)-C(1)-C(14)-O(14)	164.08(6)
C(6)-C(1)-C(14)-N(4)	104.15(7)
C(2)-C(1)-C(14)-N(4)	−73.40(8)
O(14)-C(14)-N(4)-N(3)	−71.87(7)
C(1)-C(14)-N(4)-N(3)	167.41(5)

Table 6. *Cont.*

C(14)-N(4)-N(3)-C(1T)	−107.20(7)
C(14)-N(4)-N(3)-C(2T)	72.50(9)
C(2T)-N(3)-C(1T)-N(1)	0.18(8)
N(4)-N(3)-C(1T)-N(1)	179.94(6)
N(3)-C(1T)-N(1)-N(2)	−0.03(8)
C(1T)-N(1)-N(2)-C(2T)	−0.15(8)
N(1)-N(2)-C(2T)-N(3)	0.27(8)
C(1T)-N(3)-C(2T)-N(2)	−0.28(8)
N(4)-N(3)-C(2T)-N(2)	179.99(6)

Table 7. Hydrogen bonds for 1a [Å and °].

D-H...A	d(D-H)	d(H...A)	d(D...A)	<(DHA)
O(14)-H(41)...N(1)#1	0.853(11)	1.922(11)	2.7700(10)	172.4(10)
N(4)-H(40)...N(2)#2	0.876(9)	2.208(9)	3.0695(10)	167.6(8)
C(1T)-H(1T)...N(1S)#3	0.95	2.28	3.135(2)	149.5
C(2T)-H(2T)...O(14)#4	0.95	2.21	3.1494(12)	168.1

Symmetry transformations used to generate equivalent atoms: #1 x, −y + 2, z − 1/2; #2 −x + 1/2, y − 1/2, −z + 3/2; #3 −x + 1, −y + 1, −z + 2; #4 −x + 1/2, −y + 3/2, −z + 1.

2-[hydroxy(4H-1,2,4-triazol-4-ylamino)methyl]benzonitrile (1b)

Figure 2. Molecular structure and labelling for 2-[hydroxy(4*H*-1,2,4-triazol-4-ylamino)methyl]benzonitrile (1b). Displacement ellipsoids are shown at the 50% probability level.

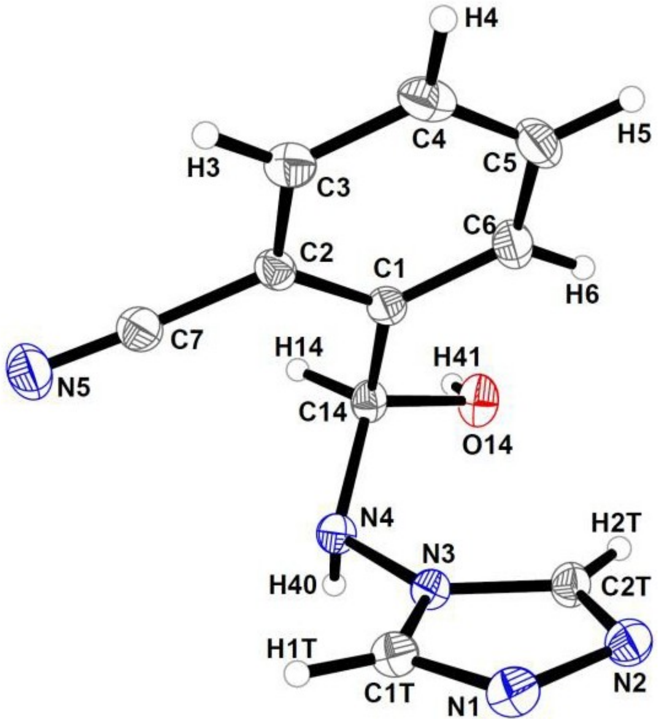

Table 1. Crystal data and structure refinement for 1b.

Identification code	1b	
Empirical formula	C10 H9 N5 O	
Formula weight	215.22	
Temperature	100(2) K	
Wavelength	0.71073 Å	
Crystal system	Monoclinic	
Space group	P 1 21/n 1	
Unit cell dimensions	a = 7.574(3) Å	α = 90°
	b = 17.464(5) Å	β = 91.01(3)°
	c = 7.908(3) Å	γ = 90°
Volume	1045.8(6) $Å^3$	
Z	4	
Density (calculated)	1.367 Mg/m^3	
Absorption coefficient	0.096 mm^{-1}	
F(000)	448	
Crystal size	0.41 × 0.27 × 0.21 mm^3	
Theta range for data collection	2.93 to 36.93°.	
Index ranges	−9 <= h <= 10, −27 <= k <= 21, −10 <= l<= 13	
Reflections collected	10163	
Independent reflections	3550 [R(int) = 0.0257]	
Completeness to theta = 27.00°	99.9%	
Absorption correction	Semi-empirical from equivalents	
Max. and min. transmission	1.00000 and 0.98054	
Refinement method	Full-matrix least-squares on F2	
Data/restraints/parameters	3550/0/151	
Goodness-of-fit on F2	1.079	
Final R indices [I > 2sigma(I)]	R1 = 0.0421, wR2 = 0.1069	
R indices (all data)	R1 = 0.0629, wR2 = 0.1118	
Largest diff. peak and hole	0.426 and −0.187 e.$Å^{-3}$	

Table 2. Atomic coordinates ($\times 10^4$) and equivalent isotropic displacement parameters ($Å^2 \times 10^3$) for 4. U(eq) is defined as one third of the trace of the orthogonalized U^{ij} tensor.

	**x**	**y**	**z**	**U(eq)**
C(1)	6425(1)	4137(1)	1088(1)	16(1)
C(2)	7127(1)	4869(1)	1409(1)	17(1)
C(3)	7846(1)	5304(1)	110(1)	21(1)
C(4)	7891(1)	5009(1)	−1515(1)	23(1)
C(5)	7219(2)	4287(1)	−1845(1)	24(1)
C(6)	6489(1)	3857(1)	−557(1)	22(1)
C(7)	7123(1)	5209(1)	3072(1)	20(1)
N(5)	7174(1)	5518(1)	4355(1)	29(1)
C(14)	5601(1)	3660(1)	2475(1)	17(1)
O(14)	4787(1)	3000(1)	1765(1)	23(1)
N(4)	6935(1)	3463(1)	3771(1)	16(1)
N(3)	8429(1)	3104(1)	3082(1)	15(1)
C(1T)	10097(1)	3342(1)	3407(1)	18(1)
N(1)	11216(1)	2965(1)	2488(1)	21(1)
N(2)	10235(1)	2454(1)	1494(1)	20(1)
C(2T)	8578(1)	2546(1)	1879(1)	19(1)
C(2T)	8578(1)	2546(1)	1879(1)	19(1)

Table 3. Bond lengths [Å] and angles [°] for 1b.

C(1)-C(6)	1.3917(14)
C(1)-C(2)	1.4067(14)
C(1)-C(14)	1.5196(15)
C(2)-C(3)	1.3962(15)
C(2)-C(7)	1.4428(15)
C(3)-C(4)	1.3856(15)
C(3)-H(3)	0.9500
C(4)-C(5)	1.3822(16)
C(4)-H(4)	0.9500
C(5)-C(6)	1.3881(16)
C(5)-H(5)	0.9500
C(6)-H(6)	0.9500
C(7)-N(5)	1.1490(14)
C(14)-O(14)	1.4182(12)
C(14)-N(4)	1.4677(13)
C(14)-H(14)	1.0000
O(14)-H(41)	0.928(16)
N(4)-N(3)	1.4115(12)
N(4)-H(40)	0.911(12)
N(3)-C(1T)	1.3509(13)
N(3)-C(2T)	1.3684(13)
C(1T)-N(1)	1.3053(14)
C(1T)-H(1T)	0.9500
N(1)-N(2)	1.3940(12)
N(2)-C(2T)	1.3064(15)
C(2T)-H(2T)	0.9500
C(6)-C(1)-C(2)	117.94(10)
C(6)-C(1)-C(14)	120.29(9)
C(2)-C(1)-C(14)	121.78(9)
C(3)-C(2)-C(1)	120.85(9)
C(3)-C(2)-C(7)	117.02(9)
C(1)-C(2)-C(7)	122.12(9)
C(4)-C(3)-C(2)	119.77(10)
C(4)-C(3)-H(3)	120.1
C(2)-C(3)-H(3)	120.1
C(5)-C(4)-C(3)	119.97(10)
C(5)-C(4)-H(4)	120.0
C(3)-C(4)-H(4)	120.0
C(4)-C(5)-C(6)	120.31(10)
C(4)-C(5)-H(5)	119.8
C(6)-C(5)-H(5)	119.8
C(5)-C(6)-C(1)	121.15(10)
C(5)-C(6)-H(6)	119.4
C(1)-C(6)-H(6)	119.4
N(5)-C(7)-C(2)	175.78(11)
O(14)-C(14)-N(4)	111.99(8)

Table 3. *Cont.*

O(14)-C(14)-C(1)	109.92(8)
N(4)-C(14)-C(1)	110.24(8)
O(14)-C(14)-H(14)	108.2
N(4)-C(14)-H(14)	108.2
C(1)-C(14)-H(14)	108.2
C(14)-O(14)-H(41)	108.2(9)
N(3)-N(4)-C(14)	112.43(8)
N(3)-N(4)-H(40)	109.1(8)
C(14)-N(4)-H(40)	109.4(8)
C(1T)-N(3)-C(2T)	105.29(8)
C(1T)-N(3)-N(4)	122.88(8)
C(2T)-N(3)-N(4)	131.43(8)
N(1)-C(1T)-N(3)	110.65(9)
N(1)-C(1T)-H(1T)	124.7
N(3)-C(1T)-H(1T)	124.7
C(1T)-N(1)-N(2)	106.97(9)
C(2T)-N(2)-N(1)	107.20(9)
N(2)-C(2T)-N(3)	109.89(9)
N(2)-C(2T)-H(2T)	125.1
N(3)-C(2T)-H(2T)	125.1

Table 4. Anisotropic displacement parameters ($Å^2 \times 10^3$) for 1b. The anisotropic displacement factor exponent takes the form: $-2\pi^2[h^2a^{*2}U^{11} + ... + 2\ h\ k\ a^*\ b^*\ U^{12}]$.

	**U^{11}**	**U^{22}**	**U^{33}**	**U^{23}**	**U^{13}**	**U^{12}**
C(1)	13(1)	19(1)	17(1)	−2(1)	−1(1)	3(1)
C(2)	15(1)	21(1)	15(1)	−1(1)	−2(1)	2(1)
C(3)	21(1)	22(1)	21(1)	2(1)	−2(1)	1(1)
C(4)	22(1)	31(1)	18(1)	6(1)	1(1)	3(1)
C(5)	26(1)	32(1)	15(1)	−3(1)	0(1)	5(1)
C(6)	22(1)	24(1)	19(1)	−5(1)	−1(1)	1(1)
C(7)	22(1)	20(1)	20(1)	1(1)	−1(1)	−2(1)
N(5)	37(1)	26(1)	23(1)	−4(1)	−2(1)	−5(1)
C(14)	14(1)	18(1)	19(1)	−3(1)	1(1)	0(1)
O(14)	15(1)	24(1)	30(1)	−8(1)	2(1)	−4(1)
N(4)	14(1)	19(1)	15(1)	0(1)	4(1)	1(1)
N(3)	14(1)	16(1)	16(1)	0(1)	1(1)	0(1)
C(1T)	16(1)	18(1)	21(1)	1(1)	−1(1)	−2(1)
N(1)	18(1)	20(1)	25(1)	1(1)	0(1)	−2(1)
N(2)	18(1)	21(1)	21(1)	−1(1)	0(1)	2(1)
C(2T)	18(1)	18(1)	20(1)	−3(1)	−1(1)	1(1)

Table 5. Hydrogen coordinates (×10^4) and isotropic displacement parameters (Å^2 × 10^3) for 1b.

	x	y	z	U(eq)
H(3)	8302	5801	338	25
H(4)	8383	5302	−2402	28
H(5)	7257	4085	−2959	29
H(6)	6024	3364	−804	26
H(14)	4666	3976	3020	20
H(41)	3630(20)	2983(8)	2125(18)	34
H(40)	6447(16)	3145(7)	4549(15)	20
H(1T)	10412	3731	4196	22
H(2T)	7619	2266	1395	22

Table 6. Torsion angles [°] for 1b.

C(6)-C(1)-C(2)-C(3)	0.73(14)
C(14)-C(1)-C(2)-C(3)	−178.65(9)
C(6)-C(1)-C(2)-C(7)	179.96(9)
C(14)-C(1)-C(2)-C(7)	0.58(14)
C(1)-C(2)-C(3)-C(4)	−0.82(15)
C(7)-C(2)-C(3)-C(4)	179.91(9)
C(2)-C(3)-C(4)-C(5)	0.27(15)
C(3)-C(4)-C(5)-C(6)	0.36(16)
C(4)-C(5)-C(6)-C(1)	−0.44(16)
C(2)-C(1)-C(6)-C(5)	−0.10(15)
C(14)-C(1)-C(6)-C(5)	179.29(9)
C(6)-C(1)-C(14)-O(14)	−7.43(12)
C(2)-C(1)-C(14)-O(14)	171.94(8)
C(6)-C(1)-C(14)-N(4)	116.50(10)
C(2)-C(1)-C(14)-N(4)	−64.14(12)
O(14)-C(14)-N(4)-N(3)	68.73(10)
C(1)-C(14)-N(4)-N(3)	−53.99(10)
C(14)-N(4)-N(3)-C(1T)	128.51(10)
C(14)-N(4)-N(3)-C(2T)	−43.06(14)
C(2T)-N(3)-C(1T)-N(1)	−0.19(11)
N(4)-N(3)-C(1T)-N(1)	−173.65(8)
N(3)-C(1T)-N(1)-N(2)	0.51(11)
C(1T)-N(1)-N(2)-C(2T)	−0.65(11)
N(1)-N(2)-C(2T)-N(3)	0.54(12)
C(1T)-N(3)-C(2T)-N(2)	−0.24(11)
N(4)-N(3)-C(2T)-N(2)	172.43(9)

Table 7. Hydrogen bonds for 1b [Å and °].

D-H...A	d(D-H)	d(H...A)	d(D...A)	<(DHA)
N(4)-H(40)...N(2)#1	0.911(12)	2.087(13)	2.9928(15)	172.6(11)
O(14)-H(41)...N(1)#2	0.928(16)	1.855(16)	2.7746(16)	171.0(13)
C(1T)-H(1T)...N(5)#3	0.95	2.51	3.3523(16)	147.8

Symmetry transformations used to generate equivalent atoms: #1 x − 1/2, −y + 1/2, z + 1/2; #2 x − 1, y, z; #3 −x + 2, −y + 1, −z + 1.

3.2. 2-[(E)-(4H-1,2,4-triazol-4-ylimino)methyl]benzonitrile (1s)

3.2.1. Synthesis

Ethanolic solution (3 mL) of 2-formylbenzonitrile (51 mg) was added to an ethanolic solution (3 mL) of 4-amino-1,2,4-triazole (33 mg). Few drops of hydrochloric acid were added to the obtained solution. The reaction mixture after complete dissolution was refluxed for 4 hours. The title compound crystallised directly from the mother liquor. Upon standing 4 days at the room temperature, the solution deposited pale yellow crystal needles. The crystals were filtered off, washed with a small amount of ethanol and diethyl ether then dried in the air to afford 2-[(*E*)-(4*H*-1,2,4-triazol-4-ylimino)methyl]benzonitrile—(37 mg, 48%), mp 197 °C.

3.2.2. Elemental Analysis

	% C	% H	% N
Calculated	60.91	3.58	35.51
Found	60.78	3.47	35.25

3.2.3. Mass Spectrometry

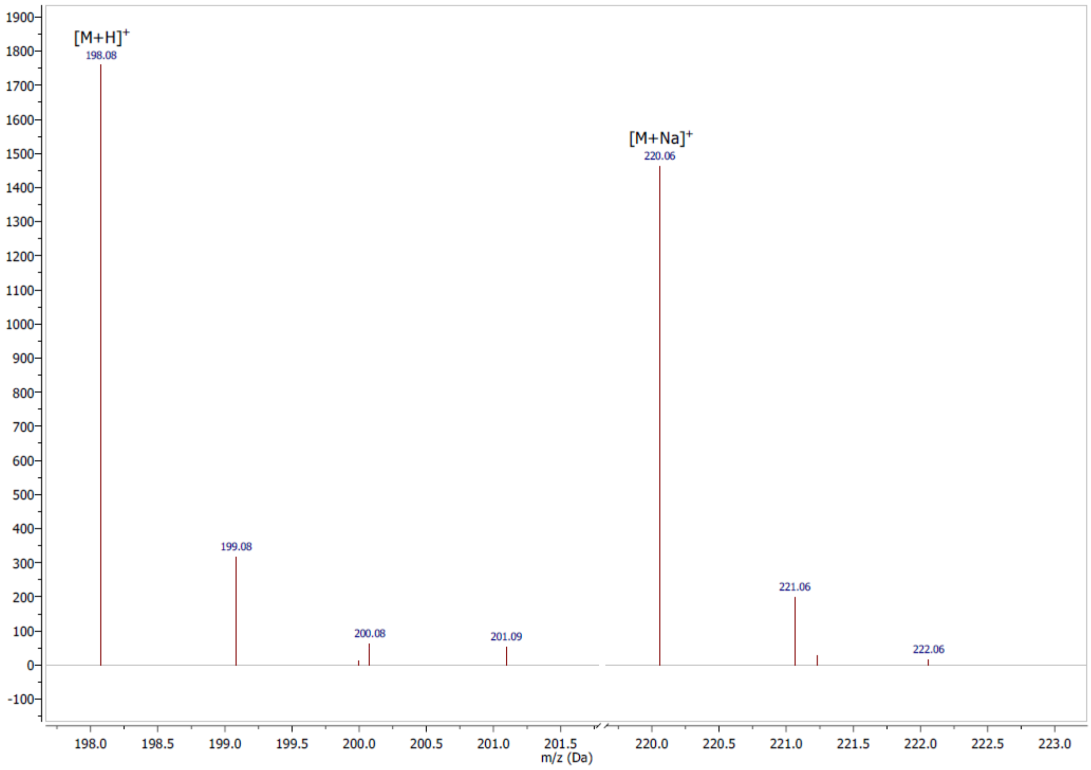

3.2.4. NMR Spectroscopy

^{1}H-NMR

^{1}H-NMR (500 MHz, DMSO, RT): 9.22 (s, 2H, H1T, H2T), 9.16 (s, 1H, H14), 8.08–8.10 (m, 1H, H6), 8.04–8.06 (m, 1H, H3), 7.88–7.92 (m, 1H, H4), 7.77–7.80 (m, 1H, H5).

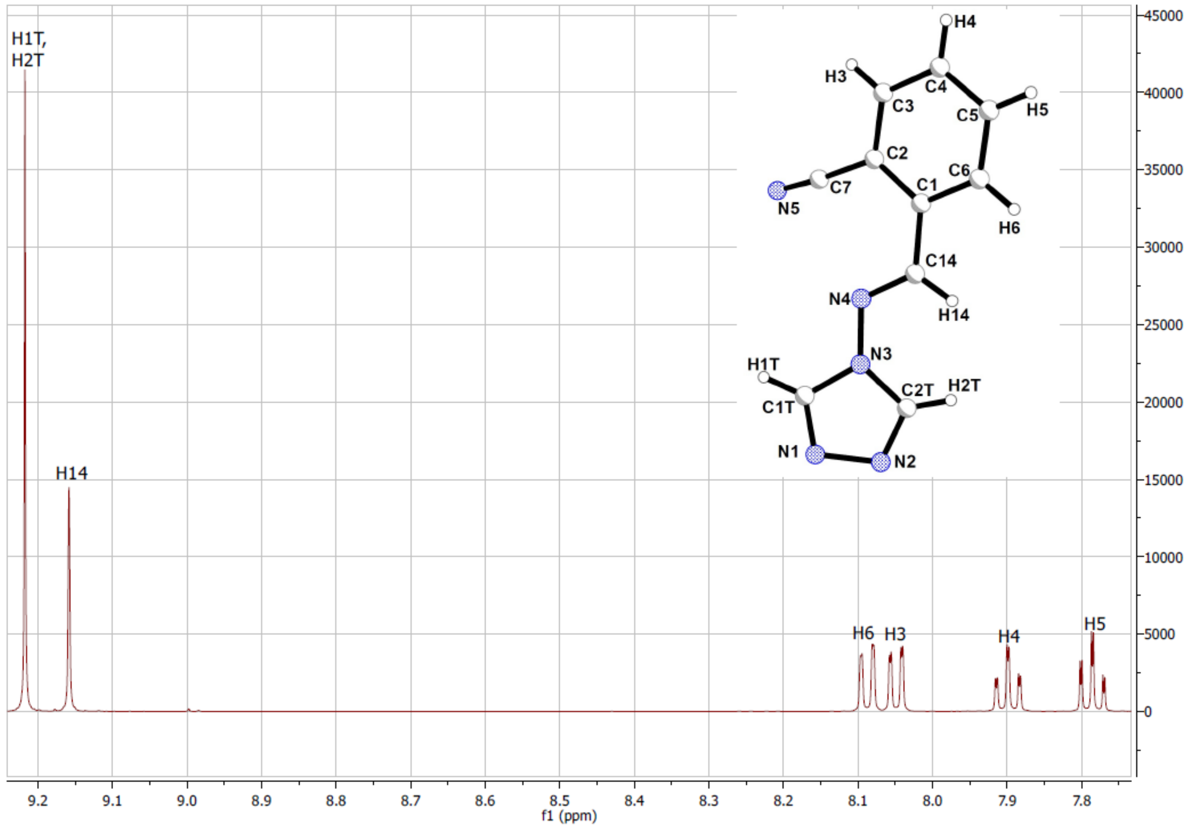

^{13}C-NMR

^{13}C-NMR (125.8 MHz, DMSO, RT): 154.4 (C14), 139.1 (C1T, C2T), 134.7 (C3), 134.0 (C2), 133.7 (C4), 132.4 (C5), 129.2 (C6), 116.8 (C7), 111.0 (C1).

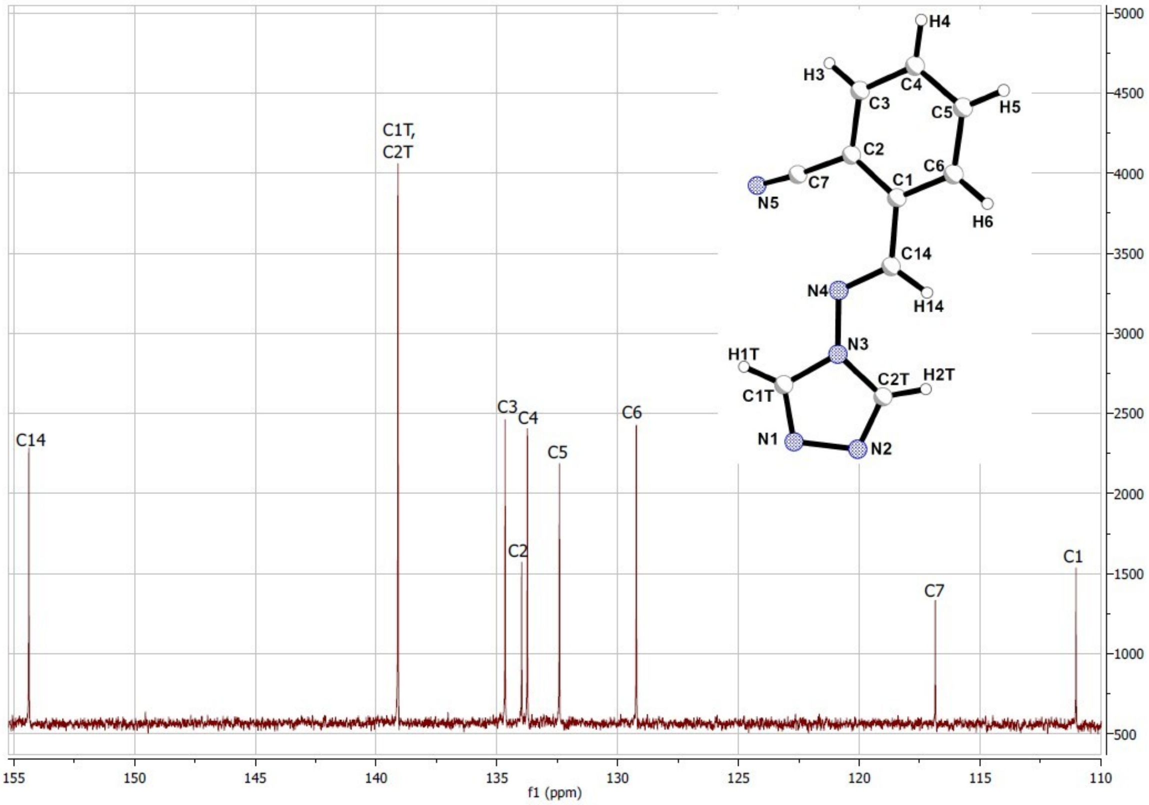

HSQC

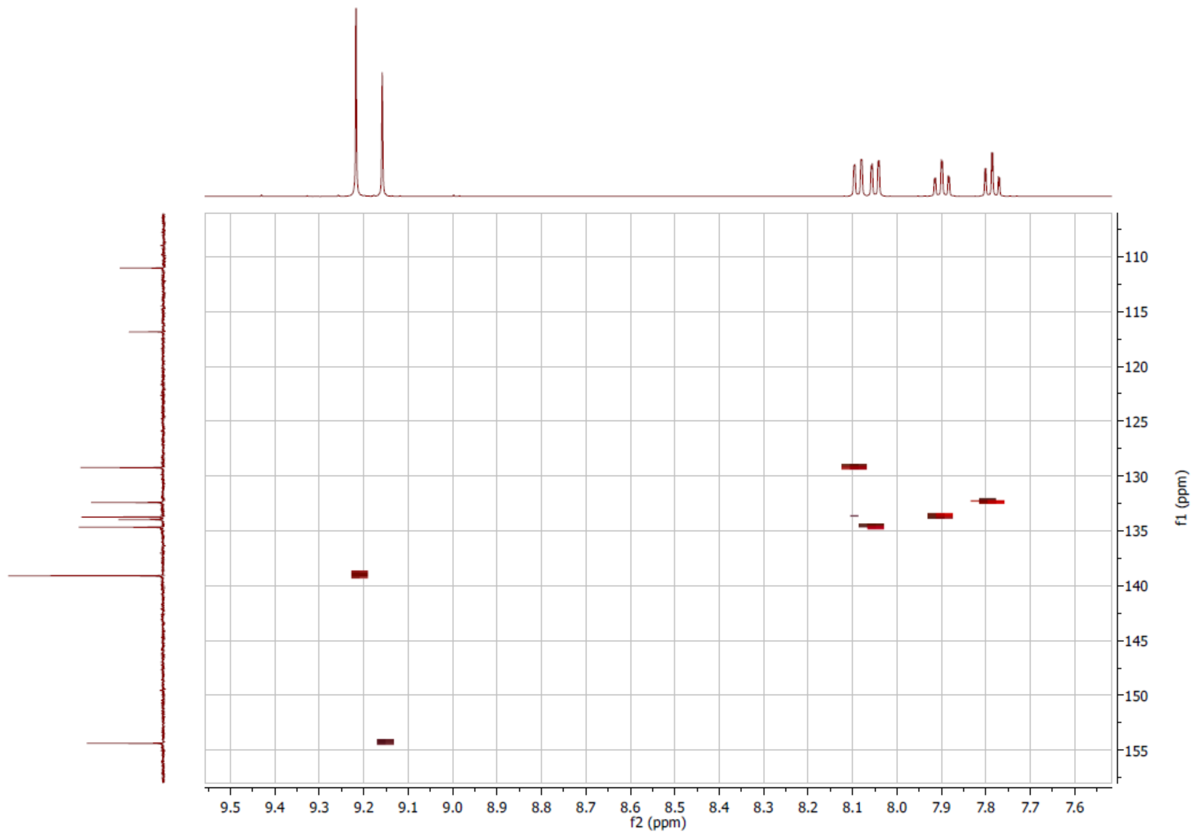

9.5
9.4
9.3
9.2
9.1
9.0
8.9
8.8
8.7
8.6
8.5
8.4
8.3
8.2
8.1
8.0
7.9
7.8
7.7
7.6
f2 (ppm)
110
115
120
125
130
135
140
145
150
155
f1 (ppm)

HMBC

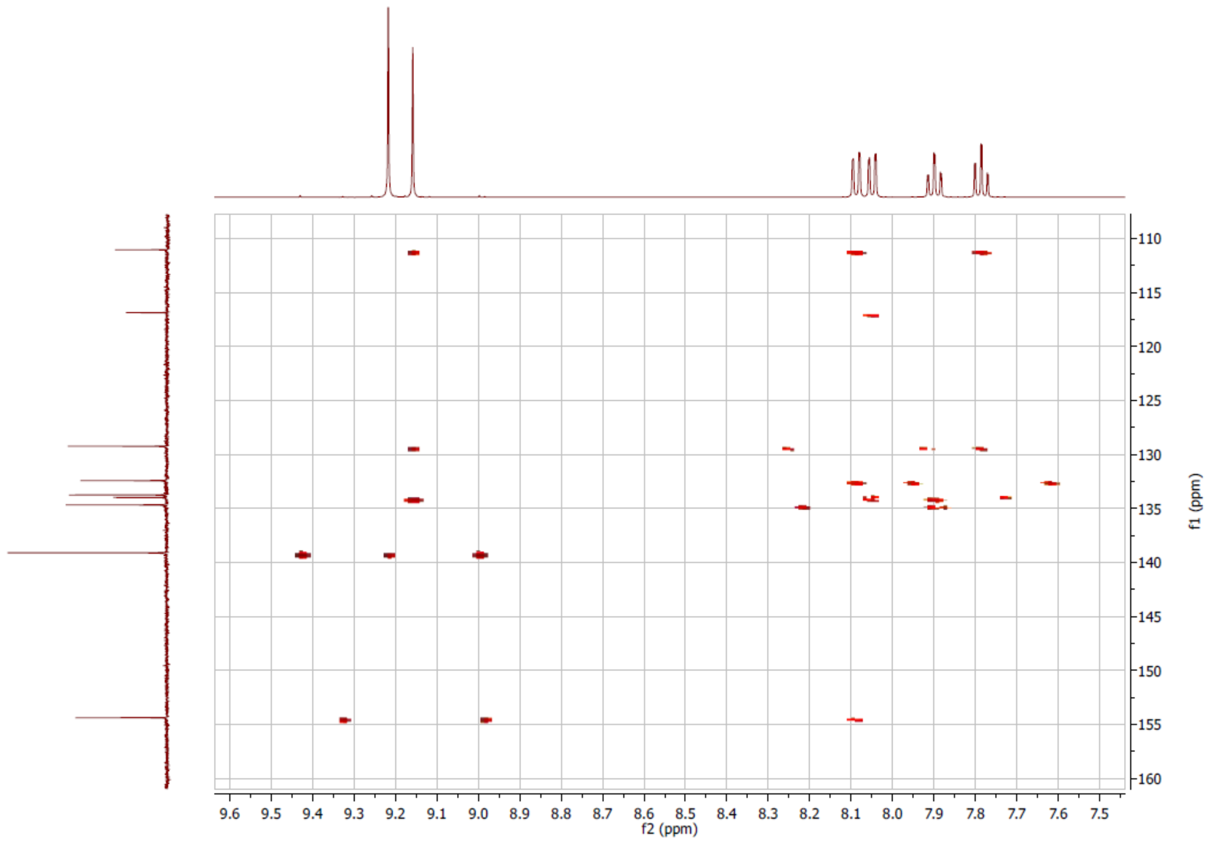

9.6
9.5
9.4
9.3
9.2
9.1
9.0
8.9
8.8
8.7
8.6
8.5
8.4
8.3
8.2
8.1
8.0
7.9
7.8
7.7
7.6
7.5
f2 (ppm)
110
115
120
125
130
135
140
145
150
155
160
f1 (ppm)

3.2.5. IR Spectroscopy

IR (KBr, cm^{-1}): 3438m, 3141m, 3082s, 3033m, 2959m, 2931m, 2225m, 1717w, 1696w, 1624w, 1592w, 1569w, 1525m, 1507vs, 1491s, 1469m, 1440w, 1397w, 1346vw, 1328vw, 1315w, 1300m, 1277w, 1218s, 1163vs, 1056vs, 999w, 978s, 959w, 940m, 899w, 890w, 873m, 849w, 774vs, 766vs, 734w, 718vw, 671vw, 623vs, 614s, 567w, 560s, 510m, 505m, 459vw.

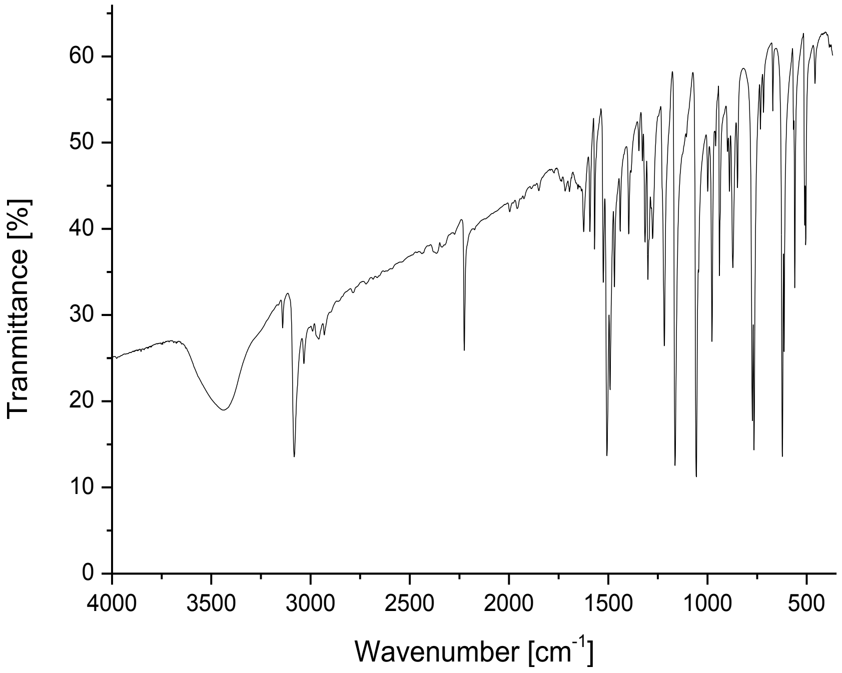

3.2.6. Crystallography

Figure 3. Molecular structure and labelling for 2-[(*E*)-(4*H*-1,2,4-triazol-4-ylimino)methyl]benzonitrile (1s). Displacement ellipsoids are shown at the 50% probability level.

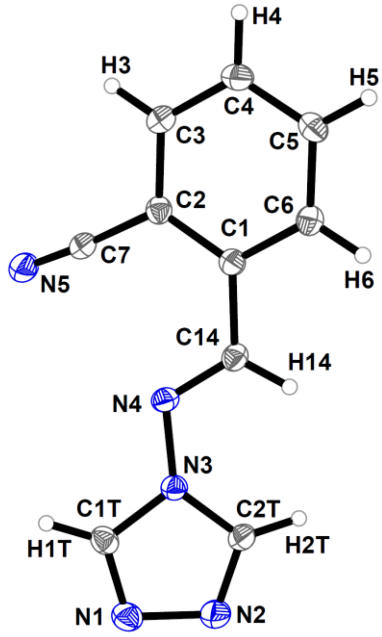

Table 1. Crystal data and structure refinement for 1s.

Identification code	1s	
Empirical formula	C10 H7 N5	
Formula weight	197.21	
Temperature	100(2) K	
Wavelength	0.71073 Å	
Crystal system	Monoclinic	
Space group	P 1 21/n 1	
Unit cell dimensions	a = 3.969(2) Å	α = 90°.
	b = 28.422(6) Å	β = 103.31(3)°.
	c = 8.300(3) Å	γ = 90°.
Volume	911.1(6) $Å^3$	
Z	4	
Density (calculated)	1.438 Mg/m^3	
Absorption coefficient	0.096 mm^{-1}	
F(000)	408	
Crystal size	0.47 × 0.29 × 0.20 mm^3	
Theta range for data collection	2.87 to 28.84°.	
Index ranges	−5 <= h <= 4, −38 <= k <= 36, −8 <= l <= 11	
Reflections collected	6590	
Independent reflections	2223 [R(int) = 0.0168]	
Completeness to theta = 27.00°	99.7%	
Absorption correction	Semi-empirical from equivalents	
Max. and min. transmission	1.00000 and 0.96905	
Refinement method	Full-matrix least-squares on F^2	
Data/restraints/parameters	2223/0/136	
Goodness-of-fit on F^2	0.947	
Final R indices [I > 2sigma(I)]	R1 = 0.0363, wR2 = 0.1135	
R indices (all data)	R1 = 0.0436, wR2 = 0.1191	
Largest diff. peak and hole	0.357 and −0.166 e.$Å^{-3}$	

Table 2. Atomic coordinates ($\times 10^4$) and equivalent isotropic displacement parameters ($Å^2 \times 10^3$) for 1s. U(eq) is defined as one third of the trace of the orthogonalized U^{ij} tensor.

	x	y	z	U(eq)
C(1)	5239(3)	1196(1)	7411(1)	17(1)
C(2)	3887(3)	757(1)	6778(1)	18(1)
C(3)	2777(3)	432(1)	7795(1)	21(1)
C(4)	2998(3)	540(1)	9447(1)	21(1)
C(5)	4275(3)	972(1)	10077(1)	21(1)
C(6)	5353(3)	1299(1)	9059(1)	19(1)
C(7)	3440(3)	630(1)	5053(1)	21(1)

Table 2. *Cont.*

	x	y	z	U(eq)
N(5)	2908(3)	508(1)	3699(1)	28(1)
C(14)	6575(3)	1556(1)	6457(1)	18(1)
N(4)	7204(2)	1455(1)	5063(1)	18(1)
N(3)	8447(2)	1819(1)	4237(1)	17(1)
C(1T)	9731(3)	1732(1)	2886(1)	20(1)
N(1)	10565(2)	2122(1)	2257(1)	24(1)
N(2)	9750(3)	2487(1)	3235(1)	26(1)
C(2T)	8492(3)	2297(1)	4403(1)	21(1)

Table 3. Bond lengths [Å] and angles [°] for 1s.

C(1)-C(6)	1.3898(15)
C(1)-C(2)	1.4116(14)
C(1)-C(14)	1.4647(14)
C(2)-C(3)	1.3894(14)
C(2)-C(7)	1.4474(14)
C(3)-C(4)	1.3872(15)
C(3)-H(3)	0.9500
C(4)-C(5)	1.3862(15)
C(4)-H(4)	0.9500
C(5)-C(6)	1.3868(14)
C(5)-H(5)	0.9500
C(6)-H(6)	0.9500
C(7)-N(5)	1.1477(14)
C(14)-N(4)	1.2716(14)
C(14)-H(14)	0.9500
N(4)-N(3)	1.3925(11)
N(3)-C(1T)	1.3562(14)
N(3)-C(2T)	1.3673(13)
C(1T)-N(1)	1.2994(14)
C(1T)-H(1T)	0.9500
N(1)-N(2)	1.4011(13)
N(2)-C(2T)	1.3050(14)
C(2T)-H(2T)	0.9500
C(6)-C(1)-C(2)	118.41(9)
C(6)-C(1)-C(14)	116.85(9)
C(2)-C(1)-C(14)	124.74(9)
C(3)-C(2)-C(1)	120.52(9)
C(3)-C(2)-C(7)	116.88(9)
C(1)-C(2)-C(7)	122.54(9)
C(4)-C(3)-C(2)	119.84(9)
C(4)-C(3)-H(3)	120.1
C(2)-C(3)-H(3)	120.1
C(5)-C(4)-C(3)	120.16(9)

Table 3. *Cont.*

C(5)-C(4)-H(4)	119.9
C(3)-C(4)-H(4)	119.9
C(4)-C(5)-C(6)	120.10(9)
C(4)-C(5)-H(5)	119.9
C(6)-C(5)-H(5)	119.9
C(5)-C(6)-C(1)	120.93(9)
C(5)-C(6)-H(6)	119.5
C(1)-C(6)-H(6)	119.5
N(5)-C(7)-C(2)	175.19(11)
N(4)-C(14)-C(1)	120.38(9)
N(4)-C(14)-H(14)	119.8
C(1)-C(14)-H(14)	119.8
C(14)-N(4)-N(3)	116.53(8)
C(1T)-N(3)-C(2T)	105.37(8)
C(1T)-N(3)-N(4)	121.02(8)
C(2T)-N(3)-N(4)	133.43(8)
N(1)-C(1T)-N(3)	110.90(9)
N(1)-C(1T)-H(1T)	124.5
N(3)-C(1T)-H(1T)	124.5
C(1T)-N(1)-N(2)	106.54(9)
C(2T)-N(2)-N(1)	107.61(9)
N(2)-C(2T)-N(3)	109.57(9)
N(2)-C(2T)-H(2T)	125.2
N(3)-C(2T)-H(2T)	125.2

Table 4. Anisotropic displacement parameters ($Å^2 \times 10^3$) for 1s. The anisotropic displacement factor exponent takes the form: $-2\pi^2[h^2a^{*2}U^{11} + ... + 2\ h\ k\ a^*\ b^*\ U^{12}]$.

	**U^{11}**	**U^{22}**	**U^{33}**	**U^{23}**	**U^{13}**	**U^{12}**
C(1)	17(1)	16(1)	18(1)	1(1)	4(1)	3(1)
C(2)	19(1)	17(1)	18(1)	0(1)	4(1)	2(1)
C(3)	22(1)	17(1)	23(1)	1(1)	5(1)	−1(1)
C(4)	22(1)	21(1)	22(1)	5(1)	7(1)	1(1)
C(5)	22(1)	24(1)	17(1)	1(1)	6(1)	2(1)
C(6)	20(1)	18(1)	20(1)	−1(1)	4(1)	1(1)
C(7)	26(1)	16(1)	22(1)	0(1)	7(1)	−2(1)
N(5)	40(1)	22(1)	23(1)	−3(1)	9(1)	−7(1)
C(14)	20(1)	15(1)	19(1)	0(1)	3(1)	1(1)
N(4)	20(1)	15(1)	21(1)	2(1)	7(1)	−1(1)
N(3)	19(1)	17(1)	16(1)	1(1)	4(1)	−1(1)
C(1T)	19(1)	25(1)	16(1)	−1(1)	4(1)	−1(1)
N(1)	27(1)	27(1)	19(1)	1(1)	7(1)	−3(1)
N(2)	35(1)	22(1)	23(1)	1(1)	10(1)	−5(1)
C(2T)	27(1)	17(1)	20(1)	0(1)	6(1)	−3(1)

Table 5. Hydrogen coordinates (×10[4]) and isotropic displacement parameters (Å[2] a 10[3]) for 1s.

	**x**	**y**	**z**	**U(eq)**
H(3)	1869	137	7361	25
H(4)	2271	316	10147	25
H(5)	4413	1045	11207	25
H(6)	6181	1597	9496	23
H(14)	6972	1866	6882	22
H(1T)	9987	1428	2458	24
H(2T)	7724	2466	5240	25

Table 6. Torsion angles [°] for 1s.

C(6)-C(1)-C(2)-C(3)	−1.50(15)
C(14)-C(1)-C(2)-C(3)	178.01(9)
C(6)-C(1)-C(2)-C(7)	175.68(9)
C(14)-C(1)-C(2)-C(7)	−4.81(16)
C(1)-C(2)-C(3)-C(4)	−0.02(15)
C(7)-C(2)-C(3)-C(4)	−177.35(10)
C(2)-C(3)-C(4)-C(5)	0.93(15)
C(3)-C(4)-C(5)-C(6)	−0.30(15)
C(4)-C(5)-C(6)-C(1)	−1.27(15)
C(2)-C(1)-C(6)-C(5)	2.14(15)
C(14)-C(1)-C(6)-C(5)	−177.40(9)
C(6)-C(1)-C(14)-N(4)	165.86(9)
C(2)-C(1)-C(14)-N(4)	−13.65(16)
C(1)-C(14)-N(4)-N(3)	179.63(8)
C(14)-N(4)-N(3)-C(1T)	169.04(9)
C(14)-N(4)-N(3)-C(2T)	−16.68(16)
C(2T)-N(3)-C(1T)-N(1)	0.76(12)
N(4)-N(3)-C(1T)-N(1)	176.45(8)
N(3)-C(1T)-N(1)-N(2)	−0.70(12)
C(1T)-N(1)-N(2)-C(2T)	0.36(12)
N(1)-N(2)-C(2T)-N(3)	0.10(12)
C(1T)-N(3)-C(2T)-N(2)	−0.51(12)
N(4)-N(3)-C(2T)-N(2)	−175.43(10)

Table 7. Hydrogen bonds for 1s [Å and °].

**D-H...A**	**d(D-H)**	**d(H...A)**	**d(D...A)**	**<(DHA)**
C(2T)-H(2T)...N(1)#1	0.95	2.36	3.3096(16)	177.5
C(14)-H(14)...N(2)#1	0.95	2.42	3.2545(15)	146.1

Symmetry transformations used to generate equivalent atoms: #1 x − 1/2, −y + 1/2, z + 1/2.

3.3. 3-[hydroxy(4H-1,2,4-triazol-4-ylamino)methyl]benzonitrile (2)

3.3.1. Synthesis

Acetonitrilic solution (3 mL) of 3-formylbenzonitrile (44 mg) was added to an acetonitrilic solution (3 mL) of 4-amino-1,2,4-triazole (28 mg). The reaction mixture after complete dissolution was stirred for 2 hours at 50 °C. The title compound crystallised directly from the mother liquor. Upon standing 3 days at the room temperature, the solution deposited colourless crystal blocks. The crystals were filtered off, washed with a small amount of acetonitrile and diethyl ether then dried in the air to afford 3-[hydroxy(4*H*-1,2,4-triazol-4-ylamino)methyl]benzonitrile—(56 mg, 78%), mp 115 °C.

3.3.2. Elemental Analysis

	% C	% H	% N
Calculated	55.81	4.22	32.54
Found	55.77	4.06	32.64

3.3.3. Mass Spectrometry

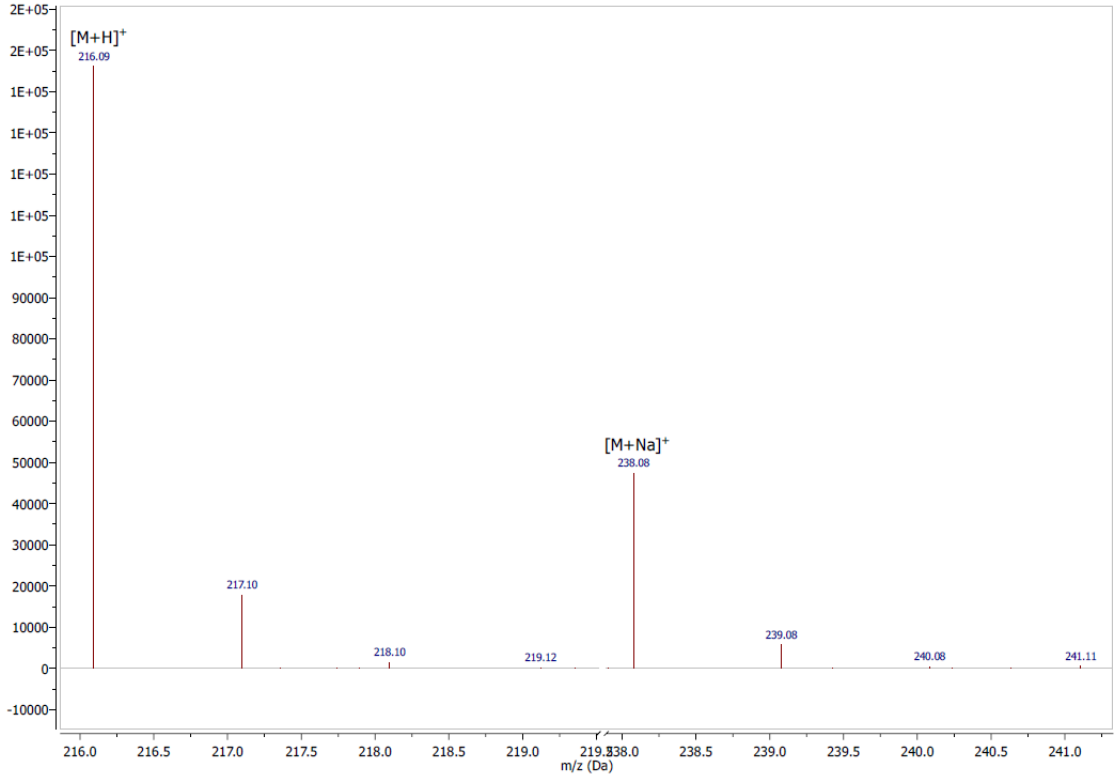

3.3.4. NMR Spectroscopy

^{1}H-NMR

^{1}H-NMR (600 MHz, DMSO, RT): 8.42 (s, 2H, H1T, H2T), 7.88–7.89 (m, 1H, H2), 7.80–7.82 (m, 2H, H4, H6), 7.58–7.61(m, 1H, H5), 7.41 (d, $^{3}J_{H40,H14}$=6.6 Hz, 1H, H40), 6.81 (d, $^{3}J_{H41,H14}$ = 5.5 Hz, 1H, H41), 5.57 (pseudo-triplet, $^{3}J_{H14,H40}$=6.6 Hz, $^{3}J_{H14,H41}$=5.5 Hz, 1H, H14).

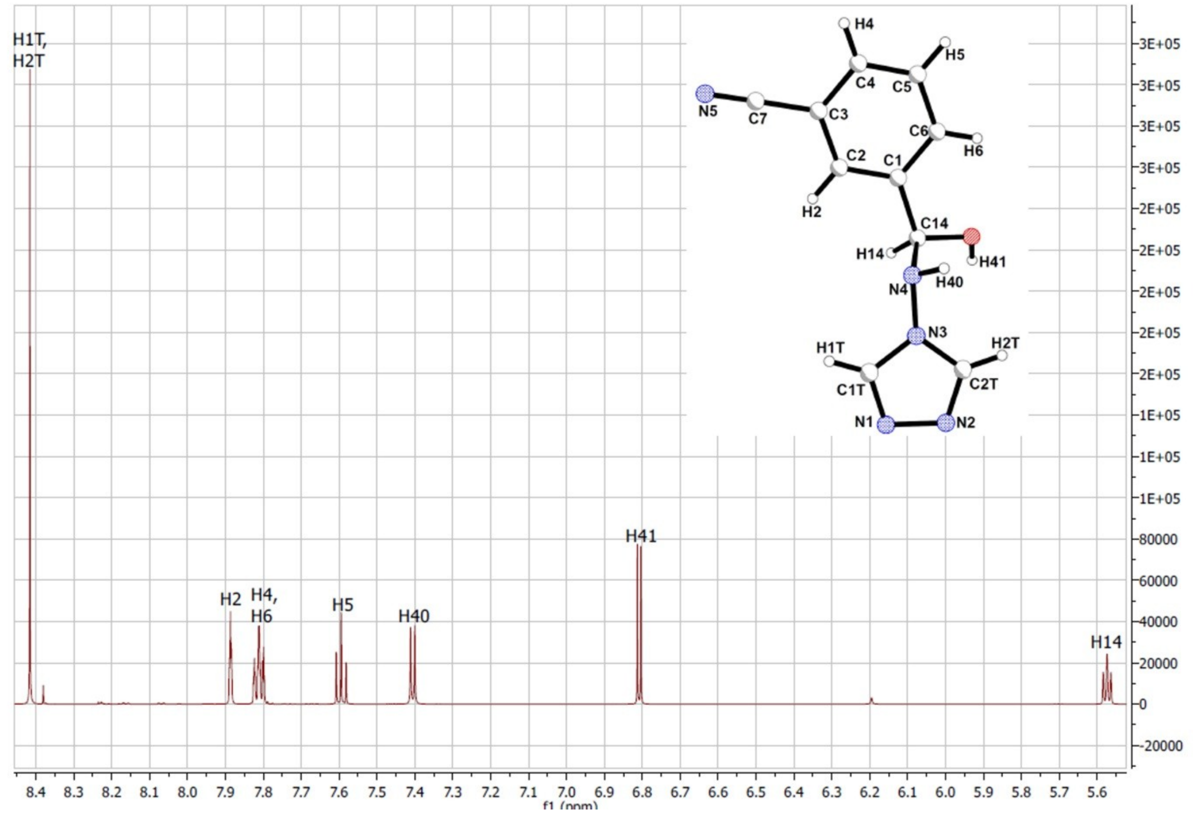

^{13}C-NMR

^{13}C-NMR (150.9 MHz, DMSO, RT): 143.9 (C1T, C2T), 141.5 (C1), 131.9 (C4), 131.5 (C6), 130.3 (C2), 129.4 (C5), 118.7 (C7), 111.0 (C3), 82.6 (C14).

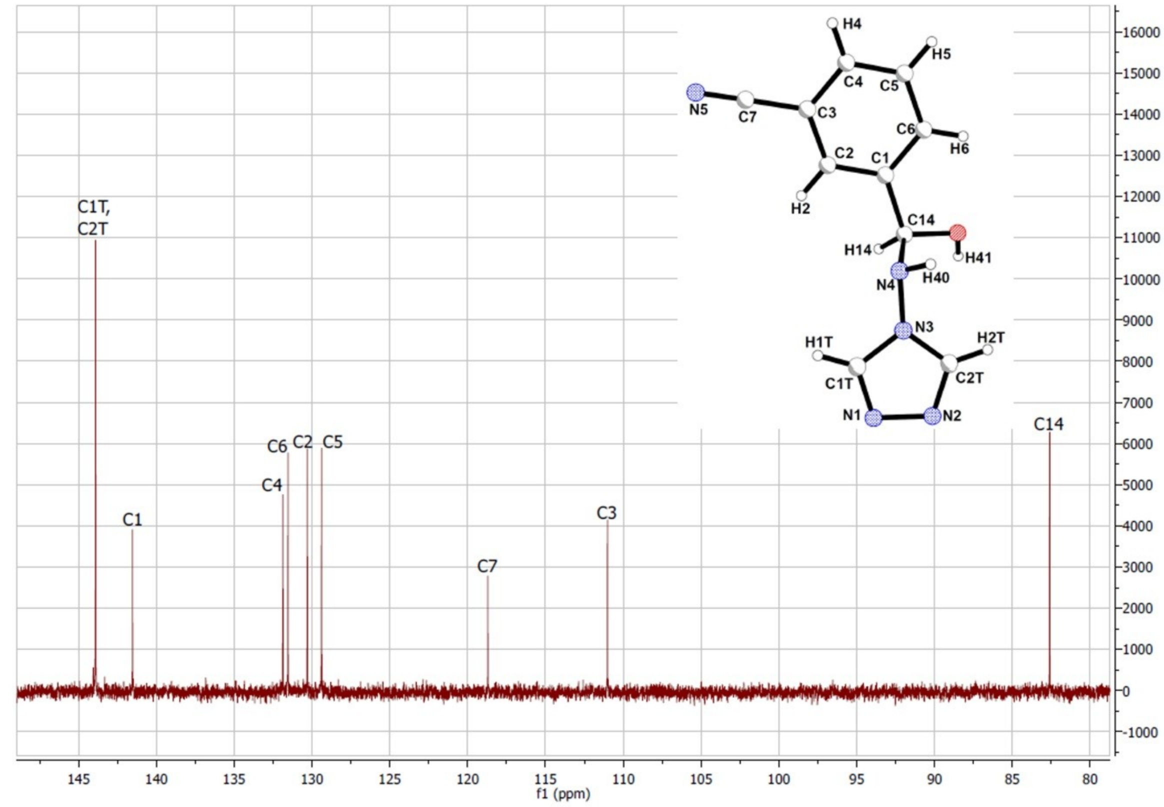

HSQC

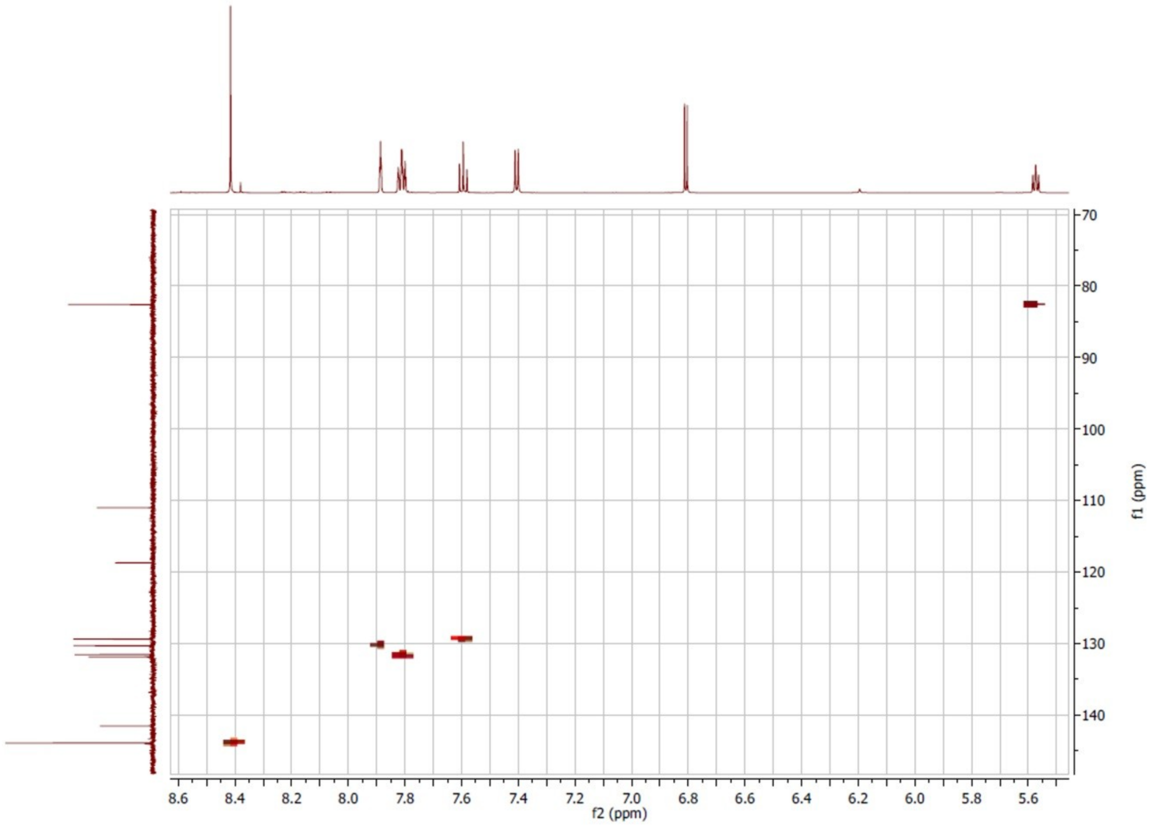
70
80
90
100
110
120
130
140
f1 (ppm)
8.6
8.4
8.2
8.0
7.8
7.6
7.4
7.2
7.0
6.8
6.6
6.4
6.2
6.0
5.8
5.6
f2 (ppm)

HMBC

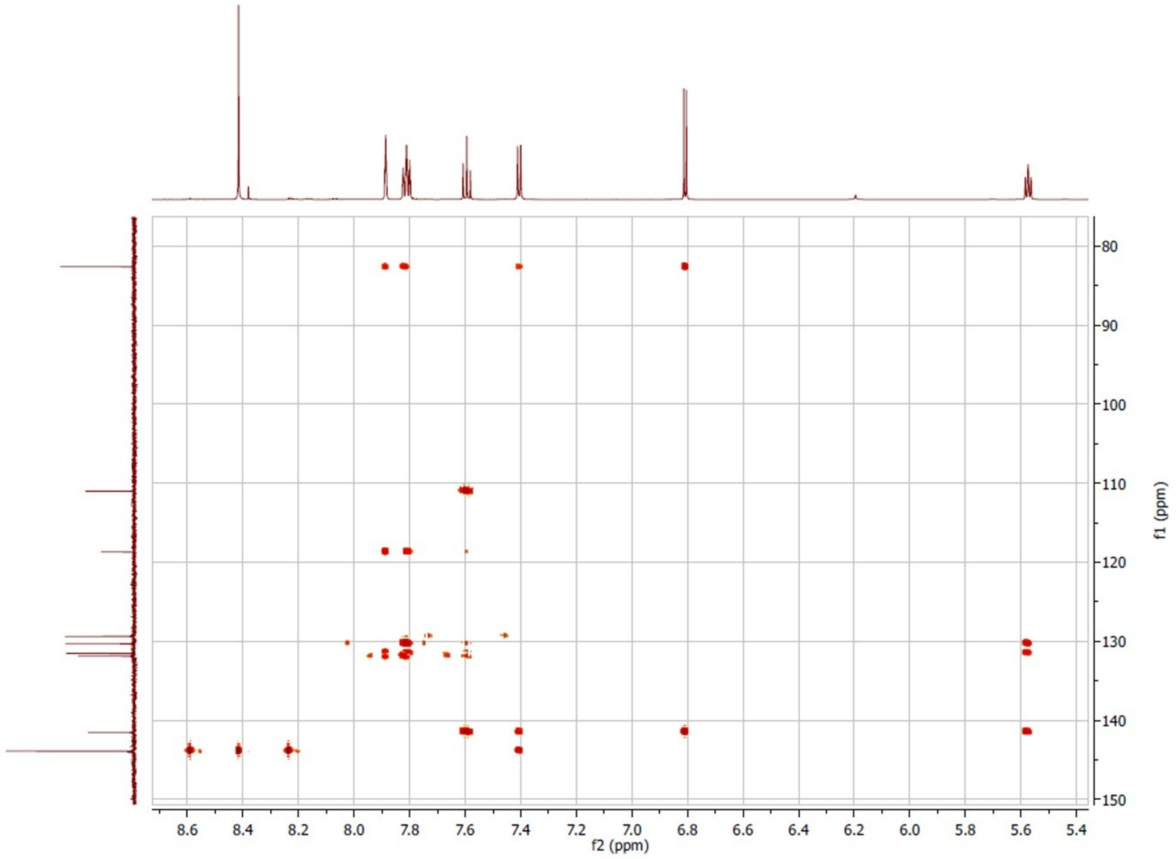
80
90
100
110
120
130
140
150
f1 (ppm)
8.6
8.4
8.2
8.0
7.8
7.6
7.4
7.2
7.0
6.8
6.6
6.4
6.2
6.0
5.8
5.6
5.4
f2 (ppm)

3.3.5. IR Spectroscopy

IR (KBr, cm^{-1}): 3190s, 3118s, 2995m, 2945m, 2870m, 2737m, 2228s, 1961vw, 1903vw, 1734vw, 1718vw, 1701vw, 1685vw, 1653vw, 1636vw, 1607vw, 1586w, 1557m, 1505m, 1483m, 1427m, 1368w, 1340w, 1316m, 1303m, 1276m, 1232vw, 1198m, 1169w, 1148s, 1107m, 1075vs, 1061vs, 1001vw, 987m, 954m, 929s, 924s, 888m, 872s, 814m, 779vs, 767s, 708s, 684s, 645vs, 601w, 577m, 554w, 480m, 463vw, 409vw, 384vw.

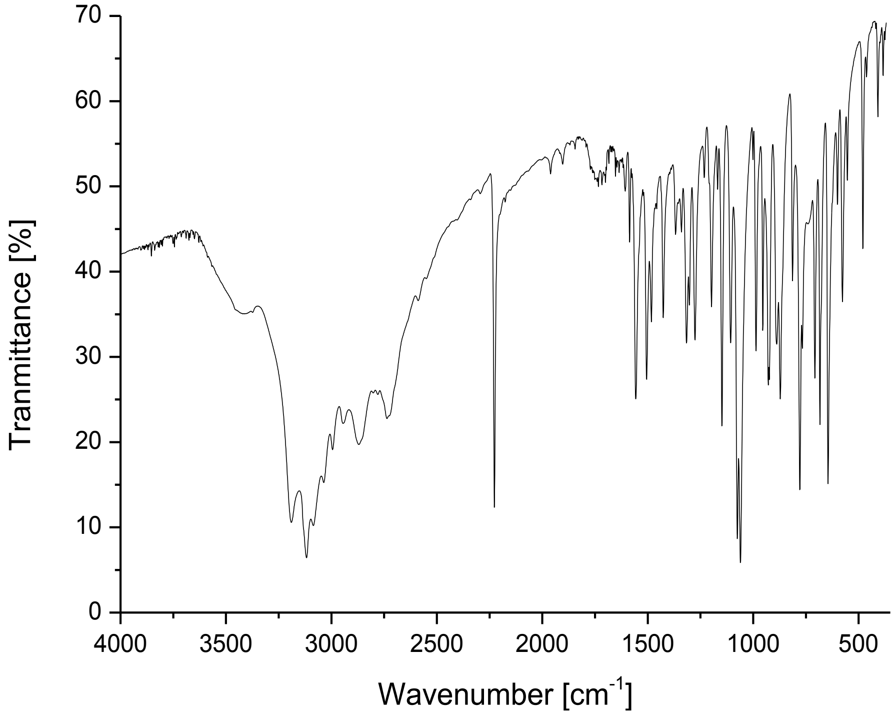

3.3.6. Crystallography

Figure 4. Molecular structure and labelling for 3-[hydroxy(4*H*-1,2,4-triazol-4-ylamino)methyl]benzonitrile (2). Displacement ellipsoids are shown at the 50% probability level.

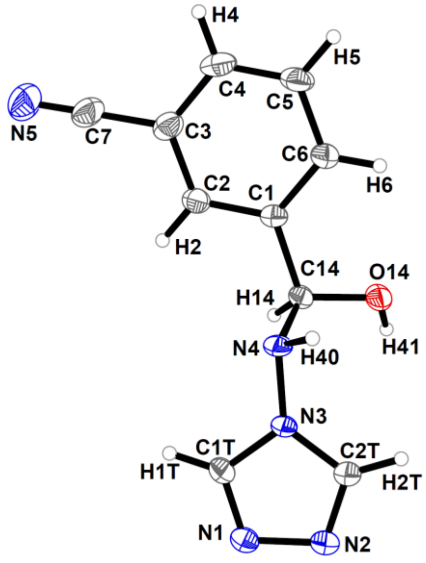

Table 1. Crystal data and structure refinement for 2.

Identification code	2
Empirical formula	C10 H9 N5 O
Formula weight	215.22
Temperature	100(2) K
Wavelength	0.71073 Å
Crystal system	Monoclinic
Space group	P 1 21/c 1
Unit cell dimensions	a = 12.037(4) Å α = 90°.
	b = 10.265(4) Å β = 91.58(3)°.
	c = 8.448(3) Å γ = 90°.
Volume	1043.4(7) Å^3
Z	4
Density (calculated)	1.370 Mg/m^3
Absorption coefficient	0.096 mm^{-1}
F(000)	448
Crystal size	0.27 × 0.26 × 0.18 mm^3
Theta range for data collection	3.12 to 29.99°.
Index ranges	−16 <= h <= 16, −14 <= k <= 14, −9 <= l <= 11
Reflections collected	12783
Independent reflections	2946 [R(int) = 0.0475]
Completeness to theta = 27.00°	99.9%
Absorption correction	Semi-empirical from equivalents
Max. and min. transmission	1.00000 and 0.91099
Refinement method	Full-matrix least-squares on F^2
Data/restraints/parameters	2946/0/151
Goodness-of-fit on F^2	0.987
Final R indices [I > 2sigma(I)]	R1 = 0.0433, wR2 = 0.0924
R indices (all data)	R1 = 0.0771, wR2 = 0.0986
Largest diff. peak and hole	0.273 and −0.206 e.Å^{-3}

Table 2. Atomic coordinates (×10^4) and equivalent isotropic displacement parameters (Å^2 × 10^3) for 2. U(eq) is defined as one third of the trace of the orthogonalized U^{ij} tensor.

	x	y	z	U(eq)
C(1)	7327(1)	6759(1)	6564(1)	21(1)
C(2)	6387(1)	6781(1)	7474(2)	24(1)
C(3)	5880(1)	7963(1)	7829(2)	28(1)
C(4)	6324(1)	9133(1)	7289(2)	31(1)
C(5)	7250(1)	9106(1)	6383(2)	29(1)
C(6)	7752(1)	7925(1)	6011(2)	25(1)
C(7)	4894(1)	7951(1)	8751(2)	35(1)
N(5)	4107(1)	7930(1)	9480(2)	49(1)
C(14)	7864(1)	5455(1)	6221(1)	22(1)
O(14)	8587(1)	5568(1)	4967(1)	27(1)
N(4)	8415(1)	5037(1)	7710(1)	23(1)
N(3)	8720(1)	3706(1)	7649(1)	21(1)
C(1T)	8168(1)	2750(1)	8402(2)	25(1)
N(1)	8640(1)	1629(1)	8160(1)	26(1)
N(2)	9542(1)	1848(1)	7206(1)	26(1)
C(2T)	9565(1)	3098(1)	6916(2)	25(1)

Table 3. Bond lengths [Å] and angles [°] for 2.

C(1)-C(2)	1.3858(18)
C(1)-C(6)	1.3879(17)
C(1)-C(14)	1.5177(17)
C(2)-C(3)	1.3939(18)
C(2)-H(2)	0.9500
C(3)-C(4)	1.396(2)
C(3)-C(7)	1.437(2)
C(4)-C(5)	1.369(2)
C(4)-H(4)	0.9500
C(5)-C(6)	1.3945(18)
C(5)-H(5)	0.9500
C(6)-H(6)	0.9500
C(7)-N(5)	1.1444(19)
C(14)-O(14)	1.3942(15)
C(14)-N(4)	1.4697(17)
C(14)-H(14)	1.0000
O(14)-H(41)	0.917(17)
N(4)-N(3)	1.4158(14)
N(4)-H(40)	0.885(15)
N(3)-C(1T)	1.3530(16)
N(3)-C(2T)	1.3575(16)
C(1T)-N(1)	1.3019(16)
C(1T)-H(1T)	0.9500
N(1)-N(2)	1.3882(15)
N(2)-C(2T)	1.3072(16)
C(2T)-H(2T)	0.9500
C(2)-C(1)-C(6)	119.12(11)
C(2)-C(1)-C(14)	118.62(10)
C(6)-C(1)-C(14)	122.26(11)
C(1)-C(2)-C(3)	120.20(12)
C(1)-C(2)-H(2)	119.9
C(3)-C(2)-H(2)	119.9
C(2)-C(3)-C(4)	120.27(12)
C(2)-C(3)-C(7)	118.84(13)
C(4)-C(3)-C(7)	120.89(12)
C(5)-C(4)-C(3)	119.36(12)
C(5)-C(4)-H(4)	120.3
C(3)-C(4)-H(4)	120.3
C(4)-C(5)-C(6)	120.54(12)
C(4)-C(5)-H(5)	119.7
C(6)-C(5)-H(5)	119.7
C(1)-C(6)-C(5)	120.51(12)
C(1)-C(6)-H(6)	119.7
C(5)-C(6)-H(6)	119.7
N(5)-C(7)-C(3)	179.33(18)

Table 3. *Cont.*

O(14)-C(14)-N(4)	113.45(11)
O(14)-C(14)-C(1)	110.47(10)
N(4)-C(14)-C(1)	106.19(10)
O(14)-C(14)-H(14)	108.9
N(4)-C(14)-H(14)	108.9
C(1)-C(14)-H(14)	108.9
C(14)-O(14)-H(41)	109.4(10)
N(3)-N(4)-C(14)	111.22(9)
N(3)-N(4)-H(40)	105.9(9)
C(14)-N(4)-H(40)	109.0(9)
C(1T)-N(3)-C(2T)	105.37(11)
C(1T)-N(3)-N(4)	123.48(11)
C(2T)-N(3)-N(4)	131.15(10)
N(1)-C(1T)-N(3)	110.18(11)
N(1)-C(1T)-H(1T)	124.9
N(3)-C(1T)-H(1T)	124.9
C(1T)-N(1)-N(2)	107.46(10)
C(2T)-N(2)-N(1)	106.75(10)
N(2)-C(2T)-N(3)	110.23(11)
N(2)-C(2T)-H(2T)	124.9
N(3)-C(2T)-H(2T)	124.9

Table 4. Anisotropic displacement parameters ($Å^2 \times 10^3$) for 2. The anisotropic displacement factor exponent takes the form: $-2\pi^2[\ h^2a^{*2}U^{11} + ... + 2\ h\ k\ a^*\ b^*\ U^{12}]$.

	U^{11}	U^{22}	U^{33}	U^{23}	U^{13}	U^{12}
C(1)	24(1)	16(1)	23(1)	0(1)	0(1)	1(1)
C(2)	24(1)	20(1)	28(1)	3(1)	1(1)	1(1)
C(3)	26(1)	29(1)	28(1)	0(1)	0(1)	8(1)
C(4)	38(1)	21(1)	33(1)	−2(1)	−2(1)	10(1)
C(5)	40(1)	16(1)	32(1)	0(1)	1(1)	0(1)
C(6)	29(1)	18(1)	27(1)	0(1)	4(1)	0(1)
C(7)	31(1)	35(1)	38(1)	3(1)	3(1)	13(1)
N(5)	38(1)	56(1)	54(1)	10(1)	14(1)	18(1)
C(14)	24(1)	14(1)	26(1)	0(1)	5(1)	−3(1)
O(14)	36(1)	17(1)	29(1)	−3(1)	11(1)	−3(1)
N(4)	26(1)	11(1)	31(1)	0(1)	3(1)	1(1)
N(3)	24(1)	12(1)	28(1)	2(1)	5(1)	1(1)
C(1T)	28(1)	18(1)	30(1)	4(1)	7(1)	−2(1)
N(1)	29(1)	17(1)	32(1)	3(1)	5(1)	−1(1)
N(2)	29(1)	19(1)	31(1)	4(1)	4(1)	3(1)
C(2T)	25(1)	19(1)	31(1)	4(1)	5(1)	3(1)

Table 5. Hydrogen coordinates (×10^4) and isotropic displacement parameters (Å^2 × 10^3) for 2.

	x	y	z	U(eq)
H(2)	6087	5989	7857	29
H(4)	5987	9939	7547	37
H(5)	7552	9899	6006	35
H(6)	8390	7919	5374	30
H(14)	7269	4812	5926	26
H(41)	8524(14)	4845(17)	4335(19)	40
H(40)	9044(13)	5475(14)	7845(16)	27
H(1T)	7529	2879	9018	30
H(2T)	10097	3521	6283	30

Table 6. Torsion angles [°] for 2.

C(6)-C(1)-C(2)-C(3)	−0.12(19)
C(14)-C(1)-C(2)-C(3)	179.23(12)
C(1)-C(2)-C(3)-C(4)	−0.9(2)
C(1)-C(2)-C(3)-C(7)	178.89(13)
C(2)-C(3)-C(4)-C(5)	1.2(2)
C(7)-C(3)-C(4)-C(5)	−178.58(13)
C(3)-C(4)-C(5)-C(6)	−0.5(2)
C(2)-C(1)-C(6)-C(5)	0.85(19)
C(14)-C(1)-C(6)-C(5)	−178.48(12)
C(4)-C(5)-C(6)-C(1)	−0.5(2)
C(2)-C(1)-C(14)-O(14)	163.66(11)
C(6)-C(1)-C(14)-O(14)	−17.01(17)
C(2)-C(1)-C(14)-N(4)	−72.94(14)
C(6)-C(1)-C(14)-N(4)	106.39(13)
O(14)-C(14)-N(4)-N(3)	−72.38(12)
C(1)-C(14)-N(4)-N(3)	166.11(9)
C(14)-N(4)-N(3)-C(1T)	−104.80(13)
C(14)-N(4)-N(3)-C(2T)	76.14(16)
C(2T)-N(3)-C(1T)-N(1)	0.27(15)
N(4)-N(3)-C(1T)-N(1)	−179.00(11)
N(3)-C(1T)-N(1)-N(2)	−0.04(15)
C(1T)-N(1)-N(2)-C(2T)	−0.22(14)
N(1)-N(2)-C(2T)-N(3)	0.39(14)
C(1T)-N(3)-C(2T)-N(2)	−0.41(15)
N(4)-N(3)-C(2T)-N(2)	178.77(12)

Table 7. Hydrogen bonds for 2 [Å and °].

D-H...A	d(D-H)	d(H...A)	d(D...A)	<(DHA)
O(14)-H(41)...N(1)#1	0.917(17)	1.817(18)	2.7254(15)	170.5(16)
N(4)-H(40)...N(2)#2	0.885(15)	2.211(16)	3.0818(18)	167.8(13)
C(2T)-H(2T)...O(14)#3	0.95	2.14	3.0902(18)	174.5
C(14)-H(14)...N(5)#4	1.00	2.56	3.553(2)	170.6
C(1T)-H(1T)...N(5)#5	0.95	2.51	3.385(2)	152.7

Symmetry transformations used to generate equivalent atoms: #1 x, −y + 1/2, z − 1/2; #2 −x + 2, y + 1/2, −z + 3/2; #3 −x + 2, −y + 1, −z + 1; #4 −x + 1, y − 1/2, −z + 3/2; #5 −x + 1, −y + 1, −z + 2.

3.4. 3-[(E)-(4H-1,2,4-triazol-4-ylimino)methyl]benzonitrile (2s)

3.4.1. Synthesis

Ethanolic solution (3 mL) of 3-formylbenzonitrile (49 mg) was added to an ethanolic solution (3 mL) of 4-amino-1,2,4-triazole (31 mg). Few drops of hydrochloric acid were added to the obtained solution. The reaction mixture after complete dissolution was refluxed for 4 hours. The title compound crystallised directly from the mother liquor. Upon standing 3 days at the room temperature, the solution deposited colourless crystal needles. The crystals were filtered off, washed with a small amount of ethanol and diethyl ether then dried in the air to afford 3-[(*E*)-(4*H*-1,2,4-triazol-4-ylimino)methyl]benzonitrile—(49 mg, 67%), mp 237 °C.

3.4.2. Elemental Analysis

	**% C**	**% H**	**% N**
Calculated	60.91	3.58	35.51
Found	60.95	3.69	35.70

3.4.3. Mass Spectrometry

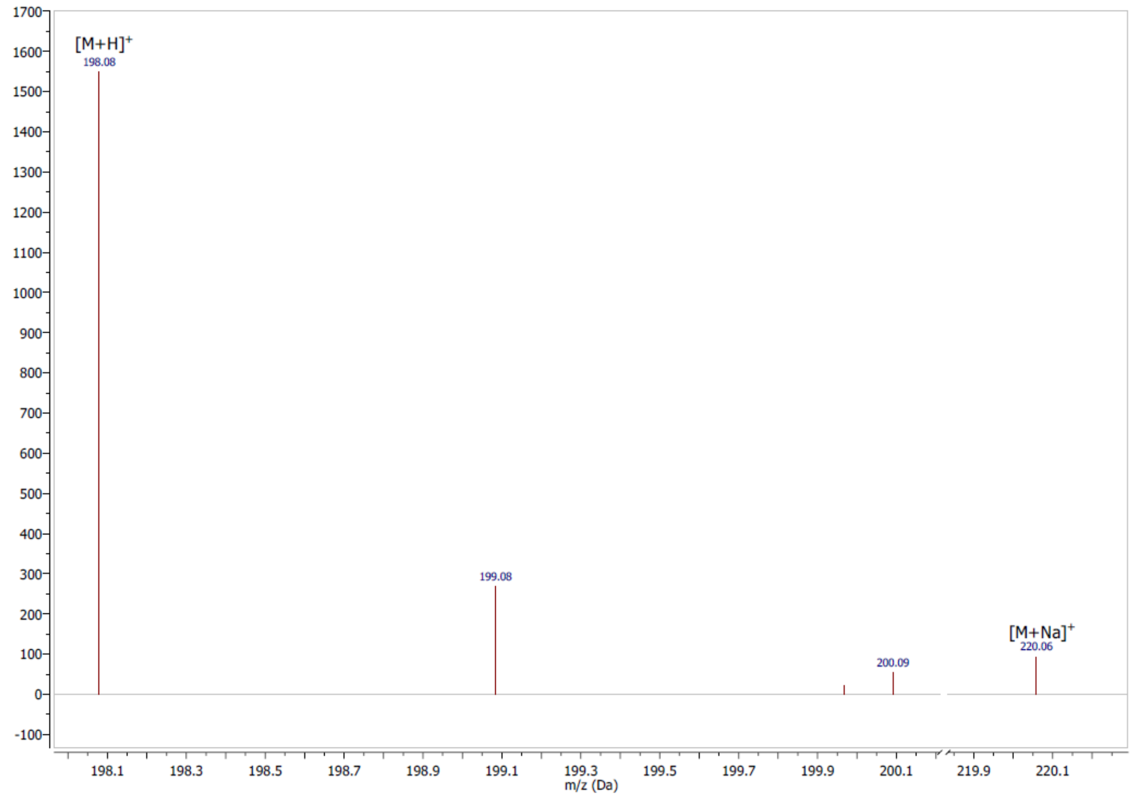

3.4.4. NMR Spectroscopy

^{1}H-NMR

^{1}H-NMR (600 MHz, DMSO, RT): 9.16 (s, 2H, H1T, H2T), 9.15 (s, 1H, H14), 8.22–8.23 (m, 1H, H2), 8.15–8.17 (m, 1H, H6), 8.06–8.08 (m, 1H, H4), 7.77–7.80 (m, 1H, H5).

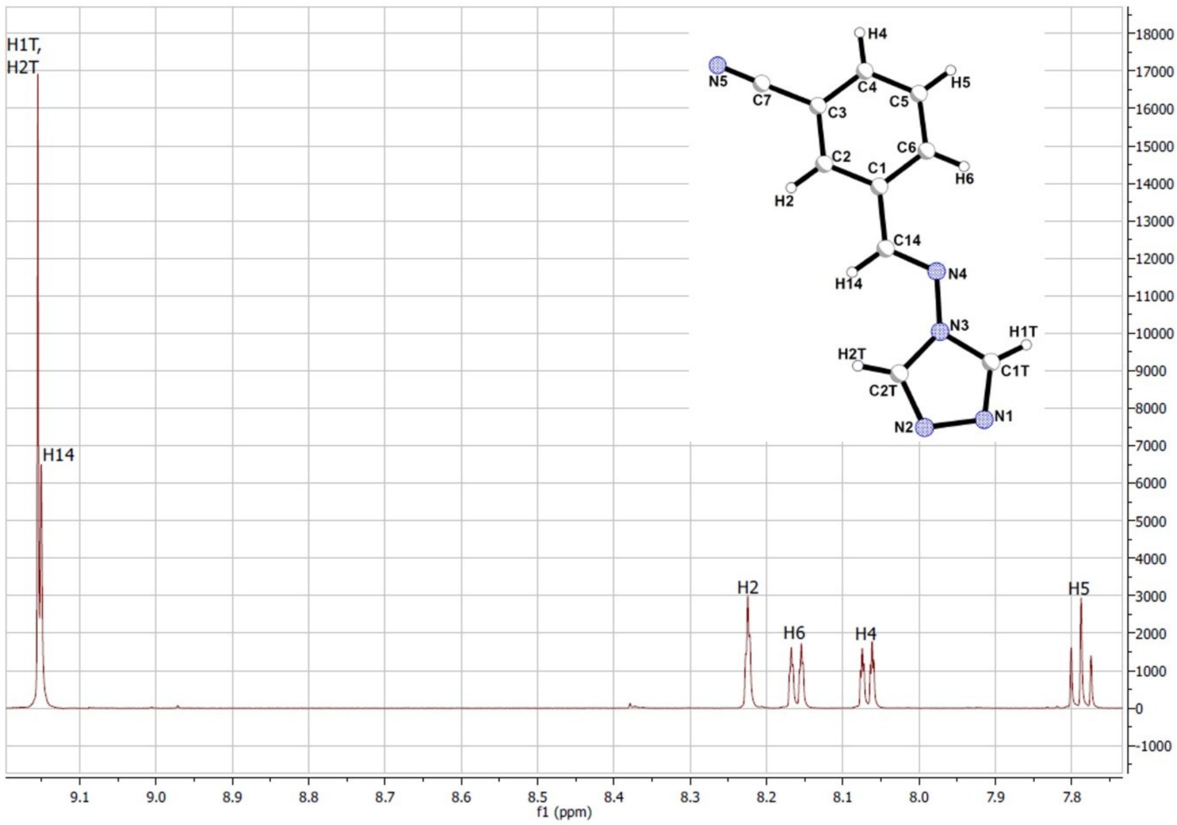

^{13}C-NMR

^{13}C-NMR (150.9 MHz, DMSO, RT): 156.2 (C14), 139.0 (C1T, C2T), 135.4 (C4), 133.4 (C1), 132.5 (C6), 131.7 (C2), 130.5 (C5), 118.0 (C7), 112.4 (C3).

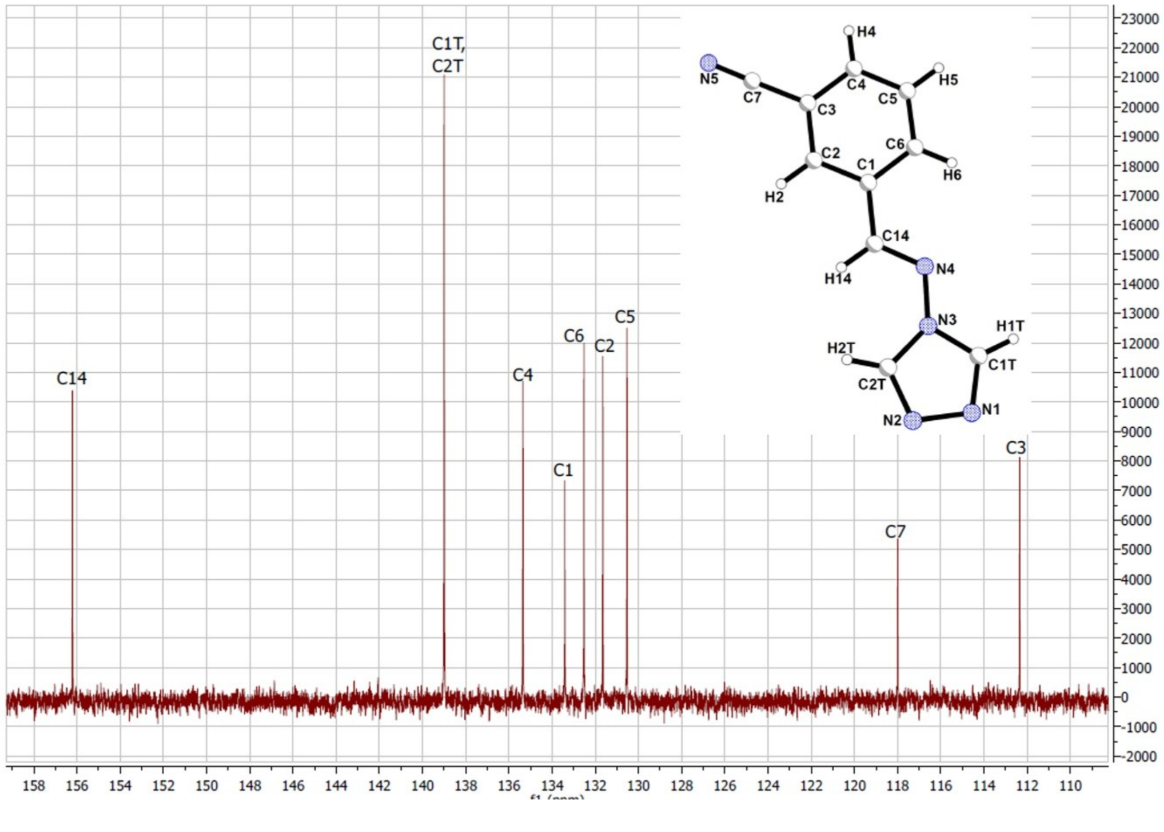

HMQC

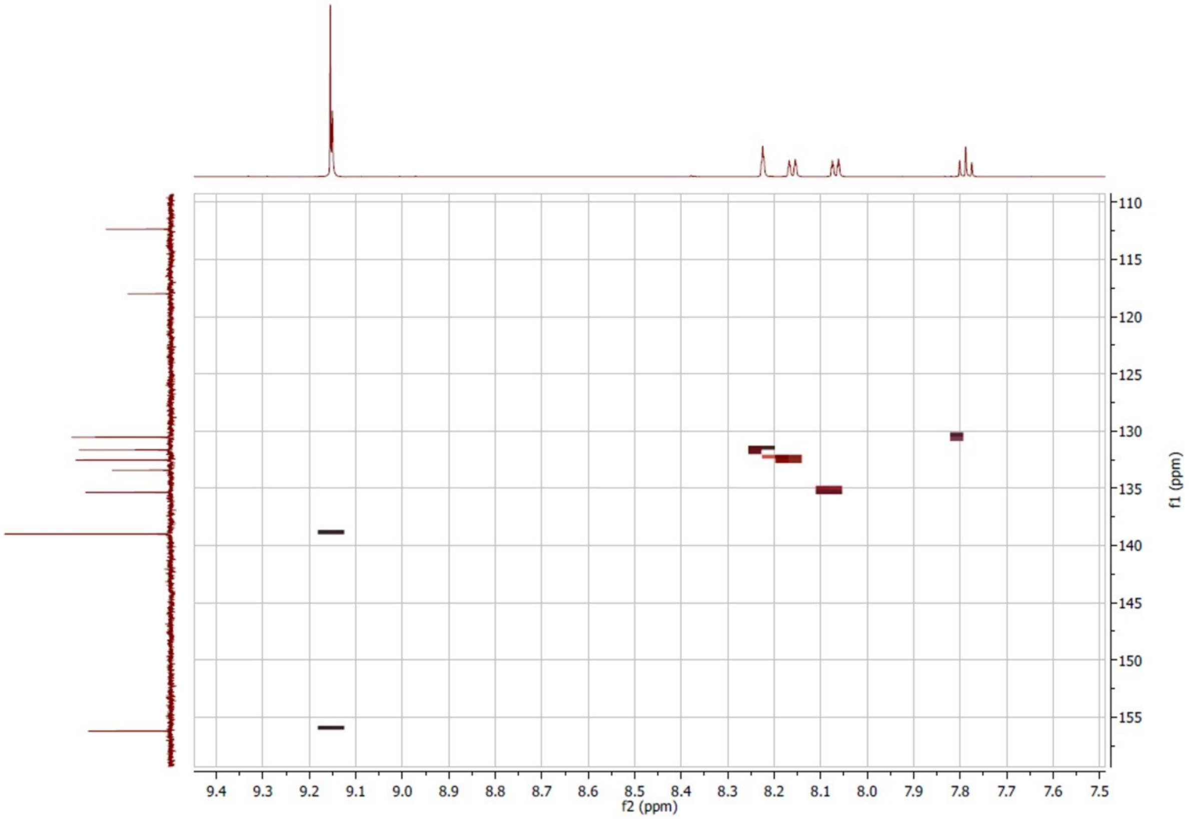
9.4
9.3
9.2
9.1
9.0
8.9
8.8
8.7
8.6
8.5
8.4
8.3
8.2
8.1
8.0
7.9
7.8
7.7
7.6
7.5
f2 (ppm)
110
115
120
125
130
135
140
145
150
155
f1 (ppm)

HMBC

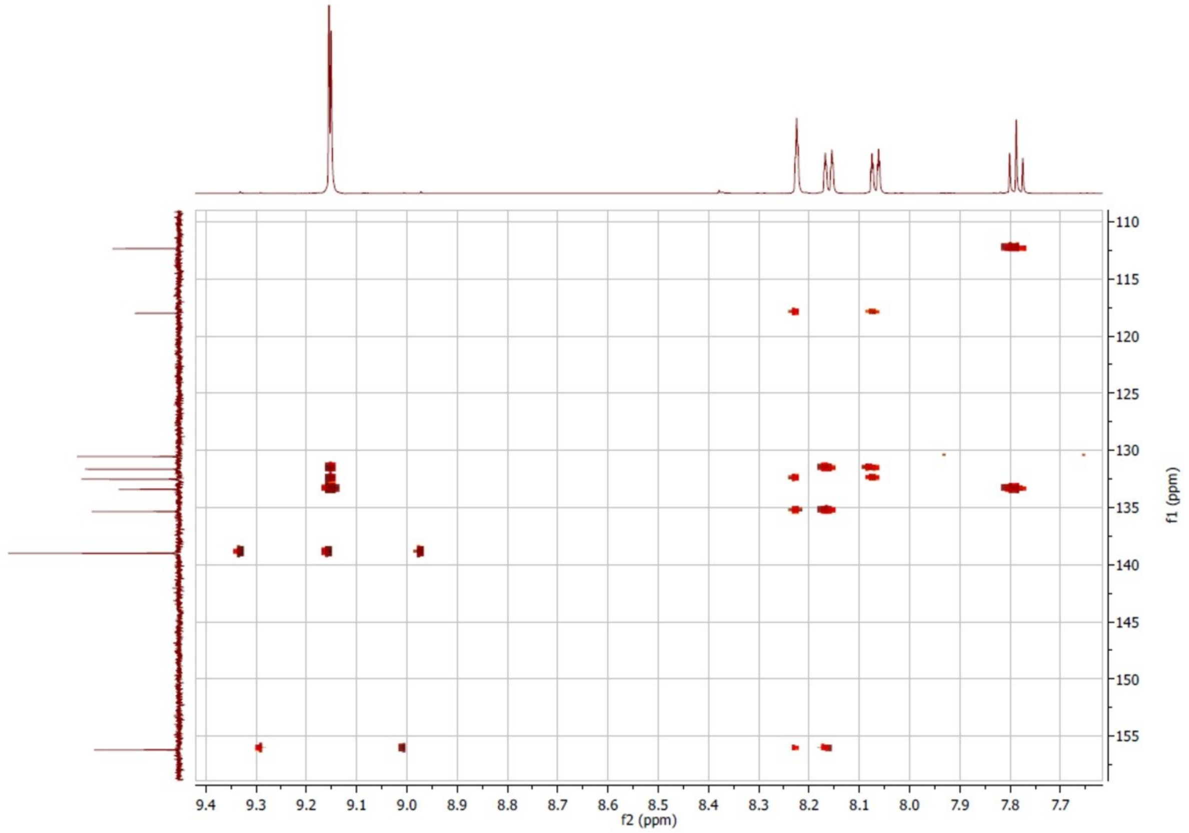
9.4
9.3
9.2
9.1
9.0
8.9
8.8
8.7
8.6
8.5
8.4
8.3
8.2
8.1
8.0
7.9
7.8
7.7
f2 (ppm)
110
115
120
125
130
135
140
145
150
155
f1 (ppm)

3.4.5. IR Spectroscopy

IR (KBr, cm^{-1}): 3443w, 3130m, 3079m, 3062m, 3034w, 2956w, 2231s, 1722vw, 1614w, 1578vw, 1529w, 1505vs, 1487m, 1471w, 1430vw, 1391w, 1345vw, 1329w, 1308w, 1286m, 1250vw, 1226w, 1177vs, 1170vs, 1096vw, 1057vs, 1002vw, 977s, 963w, 942m, 929w, 910vw, 871m, 819vw, 797s, 712m, 685vs, 627m, 606w, 596m, 497m, 475w, 457vw.

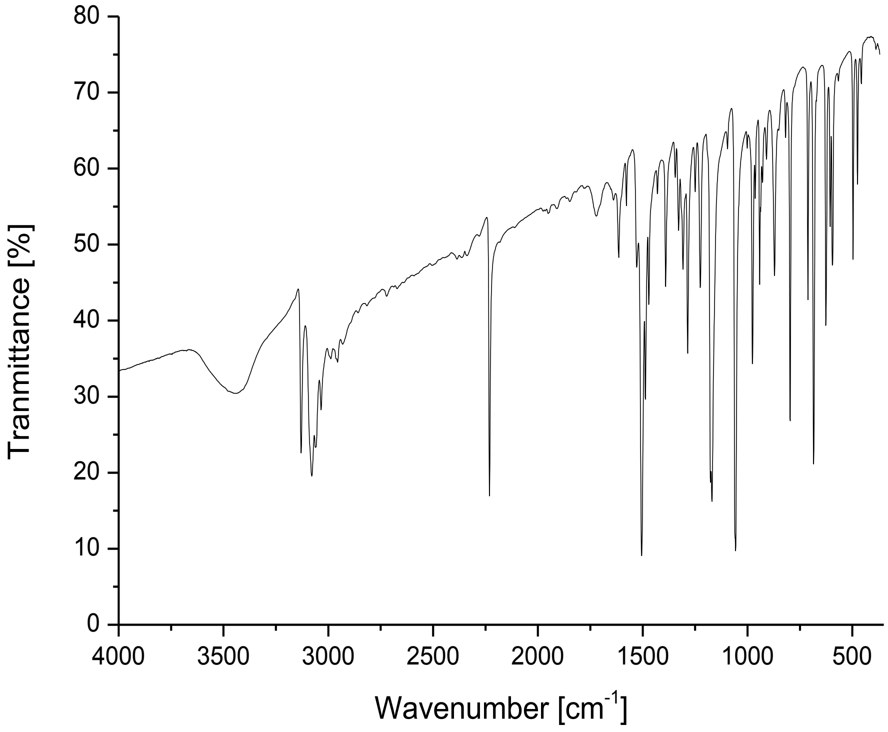

3.4.6. Crystallography

Figure 5. Molecular structure and labelling for 3-[(*E*)-(4*H*-1,2,4-triazol-4-ylimino)methyl]benzonitrile (2s). Displacement ellipsoids are shown at the 50% probability level.

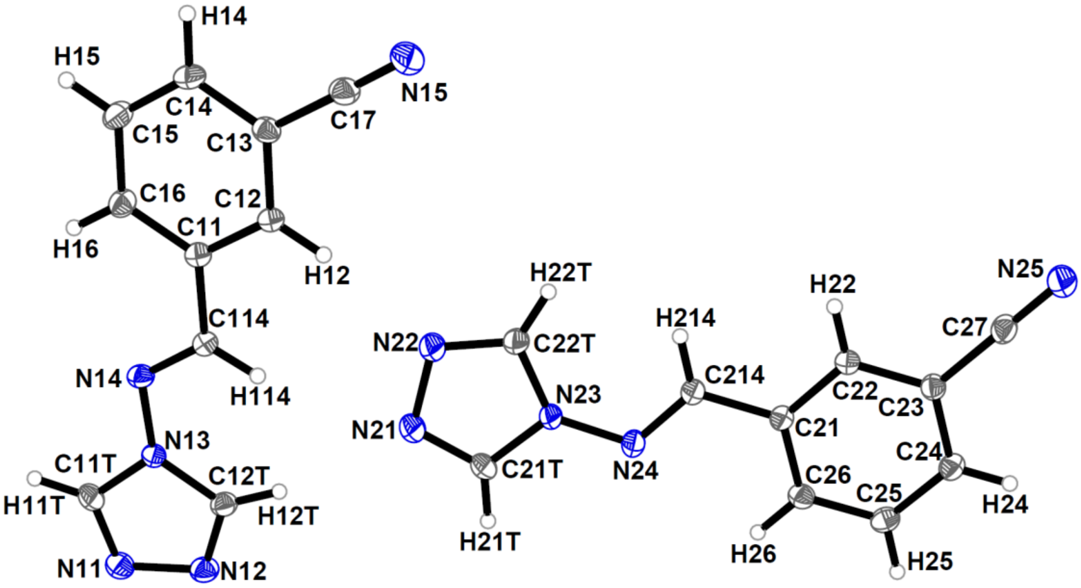

Table 1. Crystal data and structure refinement for 2s.

Identification code	2s	
Empirical formula	C10 H7 N5	
Formula weight	197.21	
Temperature	100(2) K	
Wavelength	0.71073 Å	
Crystal system	Triclinic	
Space group	P -1	
Unit cell dimensions	a = 3.811(2) Å	α = 95.60(3)°
	b = 10.734(3) Å	β = 90.48(3)°
	c = 23.299(5) Å	γ = 98.63(3)°
Volume	937.6(6) $Å^3$	
Z	4	
Density (calculated)	1.397 Mg/m^3	
Absorption coefficient	0.093 mm^{-1}	
F(000)	408	
Crystal size	0.53 × 0.18 × 0.07 mm^3	
Theta range for data collection	3.11 to 36.95°.	
Index ranges	−6 <= h <= 3, −17 <= k <= 14, −39 <= l <= 31	
Reflections collected	13312	
Independent reflections	6533 [R(int) = 0.0267]	
Completeness to theta = 27.00°	99.8%	
Absorption correction	Semi-empirical from equivalents	
Max. and min. transmission	1.00000 and 0.98567	
Refinement method	Full-matrix least-squares on F^2	
Data/restraints/parameters	6533/0/271	
Goodness-of-fit on F^2	0.975	
Final R indices [I > 2sigma(I)]	R1 = 0.0513, wR2 = 0.1510	
R indices (all data)	R1 = 0.0806, wR2 = 0.1613	
Largest diff. peak and hole	0.515 and −0.269 e.$Å^{-3}$	

Table 2. Atomic coordinates ($\times 10^4$) and equivalent isotropic displacement parameters ($Å^2 \times 10^3$) for 2s. U(eq) is defined as one third of the trace of the orthogonalized U^{ij} tensor.

C(11)	6814(3)	1387(1)	3946(1)	15(1)
C(12)	8351(3)	2588(1)	3826(1)	15(1)
C(13)	10400(4)	3382(1)	4256(1)	17(1)
C(14)	10849(4)	2998(1)	4803(1)	19(1)
C(15)	9292(4)	1798(1)	4917(1)	21(1)
C(16)	7339(4)	992(1)	4491(1)	19(1)
C(17)	12113(4)	4611(1)	4124(1)	20(1)
N(15)	13536(4)	5584(1)	4025(1)	27(1)
C(114)	4646(4)	578(1)	3492(1)	16(1)
N(14)	3286(3)	−551(1)	3585(1)	17(1)
N(13)	1164(3)	−1248(1)	3142(1)	16(1)
C(11T)	−220(4)	−2484(1)	3167(1)	20(1)
N(11)	−2204(3)	−2917(1)	2709(1)	22(1)

Table 2. *Cont.*

N(12)	−2153(3)	−1913(1)	2369(1)	21(1)
C(12T)	−127(4)	−934(1)	2635(1)	18(1)
C(21)	1025(4)	6488(1)	1107(1)	15(1)
C(22)	2493(4)	7759(1)	1174(1)	16(1)
C(23)	2002(4)	8520(1)	737(1)	17(1)
C(24)	69(4)	8019(1)	232(1)	19(1)
C(25)	−1397(4)	6750(1)	173(1)	19(1)
C(26)	−947(4)	5984(1)	606(1)	18(1)
C(27)	3604(4)	9825(1)	801(1)	20(1)
N(25)	4908(4)	10872(1)	853(1)	27(1)
C(214)	1739(4)	5699(1)	1562(1)	17(1)
N(24)	604(3)	4511(1)	1496(1)	19(1)
N(23)	1465(3)	3810(1)	1932(1)	18(1)
C(21T)	488(4)	2534(1)	1901(1)	21(1)
N(21)	1642(4)	2090(1)	2355(1)	23(1)
N(22)	3452(3)	3118(1)	2707(1)	22(1)
C(22T)	3310(4)	4136(1)	2448(1)	19(1)

Table 3. Bond lengths [Å] and angles [°] for 2s.

C(11)-C(12)	1.3890(19)
C(11)-C(16)	1.4013(19)
C(11)-C(114)	1.4643(19)
C(12)-C(13)	1.4009(18)
C(12)-H(12)	0.9500
C(13)-C(14)	1.392(2)
C(13)-C(17)	1.445(2)
C(14)-C(15)	1.386(2)
C(14)-H(14)	0.9500
C(15)-C(16)	1.383(2)
C(15)-H(15)	0.9500
C(16)-H(16)	0.9500
C(17)-N(15)	1.1475(19)
C(114)-N(14)	1.2839(18)
C(114)-H(114)	0.9500
N(14)-N(13)	1.3908(16)
N(13)-C(11T)	1.3588(18)
N(13)-C(12T)	1.3673(18)
C(11T)-N(11)	1.309(2)
C(11T)-H(11T)	0.9500
N(11)-N(12)	1.3954(19)
N(12)-C(12T)	1.3049(18)
C(12T)-H(12T)	0.9500
C(21)-C(22)	1.3876(18)
C(21)-C(26)	1.4003(19)
C(21)-C(214)	1.4689(19)

Table 3. *Cont.*

C(22)-C(23)	1.3946(19)
C(22)-H(22)	0.9500
C(23)-C(24)	1.4009(19)
C(23)-C(27)	1.4351(19)
C(24)-C(25)	1.386(2)
C(24)-H(24)	0.9500
C(25)-C(26)	1.388(2)
C(25)-H(25)	0.9500
C(26)-H(26)	0.9500
C(27)-N(25)	1.1524(19)
C(214)-N(24)	1.2774(18)
C(214)-H(214)	0.9500
N(24)-N(23)	1.3863(17)
N(23)-C(21T)	1.3585(18)
N(23)-C(22T)	1.3754(18)
C(21T)-N(21)	1.301(2)
C(21T)-H(21T)	0.9500
N(21)-N(22)	1.3966(18)
N(22)-C(22T)	1.3066(19)
C(22T)-H(22T)	0.9500
C(12)-C(11)-C(16)	119.67(12)
C(12)-C(11)-C(114)	118.18(12)
C(16)-C(11)-C(114)	122.14(12)
C(11)-C(12)-C(13)	118.99(13)
C(11)-C(12)-H(12)	120.5
C(13)-C(12)-H(12)	120.5
C(14)-C(13)-C(12)	121.23(12)
C(14)-C(13)-C(17)	119.85(12)
C(12)-C(13)-C(17)	118.92(13)
C(15)-C(14)-C(13)	119.16(12)
C(15)-C(14)-H(14)	120.4
C(13)-C(14)-H(14)	120.4
C(16)-C(15)-C(14)	120.26(13)
C(16)-C(15)-H(15)	119.9
C(14)-C(15)-H(15)	119.9
C(15)-C(16)-C(11)	120.64(13)
C(15)-C(16)-H(16)	119.7
C(11)-C(16)-H(16)	119.7
N(15)-C(17)-C(13)	178.56(17)
N(14)-C(114)-C(11)	119.47(13)
N(14)-C(114)-H(114)	120.3
C(11)-C(114)-H(114)	120.3
C(114)-N(14)-N(13)	116.19(12)
C(11T)-N(13)-C(12T)	105.27(11)
C(11T)-N(13)-N(14)	122.09(12)

Table 3. *Cont.*

C(12T)-N(13)-N(14)	132.58(12)
N(11)-C(11T)-N(13)	110.53(13)
N(11)-C(11T)-H(11T)	124.7
N(13)-C(11T)-H(11T)	124.7
C(11T)-N(11)-N(12)	106.68(11)
C(12T)-N(12)-N(11)	107.64(12)
N(12)-C(12T)-N(13)	109.87(13)
N(12)-C(12T)-H(12T)	125.1
N(13)-C(12T)-H(12T)	125.1
C(22)-C(21)-C(26)	120.04(13)
C(22)-C(21)-C(214)	117.92(12)
C(26)-C(21)-C(214)	122.00(12)
C(21)-C(22)-C(23)	119.32(12)
C(21)-C(22)-H(22)	120.3
C(23)-C(22)-H(22)	120.3
C(22)-C(23)-C(24)	120.98(12)
C(22)-C(23)-C(27)	119.20(12)
C(24)-C(23)-C(27)	119.79(13)
C(25)-C(24)-C(23)	118.95(13)
C(25)-C(24)-H(24)	120.5
C(23)-C(24)-H(24)	120.5
C(24)-C(25)-C(26)	120.66(13)
C(24)-C(25)-H(25)	119.7
C(26)-C(25)-H(25)	119.7
C(25)-C(26)-C(21)	120.05(12)
C(25)-C(26)-H(26)	120.0
C(21)-C(26)-H(26)	120.0
N(25)-C(27)-C(23)	179.63(18)
N(24)-C(214)-C(21)	119.32(12)
N(24)-C(214)-H(214)	120.3
C(21)-C(214)-H(214)	120.3
C(214)-N(24)-N(23)	116.66(12)
C(21T)-N(23)-C(22T)	105.38(12)
C(21T)-N(23)-N(24)	121.91(12)
C(22T)-N(23)-N(24)	132.71(11)
N(21)-C(21T)-N(23)	110.51(12)
N(21)-C(21T)-H(21T)	124.7
N(23)-C(21T)-H(21T)	124.7
C(21T)-N(21)-N(22)	107.11(12)
C(22T)-N(22)-N(21)	107.59(12)
N(22)-C(22T)-N(23)	109.41(12)
N(22)-C(22T)-H(22T)	125.3
N(23)-C(22T)-H(22T)	125.3

Table 4. Anisotropic displacement parameters (Å^2 × 10^3) for 2s. The anisotropic displacement factor exponent takes the form: $-2\pi^2$[$h^2a^{*2}U^{11}$ + ... + 2 h k a* b* U^{12}].

	U^{11}	U^{22}	U^{33}	U^{23}	U^{13}	U^{12}
C(11)	14(1)	16(1)	15(1)	1(1)	1(1)	2(1)
C(12)	16(1)	16(1)	14(1)	0(1)	−1(1)	2(1)
C(13)	15(1)	16(1)	20(1)	−1(1)	−2(1)	2(1)
C(14)	18(1)	22(1)	17(1)	−2(1)	−4(1)	1(1)
C(15)	23(1)	24(1)	16(1)	3(1)	−3(1)	2(1)
C(16)	19(1)	21(1)	16(1)	4(1)	0(1)	1(1)
C(17)	20(1)	19(1)	21(1)	−1(1)	−4(1)	3(1)
N(15)	30(1)	21(1)	30(1)	2(1)	−6(1)	−2(1)
C(114)	17(1)	17(1)	14(1)	2(1)	0(1)	3(1)
N(14)	18(1)	17(1)	16(1)	0(1)	−2(1)	0(1)
N(13)	18(1)	14(1)	16(1)	1(1)	−3(1)	0(1)
C(11T)	24(1)	13(1)	24(1)	2(1)	−1(1)	−1(1)
N(11)	25(1)	17(1)	23(1)	0(1)	−2(1)	−2(1)
N(12)	22(1)	20(1)	19(1)	0(1)	−3(1)	−1(1)
C(12T)	19(1)	18(1)	16(1)	1(1)	−1(1)	1(1)
C(21)	15(1)	16(1)	15(1)	3(1)	0(1)	2(1)
C(22)	17(1)	15(1)	15(1)	1(1)	−1(1)	2(1)
C(23)	17(1)	16(1)	17(1)	3(1)	0(1)	2(1)
C(24)	19(1)	20(1)	16(1)	3(1)	−1(1)	3(1)
C(25)	18(1)	23(1)	17(1)	0(1)	−4(1)	2(1)
C(26)	18(1)	17(1)	18(1)	0(1)	−1(1)	1(1)
C(27)	20(1)	21(1)	18(1)	5(1)	−3(1)	2(1)
N(25)	32(1)	23(1)	25(1)	5(1)	−5(1)	−2(1)
C(214)	18(1)	17(1)	15(1)	3(1)	−1(1)	2(1)
N(24)	24(1)	17(1)	17(1)	5(1)	−3(1)	1(1)
N(23)	22(1)	14(1)	17(1)	3(1)	−3(1)	−1(1)
C(21T)	25(1)	13(1)	22(1)	1(1)	−2(1)	−2(1)
N(21)	28(1)	16(1)	23(1)	3(1)	−2(1)	−1(1)
N(22)	26(1)	18(1)	21(1)	4(1)	−3(1)	0(1)
C(22T)	22(1)	17(1)	17(1)	3(1)	−4(1)	−1(1)

Table 5. Hydrogen coordinates (×10^4) and isotropic displacement parameters (Å^2 a 10^3) for 2s.

	x	y	z	U(eq)
H(12)	8017	2868	3457	18
H(14)	12206	3551	5093	23
H(15)	9566	1529	5289	25
H(16)	6344	162	4570	23
H(114)	4248	885	3132	19
H(11T)	194	−2966	3475	24
H(12T)	376	−124	2496	22
H(22)	3820	8106	1515	19
H(24)	−232	8541	−66	22
H(25)	−2724	6401	−167	23
H(26)	−1978	5116	562	21
H(214)	3032	6069	1903	20
H(21T)	−851	2037	1592	25
H(22T)	4323	4971	2593	23

Table 6. Torsion angles [°] for 2s.

C(16)-C(11)-C(12)-C(13)	−0.1(2)
C(114)-C(11)-C(12)-C(13)	179.26(13)
C(11)-C(12)-C(13)-C(14)	−1.5(2)
C(11)-C(12)-C(13)-C(17)	177.55(14)
C(12)-C(13)-C(14)-C(15)	1.3(2)
C(17)-C(13)-C(14)-C(15)	−177.74(14)
C(13)-C(14)-C(15)-C(16)	0.5(2)
C(14)-C(15)-C(16)-C(11)	−2.0(2)
C(12)-C(11)-C(16)-C(15)	1.8(2)
C(114)-C(11)-C(16)-C(15)	−177.51(14)
C(12)-C(11)-C(114)-N(14)	177.84(14)
C(16)-C(11)-C(114)-N(14)	−2.9(2)
C(11)-C(114)-N(14)-N(13)	177.95(12)
C(114)-N(14)-N(13)-C(11T)	175.06(14)
C(114)-N(14)-N(13)-C(12T)	−8.3(2)
C(12T)-N(13)-C(11T)-N(11)	0.55(17)
N(14)-N(13)-C(11T)-N(11)	178.01(13)
N(13)-C(11T)-N(11)-N(12)	−0.75(17)
C(11T)-N(11)-N(12)-C(12T)	0.67(17)
N(11)-N(12)-C(12T)-N(13)	−0.34(17)
C(11T)-N(13)-C(12T)-N(12)	−0.11(16)
N(14)-N(13)-C(12T)-N(12)	−177.19(14)
C(26)-C(21)-C(22)-C(23)	−0.4(2)
C(214)-C(21)-C(22)-C(23)	177.07(13)
C(21)-C(22)-C(23)-C(24)	−0.4(2)
C(21)-C(22)-C(23)-C(27)	−178.33(13)
C(22)-C(23)-C(24)-C(25)	0.7(2)
C(27)-C(23)-C(24)-C(25)	178.71(14)
C(23)-C(24)-C(25)-C(26)	−0.3(2)
C(24)-C(25)-C(26)-C(21)	−0.4(2)
C(22)-C(21)-C(26)-C(25)	0.8(2)
C(214)-C(21)-C(26)-C(25)	−176.56(14)
C(22)-C(21)-C(214)-N(24)	−175.80(14)
C(26)-C(21)-C(214)-N(24)	1.6(2)
C(21)-C(214)-N(24)-N(23)	177.85(12)
C(214)-N(24)-N(23)-C(21T)	−178.16(14)
C(214)-N(24)-N(23)-C(22T)	1.4(2)
C(22T)-N(23)-C(21T)-N(21)	−0.58(17)
N(24)-N(23)-C(21T)-N(21)	179.08(13)
N(23)-C(21T)-N(21)-N(22)	0.43(18)
C(21T)-N(21)-N(22)-C(22T)	−0.10(17)
N(21)-N(22)-C(22T)-N(23)	−0.26(17)
C(21T)-N(23)-C(22T)-N(22)	0.51(17)
N(24)-N(23)-C(22T)-N(22)	−179.10(15)

Table 7. Hydrogen bonds for 2s [Å and °].

D-H...A	d(D-H)	d(H...A)	d(D...A)	<(DHA)
C(12T)-H(12T)...N(21)	0.95	2.41	3.343(2)	168.0
C(22T)-H(22T)...N(11)#1	0.95	2.44	3.359(2)	163.8
C(21T)-H(21T)...N(25)#2	0.95	2.46	3.405(2)	171.4
C(22)-H(22)...N(12)#1	0.95	2.51	3.397(2)	155.8
C(12)-H(12)...N(22)	0.95	2.52	3.336(2)	144.4
C(11T)-H(11T)...N(15)#2	0.95	2.58	3.463(2)	155.4
C(114)-H(114)...N(21)	0.95	2.60	3.508(2)	160.7
C(14)-H(14)...N(15)#3	0.95	2.61	3.532(2)	162.3

Symmetry transformations used to generate equivalent atoms: #1 x + 1, y + 1, z; #2 x − 1, y − 1, z; #3 –x + 3, −y + 1, −z + 1.

3.5. 4-[hydroxy(4H-1,2,4-triazol-4-ylamino)methyl]benzonitrile (3)

3.5.1. Synthesis

Acetonitrilic solution (3 mL) of 4-formylbenzonitrile (49 mg) was added to an acetonitrilic solution (3 mL) of 4-amino-1,2,4-triazole (31 mg). The reaction mixture after complete dissolution was stirred for 2 hours at room temperature (20 °C). The title compound crystallised directly from the mother liquor. Upon standing 2 days at the room temperature, the solution deposited colourless crystal blocks. The crystals were filtered off, washed with a small amount of acetonitrile and diethyl ether then dried in the air to afford 4-[hydroxy(4*H*-1,2,4-triazol-4-ylamino)methyl]benzonitrile—(70 mg, 87%), mp 98 °C.

3.5.2. Elemental Analysis

	% C	% H	% N
Calculated	55.81	4.22	32.54
Found	55.89	4.09	32.48

3.5.3. Mass Spectrometry

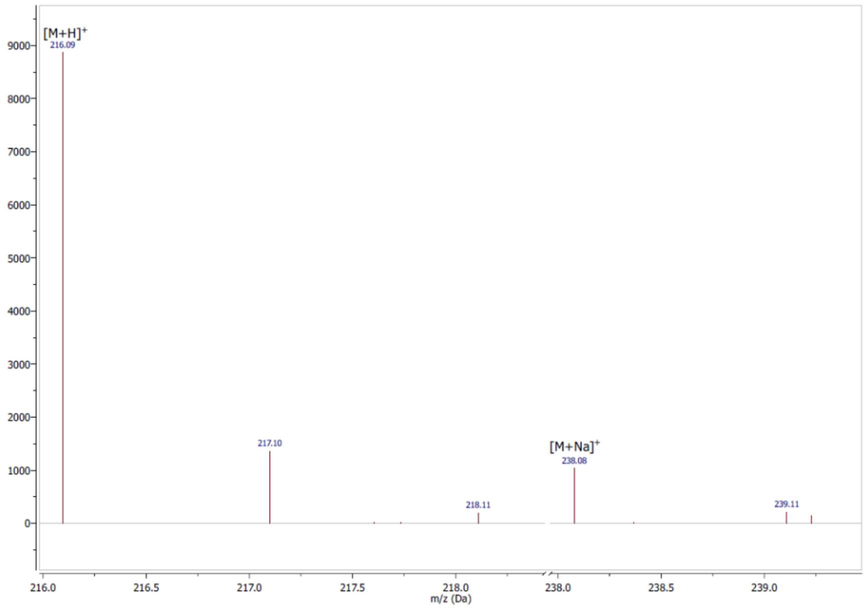

3.5.4. NMR Spectroscopy

^{1}H-NMR

^{1}H-NMR (600 MHz, DMSO, RT): 8.40 (s, 2H, H1T, H2T), 7.84–7.85 (m, 2H, H3, H5), 7.67–7.68 (m, 2H, H2, H6), 7.43 (d, $^{3}J_{H40,H14}$ = 5.5 Hz, 1H, H40), 6.82 (d, $^{3}J_{H41,H14}$ = 4.5 Hz, 1H, H41), 5.59 (pseudo-triplet, $^{3}J_{H14,H40}$ = 5.5 Hz, $^{3}J_{H14,H41}$ = 4.5 Hz, 1H, H14).

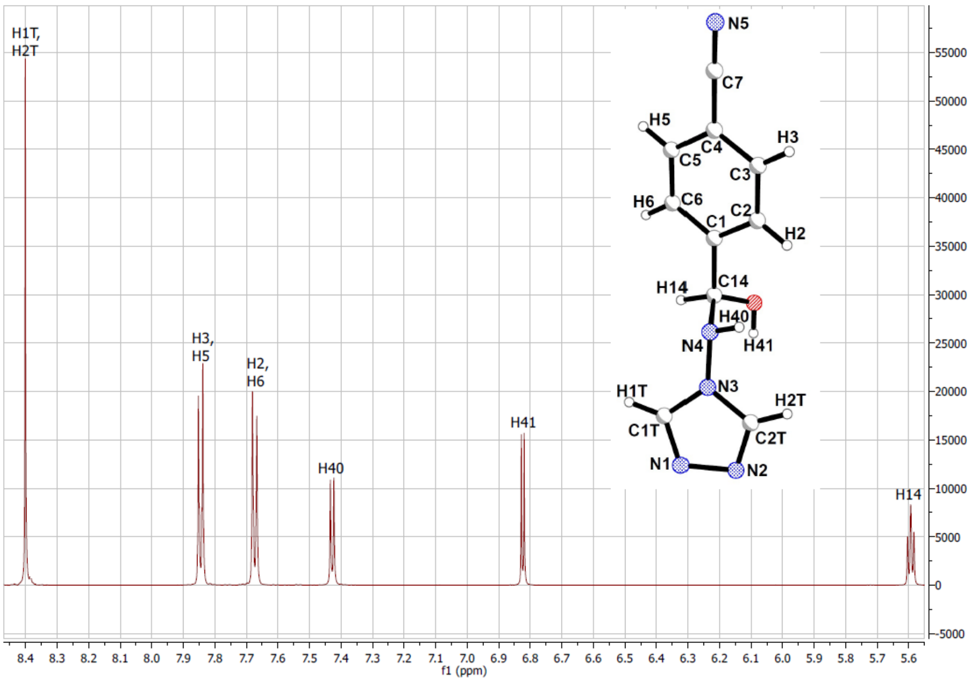

^{13}C-NMR

^{13}C-NMR (150.9 MHz, DMSO, RT): 145.2 (C1), 143.9 (C1T, C2T), 132.1 (C3, C5), 127.7 (C2, C6), 118.7 (C7), 110.8 (C4), 82.8 (C14).

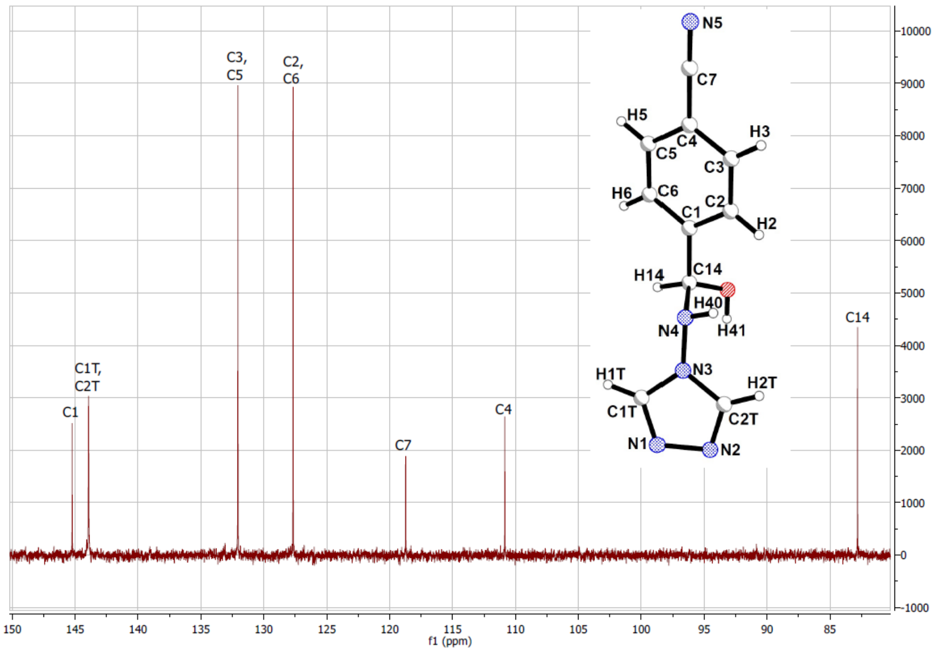

HMQC

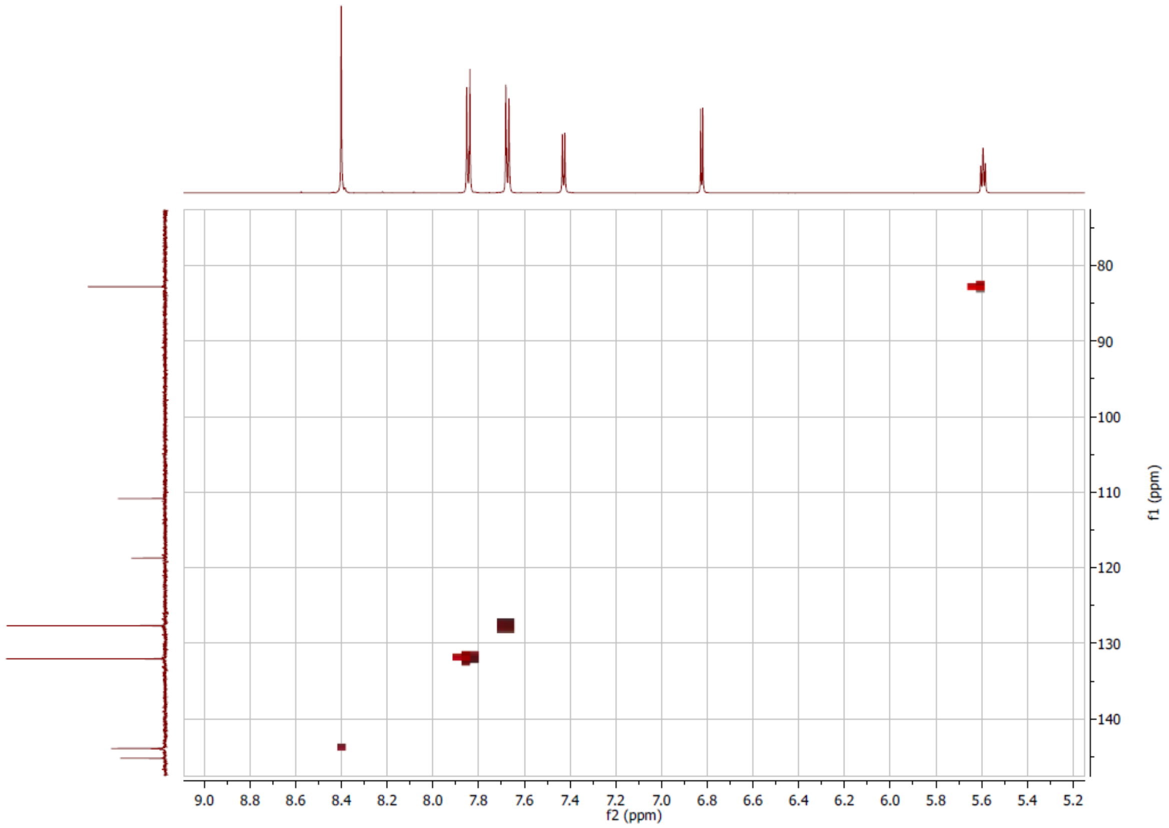

HMBC

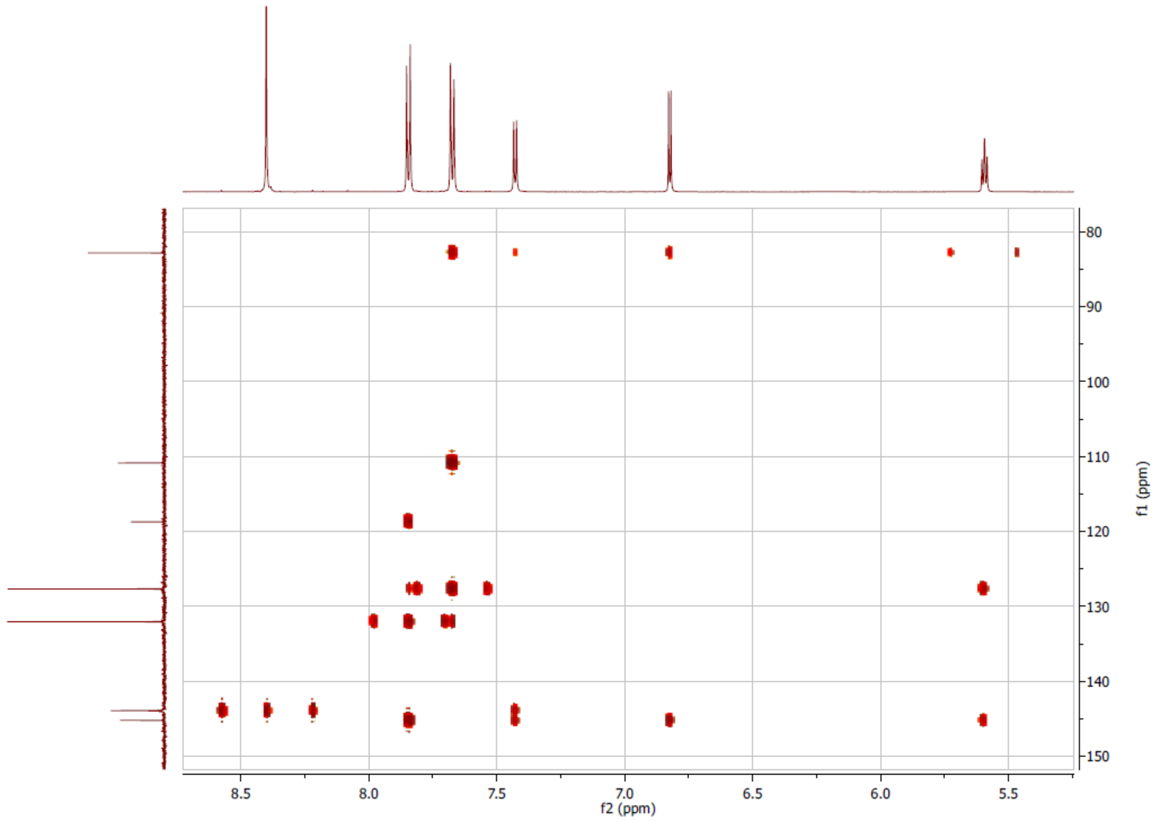

3.5.5. IR Spectroscopy

IR (KBr, cm^{-1}): 3203s, 3111s, 2852m, 2234s, 1952vw, 1830vw, 1773vw, 1734vw, 1700vw, 1685vw, 1653vw, 1610w, 1554m, 1504s, 1460w, 1406m, 1364w, 1340w, 1316m, 1296m, 1270m, 1205s, 1174w, 1163m, 1111vw, 1093vw, 1067vs, 1055vs, 1019s, 977m, 950s, 936w, 910w, 892m, 864s, 846s, 824s, 773m, 700w, 680w, 641vs, 616s, 590w, 552s, 522m, 483w, 416w, 375vw.

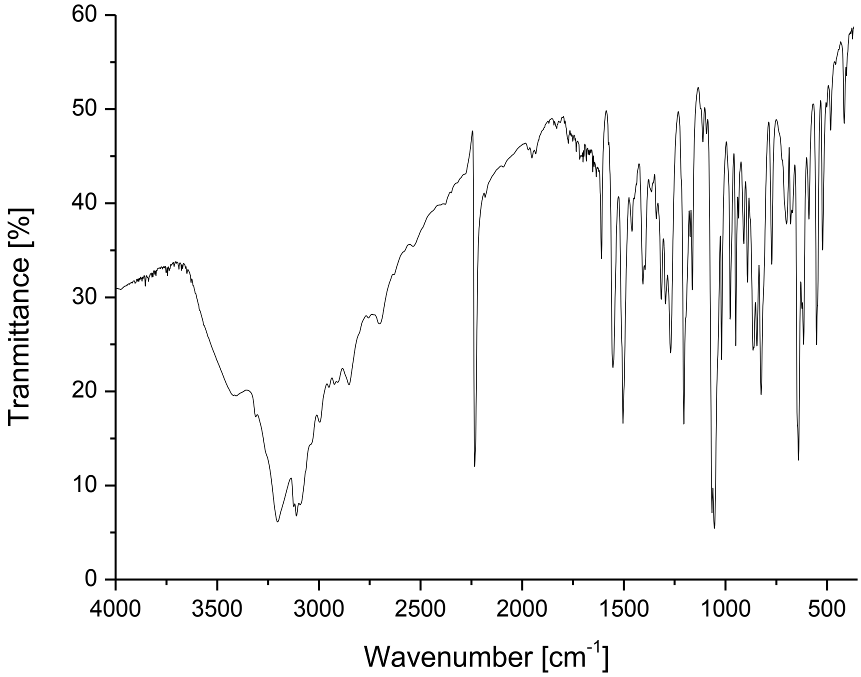

3.5.6. Crystallography

Figure 6. Molecular structure and labelling for 4-[hydroxy(4*H*-1,2,4-triazol-4-ylamino)methyl]benzonitrile (3). Displacement ellipsoids are shown at the 50% probability level.

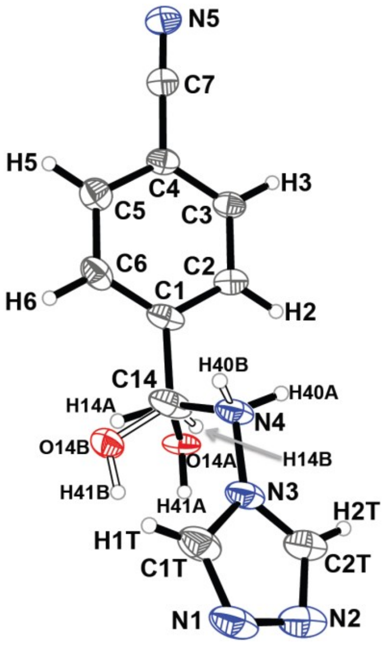

Table 1. Crystal data and structure refinement for 3.

Identification code	3	
Empirical formula	C10 H9 N5 O	
Formula weight	215.22	
Temperature	100(2) K	
Wavelength	0.71073 Å	
Crystal system	Monoclinic	
Space group	P 1 21/c 1	
Unit cell dimensions	a = 12.485(4) Å	α = 90°.
	b = 7.062(3) Å	β = 96.25(3)°.
	c = 11.406(4) Å	γ = 90°.
Volume	999.7(6) Å^3	
Z	4	
Density (calculated)	1.430 Mg/m^3	
Absorption coefficient	0.101 mm^{-1}	
F(000)	448	
Crystal size	0.40 × 0.30 × 0.19 mm^3	
Theta range for data collection	3.28 to 28.68°.	
Index ranges	−15 <= h <= 16, −9 <= k <= 9, −15 <= l <= 15	
Reflections collected	6999	
Independent reflections	2412 [R(int) = 0.0438]	
Completeness to theta = 27.00°	99.3%	
Absorption correction	Semi-empirical from equivalents	
Max. and min. transmission	1.00000 and 0.94543	
Refinement method	Full-matrix least-squares on F^2	
Data/restraints/parameters	2412/0/155	
Goodness-of-fit on F^2	1.020	
Final R indices [I > 2sigma(I)]	R1 = 0.0615, wR2 = 0.1503	
R indices (all data)	R1 = 0.0993, wR2 = 0.1644	
Largest diff. peak and hole	0.499 and −0.307 e.Å^{-3}	

Table 2. Atomic coordinates (×10^4) and equivalent isotropic displacement parameters (Å^2 × 10^3) for 3. U(eq) is defined as one third of the trace of the orthogonalized U^{ij} tensor.

	x	y	z	U(eq)
C(1)	2865(2)	814(3)	819(2)	28(1)
C(2)	3908(2)	1249(3)	570(2)	28(1)
C(3)	4739(2)	1372(3)	1471(2)	26(1)
C(4)	4527(2)	1055(3)	2628(2)	25(1)
C(5)	3484(2)	639(3)	2888(2)	31(1)
C(6)	2655(2)	529(3)	1979(2)	31(1)
C(7)	5406(2)	1171(3)	3567(2)	27(1)
N(5)	6110(2)	1268(3)	4299(2)	33(1)
C(14A)	1989(2)	596(3)	−195(2)	38(1)
O(14A)	1823(2)	2199(3)	−749(2)	28(1)
N(4A)	2323(1)	−990(3)	−904(2)	31(1)
C(14B)	1989(2)	596(3)	−195(2)	38(1)

Table 2. *Cont.*

	x	y	z	U(eq)
O(14B)	1030(3)	752(6)	1(4)	36(1)
N(4B)	2323(1)	−990(3)	−904(2)	31(1)
N(3)	1553(1)	−1395(3)	−1868(2)	32(1)
C(1T)	619(2)	−2397(3)	−1902(2)	39(1)
N(1)	169(2)	−2455(3)	−2997(2)	44(1)
N(2)	818(2)	−1455(3)	−3702(2)	43(1)
C(2T)	1636(2)	−850(3)	−2994(2)	37(1)

Table 3. Bond lengths [Å] and angles [°] for 3.

C(1)-C(6)	1.391(3)
C(1)-C(2)	1.397(3)
C(1)-C(14A)	1.511(3)
C(2)-C(3)	1.381(3)
C(2)-H(2)	0.9500
C(3)-C(4)	1.392(3)
C(3)-H(3)	0.9500
C(4)-C(5)	1.397(3)
C(4)-C(7)	1.450(3)
C(5)-C(6)	1.385(3)
C(5)-H(5)	0.9500
C(6)-H(6)	0.9500
C(7)-N(5)	1.147(3)
C(14A)-O(14A)	1.302(3)
C(14A)-N(4A)	1.469(3)
C(14A)-H(14A)	1.0000
O(14A)-H(41A)	0.8400
N(4A)-N(3)	1.409(2)
N(4A)-H(40A)	0.8871
N(4A)-H(40B)	0.8760
O(14B)-H(41B)	0.8400
N(3)-C(2T)	1.356(3)
N(3)-C(1T)	1.361(3)
C(1T)-N(1)	1.313(3)
C(1T)-H(1T)	0.9500
N(1)-N(2)	1.394(3)
N(2)-C(2T)	1.303(3)
C(2T)-H(2T)	0.9500
C(6)-C(1)-C(2)	120.06(19)
C(6)-C(1)-C(14A)	121.2(2)
C(2)-C(1)-C(14A)	118.7(2)
C(3)-C(2)-C(1)	120.3(2)
C(3)-C(2)-H(2)	119.9
C(1)-C(2)-H(2)	119.9

Table 3. *Cont.*

C(2)-C(3)-C(4)	119.36(19)
C(2)-C(3)-H(3)	120.3
C(4)-C(3)-H(3)	120.3
C(3)-C(4)-C(5)	120.81(19)
C(3)-C(4)-C(7)	118.98(18)
C(5)-C(4)-C(7)	120.2(2)
C(6)-C(5)-C(4)	119.4(2)
C(6)-C(5)-H(5)	120.3
C(4)-C(5)-H(5)	120.3
C(5)-C(6)-C(1)	120.11(19)
C(5)-C(6)-H(6)	119.9
C(1)-C(6)-H(6)	119.9
N(5)-C(7)-C(4)	179.1(2)
O(14A)-C(14A)-N(4A)	115.8(2)
O(14A)-C(14A)-C(1)	110.3(2)
N(4A)-C(14A)-C(1)	105.90(16)
O(14A)-C(14A)-H(14A)	108.2
N(4A)-C(14A)-H(14A)	108.2
C(1)-C(14A)-H(14A)	108.2
C(14A)-O(14A)-H(41A)	109.5
N(3)-N(4A)-C(14A)	111.97(16)
N(3)-N(4A)-H(40A)	109.3
C(14A)-N(4A)-H(40A)	110.4
N(3)-N(4A)-H(40B)	108.8
C(14A)-N(4A)-H(40B)	106.8
H(40A)-N(4A)-H(40B)	109.5
C(2T)-N(3)-C(1T)	105.9(2)
C(2T)-N(3)-N(4A)	124.42(19)
C(1T)-N(3)-N(4A)	129.7(2)
N(1)-C(1T)-N(3)	108.7(2)
N(1)-C(1T)-H(1T)	125.7
N(3)-C(1T)-H(1T)	125.7
C(1T)-N(1)-N(2)	108.51(19)
C(2T)-N(2)-N(1)	105.9(2)
N(2)-C(2T)-N(3)	111.0(2)
N(2)-C(2T)-H(2T)	124.5
N(3)-C(2T)-H(2T)	124.5

Table 4. Anisotropic displacement parameters ($Å^2 \times 10^3$) for 3. The anisotropic displacement factor exponent takes the form: $-2\pi^2[h^2a^{*2}U^{11} + ... + 2hka^*b^*U^{12}]$.

	U11	U22	U33	U23	U13	U12
C(1)	24(1)	22(1)	38(1)	−6(1)	−9(1)	4(1)
C(2)	28(1)	26(1)	28(1)	2(1)	−5(1)	−1(1)
C(3)	23(1)	23(1)	29(1)	1(1)	−3(1)	−3(1)
C(4)	25(1)	20(1)	28(1)	−4(1)	−4(1)	−1(1)
C(5)	28(1)	29(1)	34(1)	−5(1)	2(1)	−2(1)
C(6)	21(1)	31(1)	40(1)	−10(1)	3(1)	−1(1)
C(7)	28(1)	24(1)	28(1)	1(1)	0(1)	−2(1)
N(5)	33(1)	34(1)	30(1)	0(1)	−5(1)	−3(1)
C(14A)	26(1)	33(1)	51(2)	−11(1)	−13(1)	7(1)
O(14A)	26(1)	26(1)	30(2)	8(1)	−12(1)	0(1)
N(4A)	21(1)	33(1)	35(1)	−6(1)	−10(1)	1(1)
C(14B)	26(1)	33(1)	51(2)	−11(1)	−13(1)	7(1)
O(14B)	21(2)	52(3)	34(2)	−8(2)	−1(2)	−4(2)
N(4B)	21(1)	33(1)	35(1)	−6(1)	−10(1)	1(1)
N(3)	23(1)	32(1)	38(1)	−9(1)	−9(1)	2(1)
C(1T)	26(1)	38(1)	51(2)	−13(1)	−6(1)	−3(1)
N(1)	29(1)	41(1)	59(2)	−16(1)	−13(1)	3(1)
N(2)	41(1)	36(1)	47(1)	−6(1)	−15(1)	4(1)
C(2T)	33(1)	32(1)	43(2)	−4(1)	−11(1)	4(1)

Table 5. Hydrogen coordinates ($\times 10^4$) and isotropic displacement parameters ($Å^2 \times 10^3$) for 3.

	x	y	z	U(eq)
H(2)	4046	1461	−222	33
H(3)	5448	1669	1303	31
H(5)	3345	432	3680	37
H(6)	1943	258	2148	37
H(14A)	1309	228	135	45
H(41A)	1338	2061	−1312	42
H(40A)	2951	−738	−1168	37
H(14B)	2098	1724	−699	45
H(41B)	627	643	−635	54
H(40B)	2391	−1980	−439	37
H(1T)	339	−2961	−1244	47
H(2T)	2214	−124	−3235	44

Table 6. Torsion angles [°] for 3.

C(6)-C(1)-C(2)-C(3)	0.9(3)
C(14A)-C(1)-C(2)-C(3)	−176.98(18)
C(1)-C(2)-C(3)-C(4)	0.1(3)
C(2)-C(3)-C(4)-C(5)	−0.7(3)
C(2)-C(3)-C(4)-C(7)	179.46(18)
C(3)-C(4)-C(5)-C(6)	0.4(3)
C(7)-C(4)-C(5)-C(6)	−179.80(19)

Table 6. *Cont.*

C(4)-C(5)-C(6)-C(1)	0.6(3)
C(2)-C(1)-C(6)-C(5)	−1.3(3)
C(14A)-C(1)-C(6)-C(5)	176.59(19)
C(6)-C(1)-C(14A)-O(14A)	117.6(2)
C(2)-C(1)-C(14A)-O(14A)	−64.5(3)
C(6)-C(1)-C(14A)-N(4A)	−116.4(2)
C(2)-C(1)-C(14A)-N(4A)	61.5(3)
O(14A)-C(14A)-N(4A)-N(3)	−59.5(3)
C(1)-C(14A)-N(4A)-N(3)	177.94(18)
C(14A)-N(4A)-N(3)-C(2T)	101.3(2)
C(14A)-N(4A)-N(3)-C(1T)	−80.3(3)
C(2T)-N(3)-C(1T)-N(1)	0.3(2)
N(4A)-N(3)-C(1T)-N(1)	−178.34(19)
N(3)-C(1T)-N(1)-N(2)	−0.5(3)
C(1T)-N(1)-N(2)-C(2T)	0.4(3)
N(1)-N(2)-C(2T)-N(3)	−0.3(3)
C(1T)-N(3)-C(2T)-N(2)	0.0(3)
N(4A)-N(3)-C(2T)-N(2)	178.72(18)

Table 7. Hydrogen bonds for 3 [Å and °].

D-H...A	d(D-H)	d(H...A)	d(D...A)	<(DHA)
O(14B)-H(41B)...N(1)#1	0.84	2.21	2.885(5)	137.3
O(14A)-H(41A)...N(1)#1	0.84	1.99	2.742(3)	148.9
N(4B)-H(40B)...N(5)#2	0.88	2.49	3.186(3)	136.9

Symmetry transformations used to generate equivalent atoms:#1 −x, y + 1/2, −z − 1/2; #2 −x + 1, y − 1/2, −z + 1/2.

3.6. 4-[(4H-1,2,4-triazol-4-ylimino)methyl]benzonitrile (3s)

3.6.1. Synthesis

Ethanolic solution (3 mL) of 4-formylbenzonitrile (53 mg) was added to an ethanolic solution (3 mL) of 4-amino-1,2,4-triazole (34 mg). Few drops of hydrochloric acid were added to the obtained solution. The reaction mixture after complete dissolution was refluxed for 4 hours. The title compound was deposited directly from the mother liquor in a non-crystalline state. Obtained product was filtered off, washed with a small amount of ethanol and diethyl ether then dried in the air to afford 4-[(4*H*-1,2,4-triazol-4-ylimino)methyl]benzonitrile—(41 mg, 51%), mp 235 °C.

3.6.2. Elemental Analysis

	% C	% H	% N
Calculated	60.91	3.58	35.51
Found	60.58	3.67	35.40

3.6.3. Mass Spectrometry

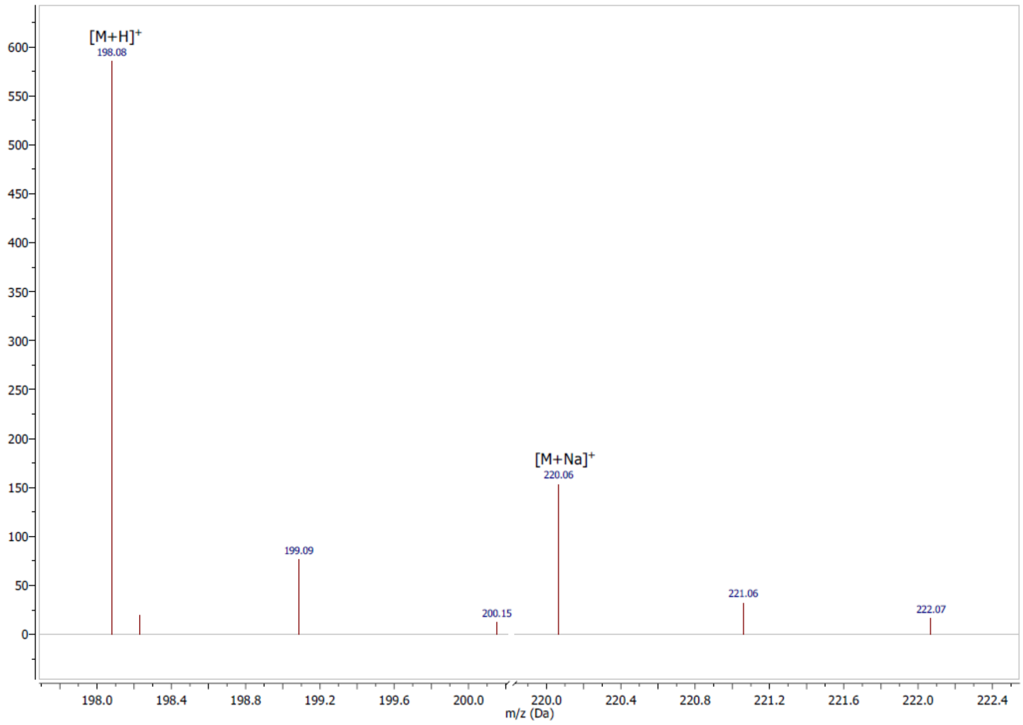

3.6.4. NMR Spectroscopy

^{1}H-NMR

^{1}H-NMR (500 MHz, DMSO, RT): 9.19 (s, 1H, H14), 9.17 (s, 2H, H1T, H2T), 8.00–8.05 (m, 4H, H2, H3, H5, H6).

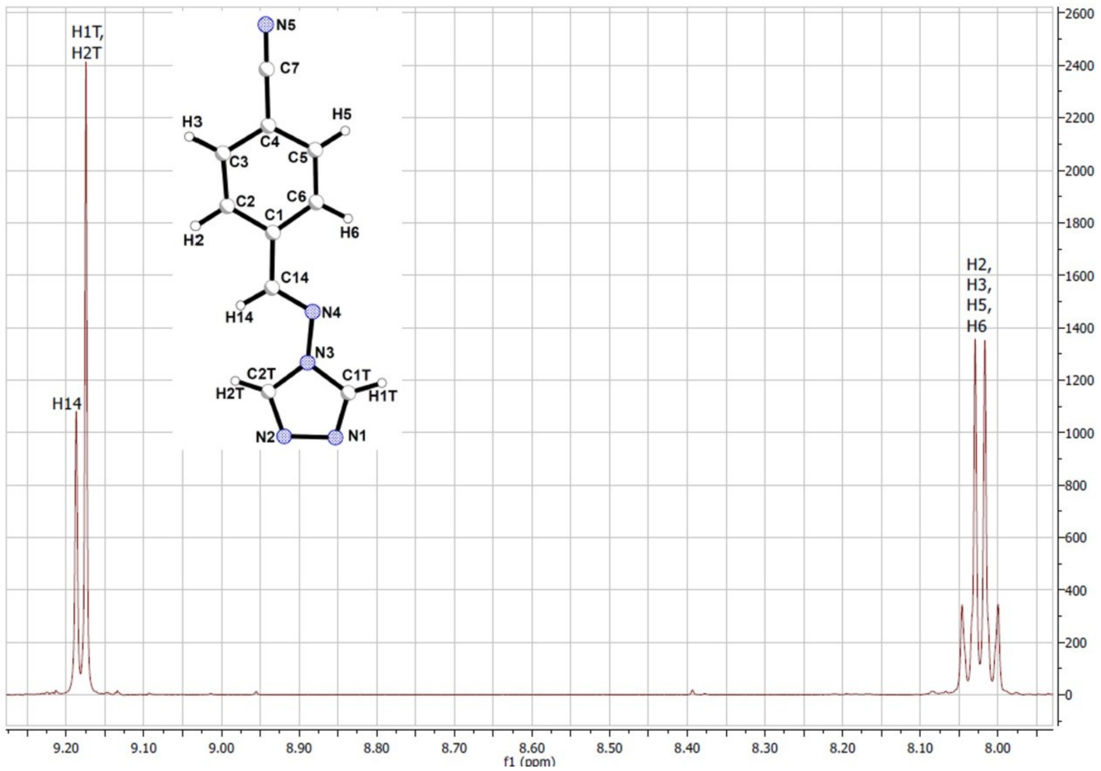

^{13}C-NMR

^{13}C-NMR (125.8 MHz, DMSO, RT): 156.4 (C14), 139.0 (C1T, C2T), 136.4 (C1), 133.1 (C3, C5), 128.8 (C2, C6), 118.3 (C7), 114.0 (C4).

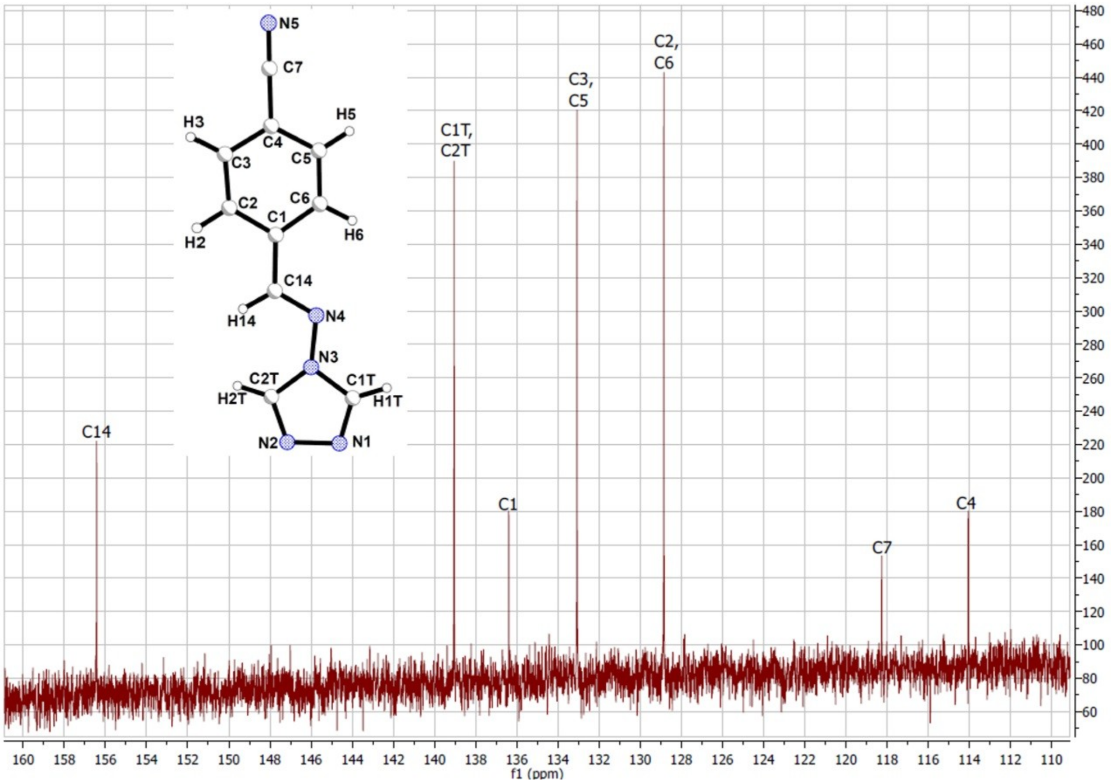

HMQC

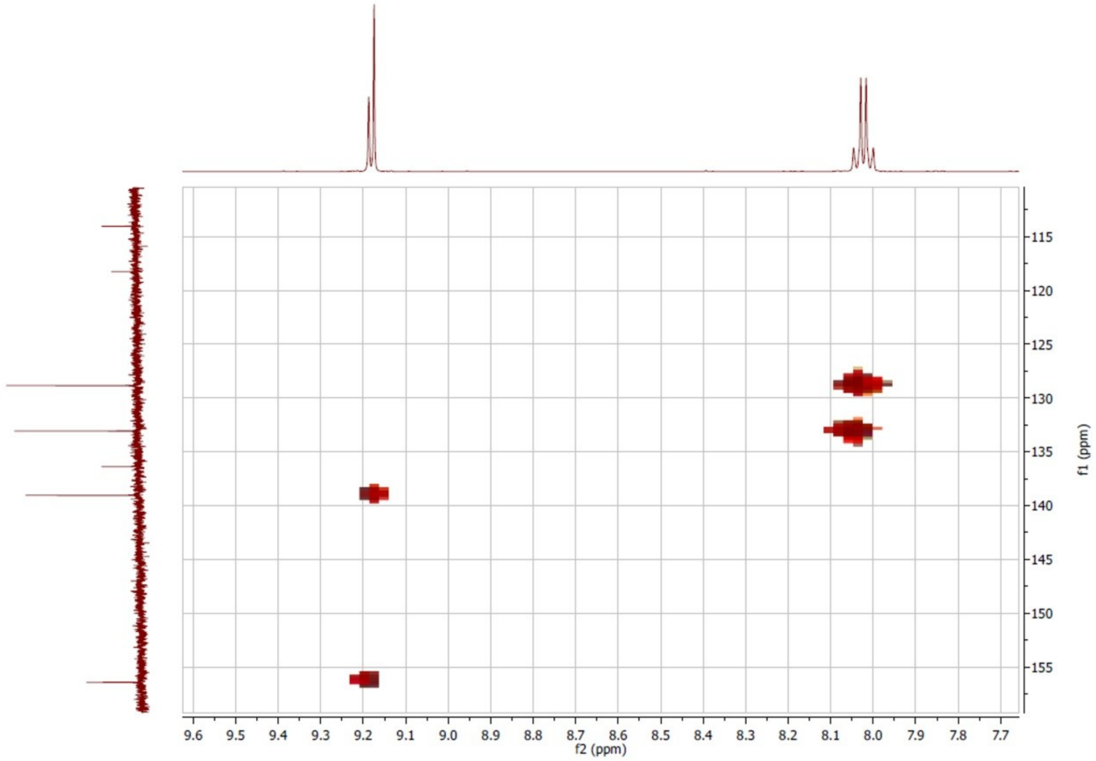

HMBC

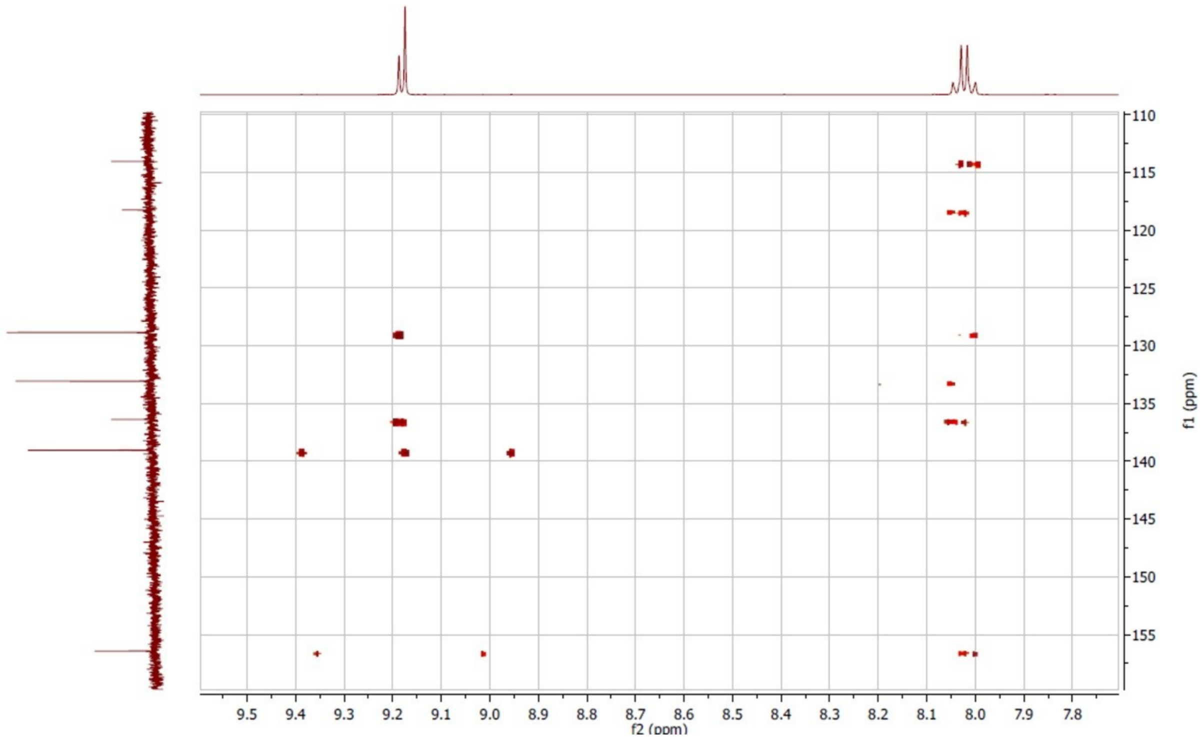

3.6.5. IR Spectroscopy

IR (KBr, cm^{-1}): 3505w, 3092m, 2972m, 2373w, 2229s, 1928w, 1669w, 1616w, 1558vw, 1514s, 1495vs, 1468m, 1414w, 1393s, 1344w, 1328w, 1315m, 1296s, 1281s, 1219s, 1175m, 1163vs, 1108m, 1050vs, 1018m, 981m, 956m, 935s, 881m, 846s, 828vs, 771w, 723w, 701w, 670vw, 647w, 616vs, 552vs, 502w, 483s.

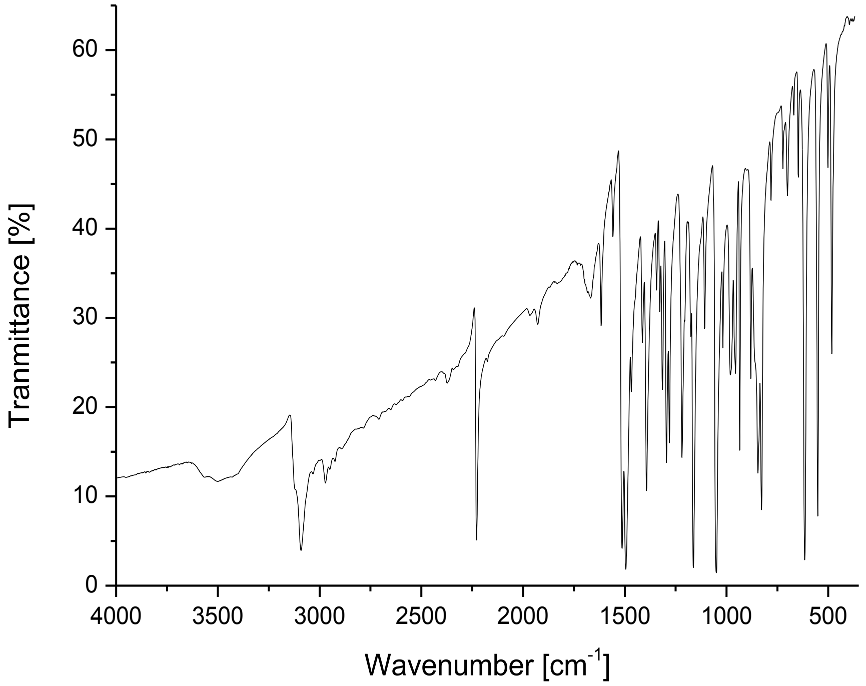

3.7. 3,5-difluoro-4-[hydroxy(4H-1,2,4-triazol-4-ylamino)methyl]benzonitrile (4)

3.7.1. Synthesis

Acetonitrilic solution (3 mL) of 3,5-difluoro-4-formylbenzonitrile (37 mg) was added to an acetonitrilic solution (3 mL) of 4-amino-1,2,4-triazole (19 mg). The reaction mixture after complete dissolution was stirred for 2 hours at 50 °C. The title compound crystallised directly from the mother liquor. Upon standing 3 days at the room temperature, the solution deposited pale yellow crystal plates. The crystals were filtered off, washed with a small amount of acetonitrile and diethyl ether then dried in the air to afford 3,5-difluoro-4-[hydroxy(4*H*-1,2,4-triazol-4-ylamino)methyl]benzonitrile—(51 mg, 90%), mp 131 °C.

3.7.2. Elemental Analysis

	% C	% H	% N
Calculated	47.81	2.81	27.88
Found	47.86	2.63	28.05

3.7.3. Mass Spectrometry

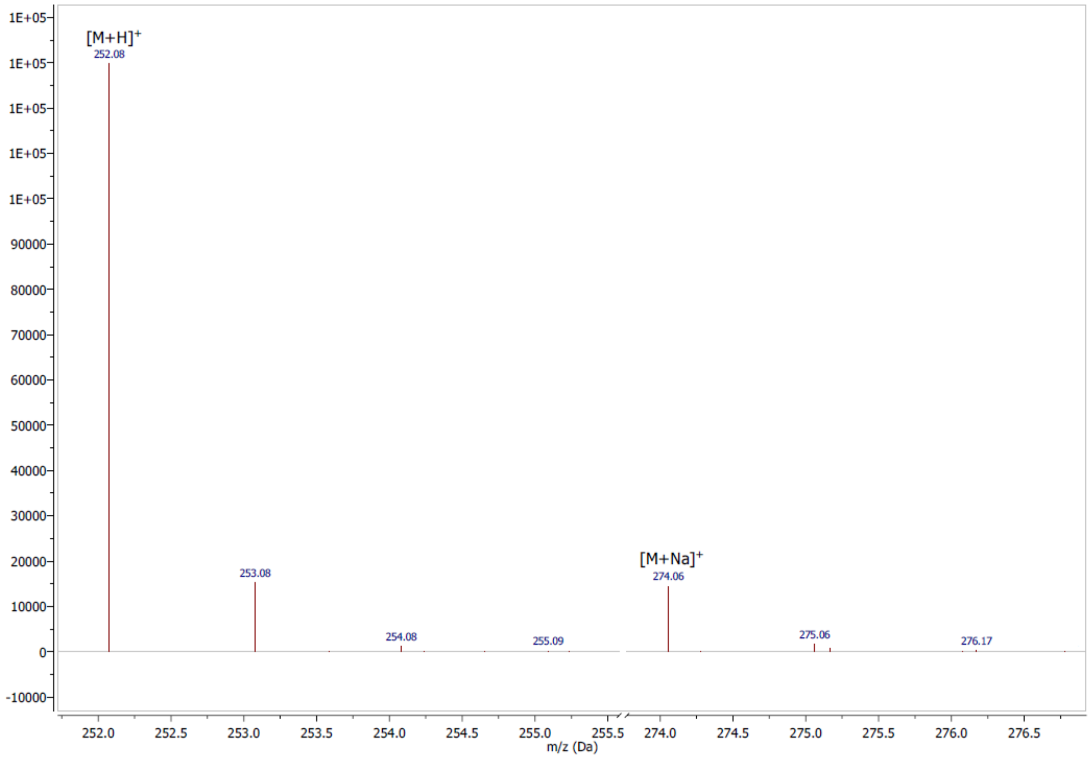

3.7.4. NMR Spectroscopy

^{1}H-NMR

^{1}H-NMR (600 MHz, DMSO, RT): 8.47 (s, 2H, H1T, H2T), 7.82 (d, $^{3}J_{H,F}$ = 7.8 Hz, 2H, H3, H5), 7.62 (d, $^{3}J_{H40,H14}$ = 7.8 Hz, 1H, H40), 7.07 (d, $^{3}J_{H41,H14}$ = 5.4 Hz, 1H, H41), 5.72 (dd, $^{3}J_{H14,H40}$ = 7.8 Hz, $^{3}J_{H14,H41}$ = 5.4 Hz, 1H, H14).

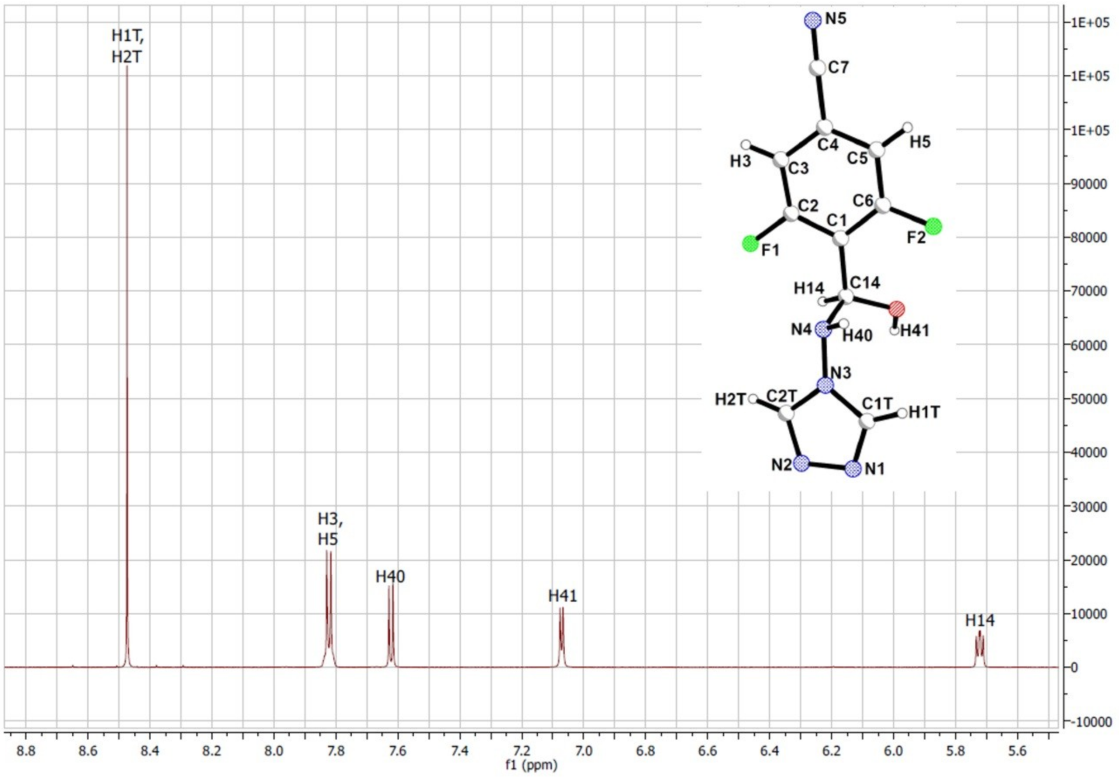

^{13}C-NMR

^{13}C-NMR (150.9 MHz, DMSO, RT): 159.6 (dd, $^{1}J_{C,F}$ = 252.0 Hz, $^{3}J_{C,F}$ = 9.1 Hz, C2, C6), 143.8 (C1T, C2T), 121.5 (t, $^{2}J_{C,F}$ = 17.4 Hz, C1), 116.8 (dd, $^{2}J_{C,F}$ = 24.9 Hz, $^{4}J_{C,F}$ = 6.8 Hz, C3, C5), 116.5 (t, $^{4}J_{C,F}$ = 3.0 Hz, C7), 113.0 (t, $^{3}J_{C,F}$ = 12.8 Hz, C4), 77.6 (C14).

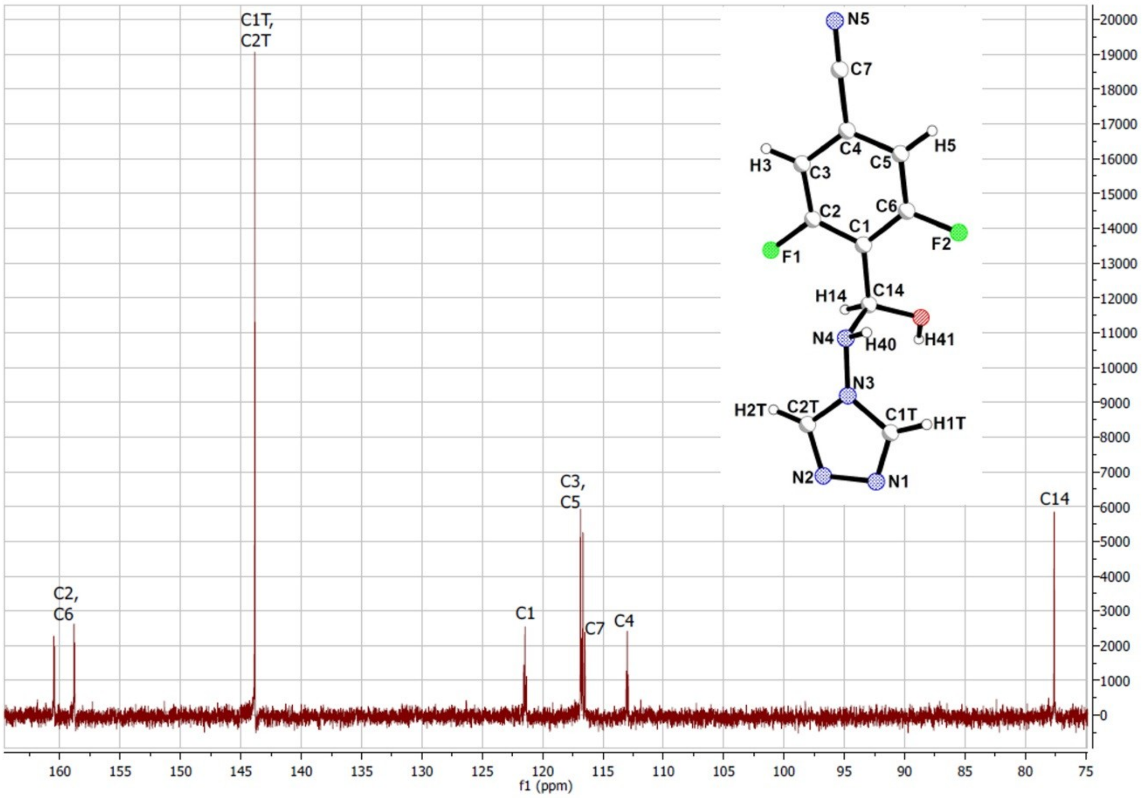

HMQC

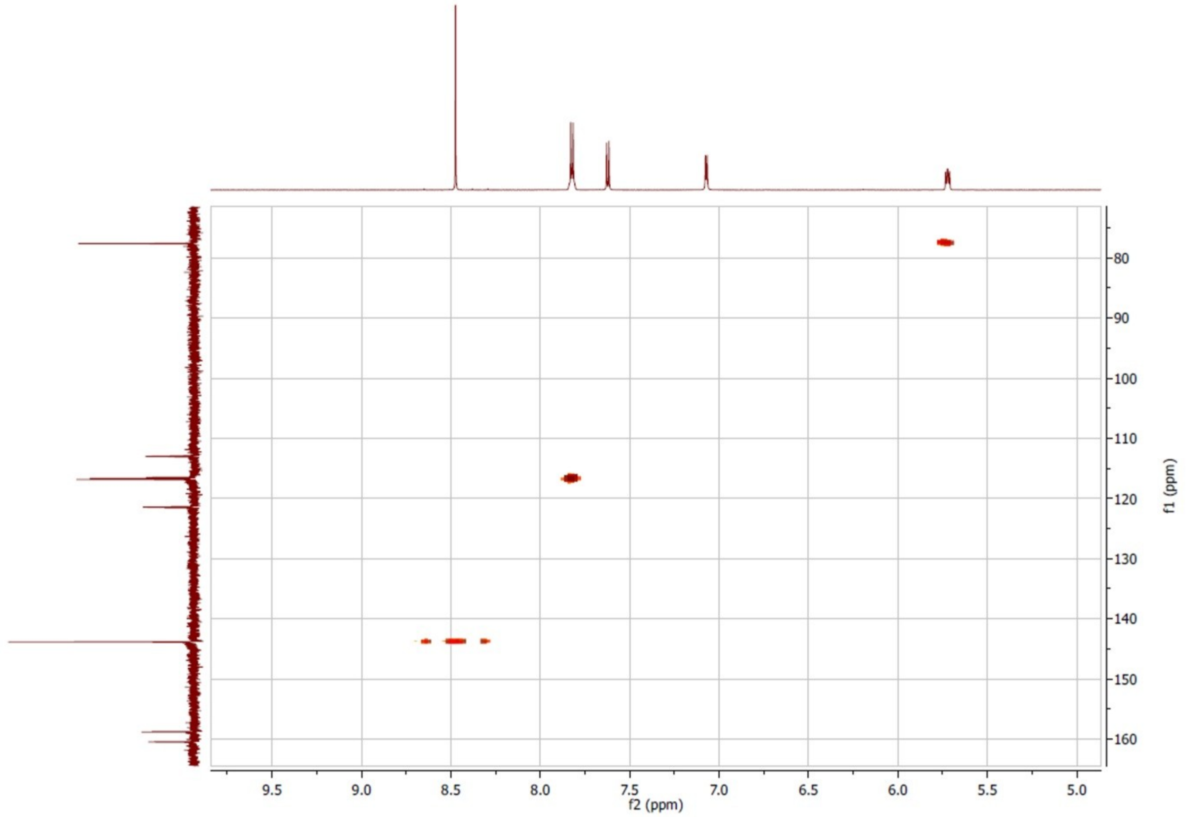
80
90
100
110
120
130
140
150
160
f1 (ppm)
9.5
9.0
8.5
8.0
7.5
7.0
6.5
6.0
5.5
5.0
f2 (ppm)

HMBC

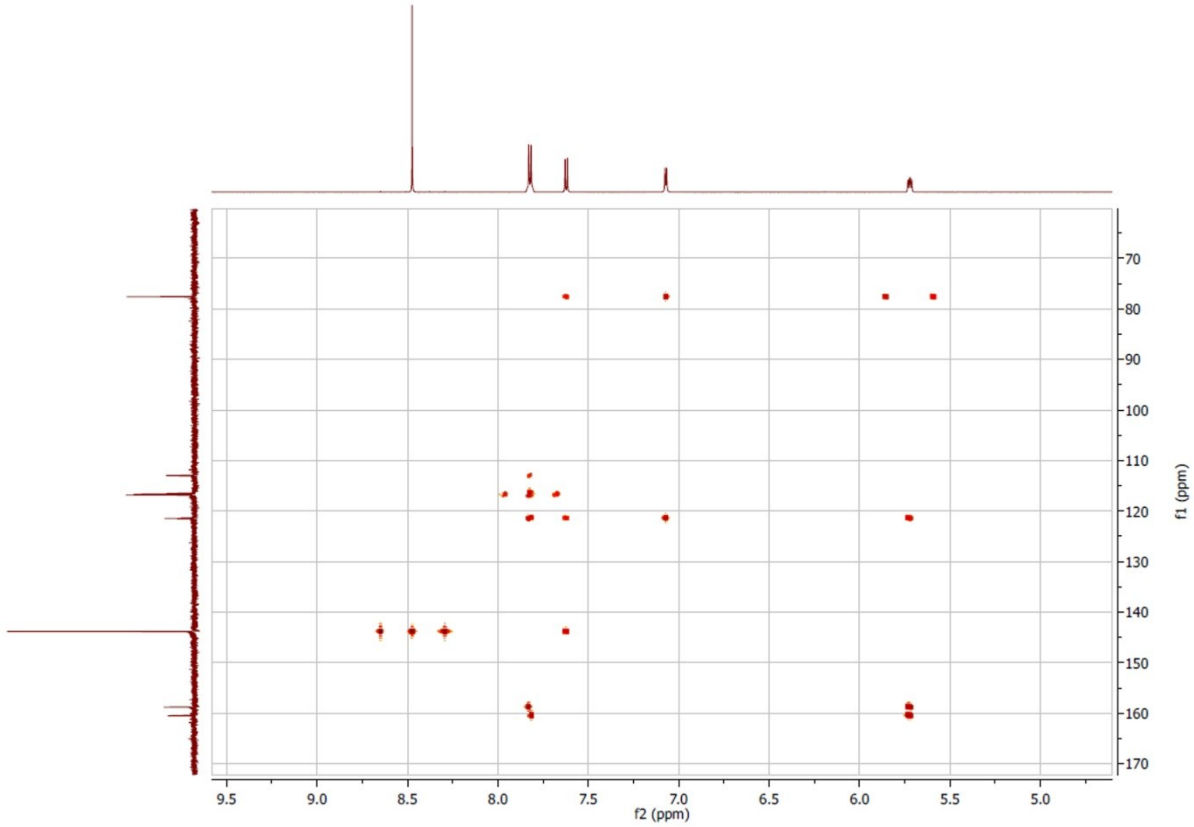
70
80
90
100
110
120
130
140
150
160
170
f1 (ppm)
9.5
9.0
8.5
8.0
7.5
7.0
6.5
6.0
5.5
5.0
f2 (ppm)

3.7.5. IR Spectroscopy

IR (KBr, cm^{-1}): 3284vs, 3129vs, 3087s, 3071vs, 3046m, 2530vw, 2240s, 2190vw, 1763w, 1700vw, 1633vs, 1570vs, 1524vs, 1498m, 1484s, 1455m, 1426vs, 1331s, 1318vs, 1290m, 1260m, 1212vs, 1193s, 1178s, 1128s, 1077s, 1066vs, 1028vs, 974m, 956s, 942m, 904vs, 879vs, 858s, 765w, 738m, 709m, 683m, 649s, 636vs, 628vs, 594m, 550s, 535s, 517s, 484w, 446w, 415w.

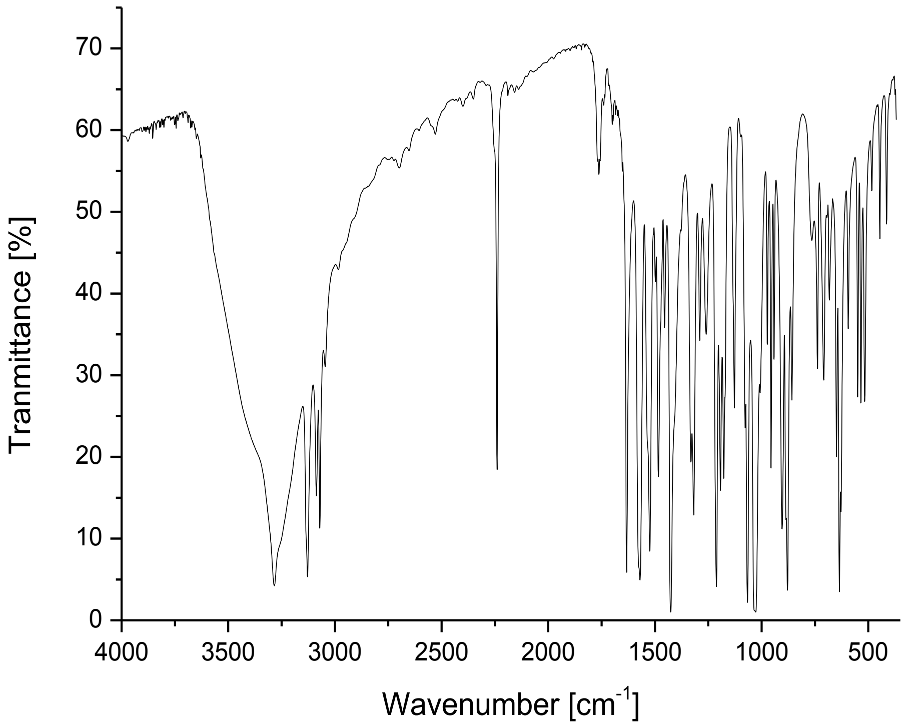

3.7.6. Crystallography

Figure 7. Molecular structure and labelling for 3,5-difluoro-4-[hydroxy(4*H*-1,2,4-triazol-4-ylamino)methyl]benzonitrile hemihydrate (4). Displacement ellipsoids are shown at the 50% probability level.

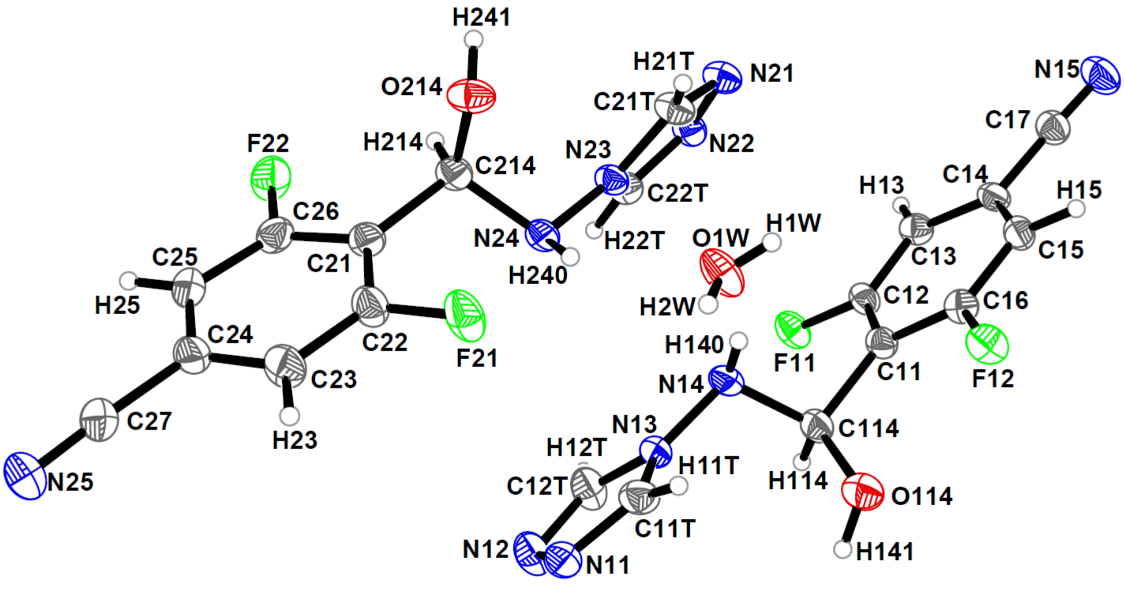

Table 1. Crystal data and structure refinement for 4.

Identification code	4	
Empirical formula	C20 H16 F4 N10 O3	
Formula weight	520.43	
Temperature	100(2) K	
Wavelength	0.71073 Å	
Crystal system	Triclinic	
Space group	P -1	
Unit cell dimensions	a = 7.456(3) Å	α = 80.83(3)°.
	b = 7.624(3) Å	β = 85.83(3)°.
	c = 21.534(5) Å	γ = 67.98(3)°.
Volume	1120.2(7) $Å^3$	
Z	2	
Density (calculated)	1.543 Mg/m^3	
Absorption coefficient	0.132 mm^{-1}	
F(000)	532	
Crystal size	0.40 × 0.25 × 0.10 mm^3	
Theta range for data collection	2.91 to 30.00°.	
Index ranges	−10 <= h <= 9, −10 <= k <= 10, −30 <= l <= 27	
Reflections collected	17252	
Independent reflections	6213 [R(int) = 0.0361]	
Completeness to theta = 27.00°	99.8%	
Absorption correction	Semi-empirical from equivalents	
Max. and min. transmission	1.00000 and 0.98365	
Refinement method	Full-matrix least-squares on F^2	
Data/restraints/parameters	6213/0/344	
Goodness-of-fit on F^2	1.041	
Final R indices [I > 2sigma(I)]	R1 = 0.0507, wR2 = 0.1212	
R indices (all data)	R1 = 0.0883, wR2 = 0.1298	
Largest diff. peak and hole	0.802 and −0.458 e.$Å^{-3}$	

Table 2. Atomic coordinates ($\times 10^4$) and equivalent isotropic displacement parameters ($Å^2 \times 10^3$) for 4. U(eq) is defined as one third of the trace of the orthogonalized U^{ij} tensor.

	**x**	**y**	**z**	**U(eq)**
F(11)	4314(2)	9314(2)	1646(1)	28(1)
F(12)	297(2)	8063(2)	333(1)	28(1)
C(11)	2204(2)	8772(2)	1012(1)	21(1)
C(12)	4049(3)	8701(2)	1115(1)	22(1)
C(13)	5621(3)	8080(3)	716(1)	23(1)
C(14)	5313(3)	7484(2)	169(1)	22(1)
C(15)	3513(3)	7480(2)	41(1)	22(1)
C(16)	2010(3)	8114(3)	466(1)	22(1)
C(17)	6873(3)	6905(3)	−285(1)	26(1)
N(15)	8075(3)	6484(3)	−656(1)	35(1)
C(114)	582(3)	9543(3)	1486(1)	24(1)
O(114)	−1245(2)	10236(2)	1219(1)	34(1)

Table 2. *Cont.*

	x	y	z	U(eq)
N(14)	925(2)	8001(2)	2014(1)	25(1)
N(13)	−473(2)	8539(2)	2495(1)	23(1)
C(11T)	−2211(3)	8351(3)	2555(1)	26(1)
N(11)	−3078(2)	8957(2)	3070(1)	27(1)
N(12)	−1885(2)	9595(3)	3359(1)	33(1)
C(12T)	−319(3)	9302(3)	3003(1)	31(1)
F(21)	−1086(2)	3571(2)	3343(1)	36(1)
F(22)	3955(2)	1920(2)	4762(1)	36(1)
C(21)	1505(3)	2700(3)	4028(1)	24(1)
C(22)	−461(3)	3203(3)	3942(1)	27(1)
C(23)	−1808(3)	3362(3)	4423(1)	30(1)
C(24)	−1138(3)	2986(3)	5035(1)	28(1)
C(25)	814(3)	2472(3)	5156(1)	29(1)
C(26)	2065(3)	2363(3)	4649(1)	26(1)
C(27)	−2513(3)	3202(3)	5553(1)	31(1)
N(25)	−3627(3)	3446(3)	5960(1)	38(1)
C(214)	2976(2)	2388(3)	3491(1)	23(1)
O(214)	3066(2)	753(2)	3243(1)	29(1)
N(24)	2431(2)	4110(2)	3019(1)	22(1)
N(23)	3977(2)	3984(2)	2590(1)	20(1)
C(21T)	4600(3)	2932(3)	2112(1)	25(1)
N(21)	6104(2)	3202(2)	1829(1)	26(1)
N(22)	6487(2)	4490(2)	2137(1)	24(1)
C(22T)	5193(2)	4936(3)	2586(1)	22(1)
O(1W)	108(2)	4665(2)	1844(1)	31(1)

Table 3. Bond lengths [Å] and angles [°] for 4.

F(11)-C(12)	1.355(2)
F(12)-C(16)	1.345(2)
C(11)-C(12)	1.388(3)
C(11)-C(16)	1.387(3)
C(11)-C(114)	1.525(2)
C(12)-C(13)	1.380(2)
C(13)-C(14)	1.391(3)
C(13)-H(13)	0.9500
C(14)-C(15)	1.391(3)
C(14)-C(17)	1.445(3)
C(15)-C(16)	1.384(3)
C(15)-H(15)	0.9500
C(17)-N(15)	1.143(2)
C(114)-O(114)	1.394(2)
C(114)-N(14)	1.459(3)
C(114)-H(114)	1.0000
O(114)-H(141)	0.8400

Table 3. *Cont.*

N(14)-N(13)	1.410(2)
N(14)-H(140)	0.9046
N(13)-C(12T)	1.349(2)
N(13)-C(11T)	1.351(2)
C(11T)-N(11)	1.302(2)
C(11T)-H(11T)	0.9500
N(11)-N(12)	1.385(2)
N(12)-C(12T)	1.316(3)
C(12T)-H(12T)	0.9500
F(21)-C(22)	1.354(2)
F(22)-C(26)	1.351(2)
C(21)-C(22)	1.388(3)
C(21)-C(26)	1.385(3)
C(21)-C(214)	1.517(2)
C(22)-C(23)	1.377(3)
C(23)-C(24)	1.390(3)
C(23)-H(23)	0.9500
C(24)-C(25)	1.391(3)
C(24)-C(27)	1.446(3)
C(25)-C(26)	1.375(3)
C(25)-H(25)	0.9500
C(27)-N(25)	1.150(3)
C(214)-O(214)	1.410(2)
C(214)-N(24)	1.468(2)
C(214)-H(214)	1.0000
O(214)-H(241)	0.8400
N(24)-N(23)	1.408(2)
N(24)-H(240)	0.9051
N(23)-C(21T)	1.353(2)
N(23)-C(22T)	1.357(2)
C(21T)-N(21)	1.305(2)
C(21T)-H(21T)	0.9500
N(21)-N(22)	1.389(2)
N(22)-C(22T)	1.307(2)
C(22T)-H(22T)	0.9500
O(1W)-H(1W)	0.75(3)
O(1W)-H(2W)	0.88(3)
C(12)-C(11)-C(16)	115.02(16)
C(12)-C(11)-C(114)	119.72(16)
C(16)-C(11)-C(114)	125.26(16)
F(11)-C(12)-C(13)	117.63(16)
F(11)-C(12)-C(11)	117.15(15)
C(13)-C(12)-C(11)	125.22(16)
C(12)-C(13)-C(14)	116.74(17)
C(12)-C(13)-H(13)	121.6

Table 3. *Cont.*

C(14)-C(13)-H(13)	121.6
C(15)-C(14)-C(13)	121.25(16)
C(15)-C(14)-C(17)	119.10(17)
C(13)-C(14)-C(17)	119.63(17)
C(16)-C(15)-C(14)	118.55(16)
C(16)-C(15)-H(15)	120.7
C(14)-C(15)-H(15)	120.7
F(12)-C(16)-C(15)	116.99(16)
F(12)-C(16)-C(11)	119.82(15)
C(15)-C(16)-C(11)	123.18(17)
N(15)-C(17)-C(14)	178.2(2)
O(114)-C(114)-N(14)	115.96(16)
O(114)-C(114)-C(11)	112.59(15)
N(14)-C(114)-C(11)	105.19(15)
O(114)-C(114)-H(114)	107.6
N(14)-C(114)-H(114)	107.6
C(11)-C(114)-H(114)	107.6
C(114)-O(114)-H(141)	109.5
N(13)-N(14)-C(114)	111.18(14)
N(13)-N(14)-H(140)	108.9
C(114)-N(14)-H(140)	108.8
C(12T)-N(13)-C(11T)	105.77(15)
C(12T)-N(13)-N(14)	126.74(15)
C(11T)-N(13)-N(14)	127.47(16)
N(11)-C(11T)-N(13)	109.88(17)
N(11)-C(11T)-H(11T)	125.1
N(13)-C(11T)-H(11T)	125.1
C(11T)-N(11)-N(12)	107.81(15)
C(12T)-N(12)-N(11)	106.29(16)
N(12)-C(12T)-N(13)	110.23(17)
N(12)-C(12T)-H(12T)	124.9
N(13)-C(12T)-H(12T)	124.9
C(22)-C(21)-C(26)	115.17(17)
C(22)-C(21)-C(214)	123.09(17)
C(26)-C(21)-C(214)	121.58(16)
F(21)-C(22)-C(23)	118.22(17)
F(21)-C(22)-C(21)	117.49(16)
C(23)-C(22)-C(21)	124.29(19)
C(22)-C(23)-C(24)	117.36(18)
C(22)-C(23)-H(23)	121.3
C(24)-C(23)-H(23)	121.3
C(25)-C(24)-C(23)	121.39(17)
C(25)-C(24)-C(27)	119.45(19)
C(23)-C(24)-C(27)	119.12(18)
C(26)-C(25)-C(24)	117.72(19)

Table 3. *Cont.*

C(26)-C(25)-H(25)	121.1
C(24)-C(25)-H(25)	121.1
F(22)-C(26)-C(25)	118.12(18)
F(22)-C(26)-C(21)	117.82(16)
C(25)-C(26)-C(21)	124.05(17)
N(25)-C(27)-C(24)	177.5(2)
O(214)-C(214)-N(24)	112.61(15)
O(214)-C(214)-C(21)	108.32(14)
N(24)-C(214)-C(21)	109.47(15)
O(214)-C(214)-H(214)	108.8
N(24)-C(214)-H(214)	108.8
C(21)-C(214)-H(214)	108.8
C(214)-O(214)-H(241)	109.5
N(23)-N(24)-C(214)	109.57(14)
N(23)-N(24)-H(240)	109.0
C(214)-N(24)-H(240)	109.1
C(21T)-N(23)-C(22T)	105.36(15)
C(21T)-N(23)-N(24)	129.69(15)
C(22T)-N(23)-N(24)	124.94(15)
N(21)-C(21T)-N(23)	110.55(16)
N(21)-C(21T)-H(21T)	124.7
N(23)-C(21T)-H(21T)	124.7
C(21T)-N(21)-N(22)	106.74(15)
C(22T)-N(22)-N(21)	107.36(14)
N(22)-C(22T)-N(23)	109.99(16)
N(22)-C(22T)-H(22T)	125.0
N(23)-C(22T)-H(22T)	125.0
H(1W)-O(1W)-H(2W)	105(2)

Table 4. Anisotropic displacement parameters (Å^2 × 10^3)for 4. The anisotropic displacement factor exponent takes the form: $-2\pi^2$[$h^2a^{*2}U^{11}$ + ... + 2 h k a* b* U^{12}].

	U^{11}	U^{22}	U^{33}	U^{23}	U^{13}	U^{12}
F(11)	28(1)	36(1)	25(1)	−13(1)	3(1)	-14(1)
F(12)	22(1)	35(1)	32(1)	−11(1)	1(1)	−13(1)
C(11)	21(1)	21(1)	21(1)	−3(1)	4(1)	−8(1)
C(12)	26(1)	21(1)	20(1)	−6(1)	3(1)	−10(1)
C(13)	19(1)	24(1)	26(1)	−4(1)	2(1)	−8(1)
C(14)	23(1)	18(1)	23(1)	−4(1)	7(1)	−6(1)
C(15)	27(1)	22(1)	19(1)	−4(1)	2(1)	−10(1)
C(16)	21(1)	22(1)	23(1)	−3(1)	1(1)	−9(1)
C(17)	28(1)	25(1)	25(1)	−5(1)	3(1)	−11(1)
N(15)	36(1)	36(1)	31(1)	−11(1)	12(1)	−13(1)
C(114)	20(1)	29(1)	21(1)	−4(1)	3(1)	−7(1)
O(114)	26(1)	39(1)	33(1)	−8(1)	3(1)	−5(1)
N(14)	21(1)	24(1)	24(1)	−5(1)	7(1)	−4(1)

Table 4. *Cont.*

	U^{11}	U^{22}	U^{33}	U^{23}	U^{13}	U^{12}
N(13)	19(1)	26(1)	20(1)	−2(1)	3(1)	−4(1)
C(11T)	19(1)	28(1)	30(1)	−5(1)	0(1)	−5(1)
N(11)	22(1)	28(1)	27(1)	−2(1)	4(1)	−7(1)
N(12)	29(1)	46(1)	25(1)	−9(1)	4(1)	−15(1)
C(12T)	27(1)	44(1)	24(1)	−8(1)	2(1)	−16(1)
F(21)	21(1)	61(1)	25(1)	−8(1)	3(1)	−15(1)
F(22)	22(1)	47(1)	33(1)	4(1)	−3(1)	−8(1)
C(21)	19(1)	22(1)	29(1)	−2(1)	4(1)	−8(1)
C(22)	23(1)	34(1)	24(1)	−4(1)	4(1)	−11(1)
C(23)	21(1)	37(1)	32(1)	−3(1)	5(1)	−12(1)
C(24)	28(1)	25(1)	28(1)	−3(1)	7(1)	−9(1)
C(25)	31(1)	29(1)	24(1)	2(1)	2(1)	−8(1)
C(26)	20(1)	26(1)	29(1)	2(1)	−1(1)	−7(1)
C(27)	31(1)	30(1)	28(1)	0(1)	3(1)	−11(1)
N(25)	35(1)	42(1)	34(1)	−4(1)	11(1)	−13(1)
C(214)	16(1)	25(1)	26(1)	−4(1)	3(1)	−7(1)
O(214)	19(1)	24(1)	43(1)	−8(1)	6(1)	−8(1)
N(24)	15(1)	25(1)	24(1)	−2(1)	7(1)	−6(1)
N(23)	16(1)	21(1)	22(1)	−5(1)	4(1)	−6(1)
C(21T)	22(1)	23(1)	30(1)	−8(1)	6(1)	−8(1)
N(21)	21(1)	25(1)	30(1)	−8(1)	5(1)	−7(1)
N(22)	19(1)	29(1)	25(1)	−5(1)	5(1)	−9(1)
C(22T)	19(1)	23(1)	25(1)	−3(1)	1(1)	−8(1)
O(1W)	26(1)	47(1)	29(1)	−16(1)	11(1)	−22(1)

Table 5. Hydrogen coordinates ($\times 10^4$) and isotropic displacement parameters ($Å^2 \times 10^3$) for 4.

	**x**	**y**	**z**	**U(eq)**
H(13)	6854	8061	810	27
H(15)	3319	7052	−331	27
H(114)	766	10634	1639	29
H(141)	−1981	11167	1392	52
H(140)	860	6967	1875	29
H(11T)	−2724	7852	2265	32
H(12T)	765	9587	3091	37
H(23)	−3142	3716	4341	36
H(25)	1268	2205	5574	35
H(214)	4273	2151	3661	27
H(241)	4227	20	3215	43
H(240)	1387	4197	2809	27
H(21T)	4023	2111	1998	30
H(22T)	5114	5801	2869	27
H(1W)	400(40)	4380(40)	1525(12)	39(7)
H(2W)	−1050(40)	4610(40)	1913(13)	57(8)

Table 6. Torsion angles [°] for 4.

C(16)-C(11)-C(12)-F(11)	−179.42(15)
C(114)-C(11)-C(12)-F(11)	0.8(2)
C(16)-C(11)-C(12)-C(13)	1.5(3)
C(114)-C(11)-C(12)-C(13)	−178.35(17)
F(11)-C(12)-C(13)-C(14)	−179.05(15)
C(11)-C(12)-C(13)-C(14)	0.1(3)
C(12)-C(13)-C(14)-C(15)	−1.3(3)
C(12)-C(13)-C(14)-C(17)	176.96(16)
C(13)-C(14)-C(15)-C(16)	1.0(3)
C(17)-C(14)-C(15)-C(16)	−177.31(17)
C(14)-C(15)-C(16)-F(12)	−179.00(15)
C(14)-C(15)-C(16)-C(11)	0.7(3)
C(12)-C(11)-C(16)-F(12)	177.85(15)
C(114)-C(11)-C(16)-F(12)	−2.4(3)
C(12)-C(11)-C(16)-C(15)	−1.8(3)
C(114)-C(11)-C(16)-C(15)	177.98(17)
C(12)-C(11)-C(114)-O(114)	155.89(17)
C(16)-C(11)-C(114)-O(114)	−23.9(3)
C(12)-C(11)-C(114)-N(14)	−76.9(2)
C(16)-C(11)-C(114)-N(14)	103.3(2)
O(114)-C(114)-N(14)-N(13)	−55.8(2)
C(11)-C(114)-N(14)-N(13)	179.14(14)
C(114)-N(14)-N(13)-C(12T)	−93.8(2)
C(114)-N(14)-N(13)-C(11T)	88.1(2)
C(12T)-N(13)-C(11T)-N(11)	−0.2(2)
N(14)-N(13)-C(11T)-N(11)	178.19(16)
N(13)-C(11T)-N(11)-N(12)	0.8(2)
C(11T)-N(11)-N(12)-C(12T)	−1.1(2)
N(11)-N(12)-C(12T)-N(13)	1.0(2)
C(11T)-N(13)-C(12T)-N(12)	−0.6(2)
N(14)-N(13)-C(12T)-N(12)	−178.94(17)
C(26)-C(21)-C(22)-F(21)	179.21(17)
C(214)-C(21)-C(22)-F(21)	−5.3(3)
C(26)-C(21)-C(22)-C(23)	−0.3(3)
C(214)-C(21)-C(22)-C(23)	175.16(18)
F(21)-C(22)-C(23)-C(24)	−179.76(18)
C(21)-C(22)-C(23)-C(24)	−0.2(3)
C(22)-C(23)-C(24)-C(25)	0.1(3)
C(22)-C(23)-C(24)-C(27)	177.85(18)
C(23)-C(24)-C(25)-C(26)	0.6(3)
C(27)-C(24)-C(25)-C(26)	−177.17(18)
C(24)-C(25)-C(26)-F(22)	178.06(17)
C(24)-C(25)-C(26)-C(21)	−1.2(3)
C(22)-C(21)-C(26)-F(22)	−178.20(17)
C(214)-C(21)-C(26)-F(22)	6.2(3)
C(22)-C(21)-C(26)-C(25)	1.1(3)

Table 6. *Cont.*

C(214)-C(21)-C(26)-C(25)	−174.49(18)
C(22)-C(21)-C(214)-O(214)	−66.7(2)
C(26)-C(21)-C(214)-O(214)	108.45(19)
C(22)-C(21)-C(214)-N(24)	56.4(2)
C(26)-C(21)-C(214)-N(24)	−128.42(18)
O(214)-C(214)-N(24)-N(23)	−71.44(17)
C(21)-C(214)-N(24)-N(23)	168.01(14)
C(214)-N(24)-N(23)-C(21T)	75.5(2)
C(214)-N(24)-N(23)-C(22T)	−103.54(19)
C(22T)-N(23)-C(21T)-N(21)	0.1(2)
N(24)-N(23)-C(21T)-N(21)	−179.11(17)
N(23)-C(21T)-N(21)-N(22)	0.1(2)
C(21T)-N(21)-N(22)-C(22T)	−0.3(2)
N(21)-N(22)-C(22T)-N(23)	0.4(2)
C(21T)-N(23)-C(22T)-N(22)	−0.3(2)
N(24)-N(23)-C(22T)-N(22)	178.96(15)

Table 7. Hydrogen bonds for 4 [Å and °].

D-H...A	d(D-H)	d(H...A)	d(D...A)	<(DHA)
O(114)-H(141)...N(21)#1	0.84	1.99	2.822(2)	173.8
O(214)-H(241)...N(11)#2	0.84	1.89	2.708(2)	165.4
O(1W)-H(2W)...N(22)#3	0.88(3)	1.89(3)	2.775(2)	175(3)
O(1W)-H(1W)...N(15)#4	0.75(3)	2.18(3)	2.911(2)	166(3)
N(14)-H(140)...O(1W)	0.90	2.04	2.909(2)	159.5
N(24)-H(240)...O(1W)	0.91	2.27	3.055(2)	145.0
C(12T)-H(12T)...O(214)#5	0.95	2.27	3.213(3)	171.0
C(25)-H(25)...N(12)#6	0.95	2.46	3.341(3)	154.7
C(23)-H(23)...N(25)#7	0.95	2.60	3.427(3)	145.3
C(21T)-H(21T)...F(11)#8	0.95	2.31	3.165(2)	150.1
C(11T)-H(11T)...F(11)#3	0.95	2.45	3.153(3)	130.7

Symmetry transformations used to generate equivalent atoms: #1 x − 1, y + 1, z; #2 x + 1,y − 1,z; #3 x − 1, y, z; #4 −x + 1, −y + 1, −z; #5 x, y + 1, z; #6 −x, −y + 1, −z + 1; #7 −x − 1, −y + 1, −z + 1; #8 x, y − 1, z.

3.8. 3,5-difluoro-4-[(E)-(4H-1,2,4-triazol-4-ylimino)methyl]benzonitrile (4s)

3.8.1. Synthesis

Ethanolic solution (3 mL) of 3,5-difluoro-4-formylbenzonitrile (55 mg) was added to an ethanolic solution (3 mL) of 4-amino-1,2,4-triazole (28 mg). Few drops of hydrochloric acid were added to the obtained solution. The reaction mixture after complete dissolution was refluxed for 4 hours. The title compound crystallised directly from the mother liquor. Upon standing 3 days at the room temperature, the solution deposited colourless crystal needles. The crystals were filtered off, washed with a small amount of ethanol and diethyl ether then dried in the air to afford 3,5-difluoro-4-[(*E*)-(4*H*-1,2,4-triazol-4-ylimino)methyl]benzonitrile hemihydrate—(49 mg, 67%), mp 195 °C.

3.8.2. Elemental Analysis

	% C	% H	% N
Calculated	49.59	2.50	28.92
Found	49.38	2.41	28.89

3.8.3. Mass Spectrometry

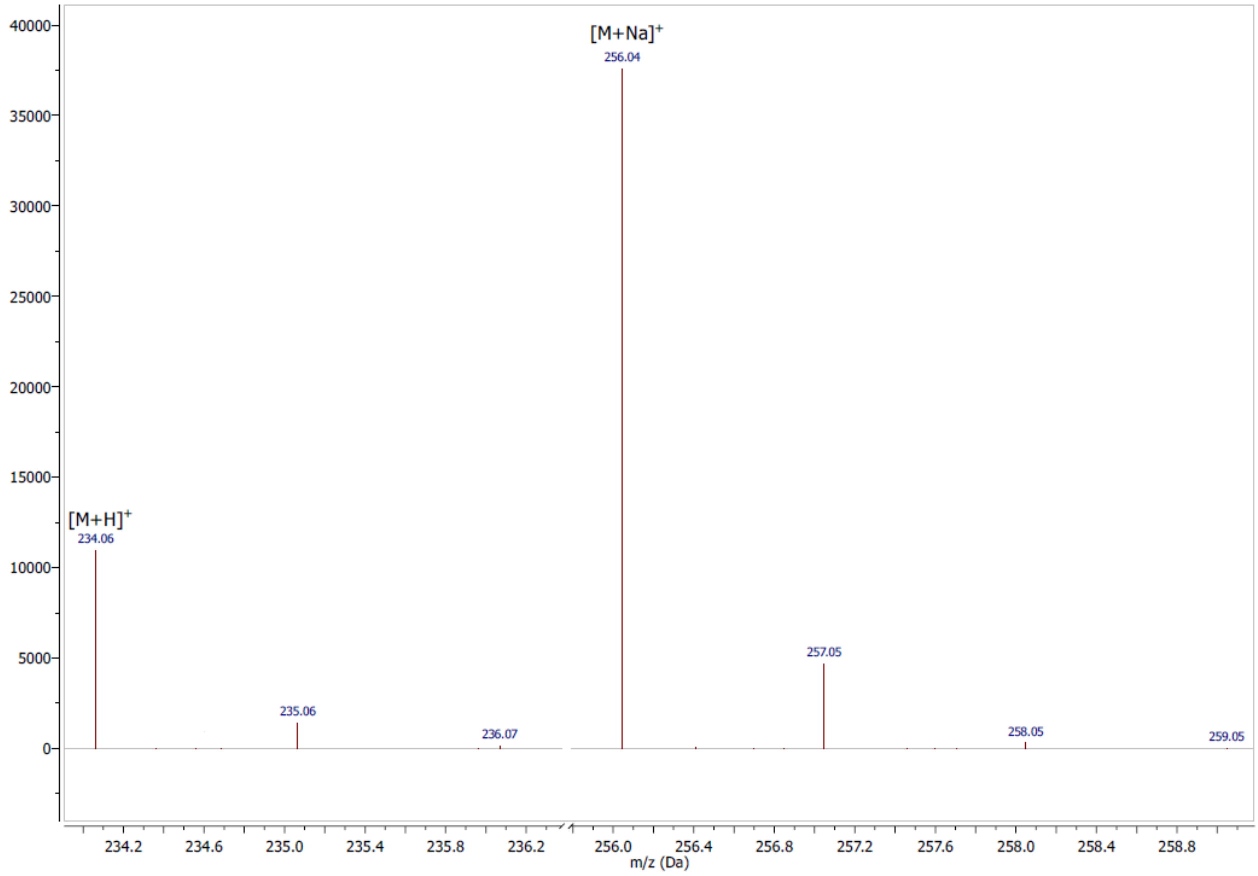

3.8.4. NMR Spectroscopy

^{1}H-NMR

^{1}H-NMR (500 MHz, DMSO, RT): 9.28 (s, 2H, H1T, H2T), 9.10 (s, 1H, H14), 8.00 (d, $^{3}J_{H,F}$ = 8.5 Hz, 2H, H3, H5).

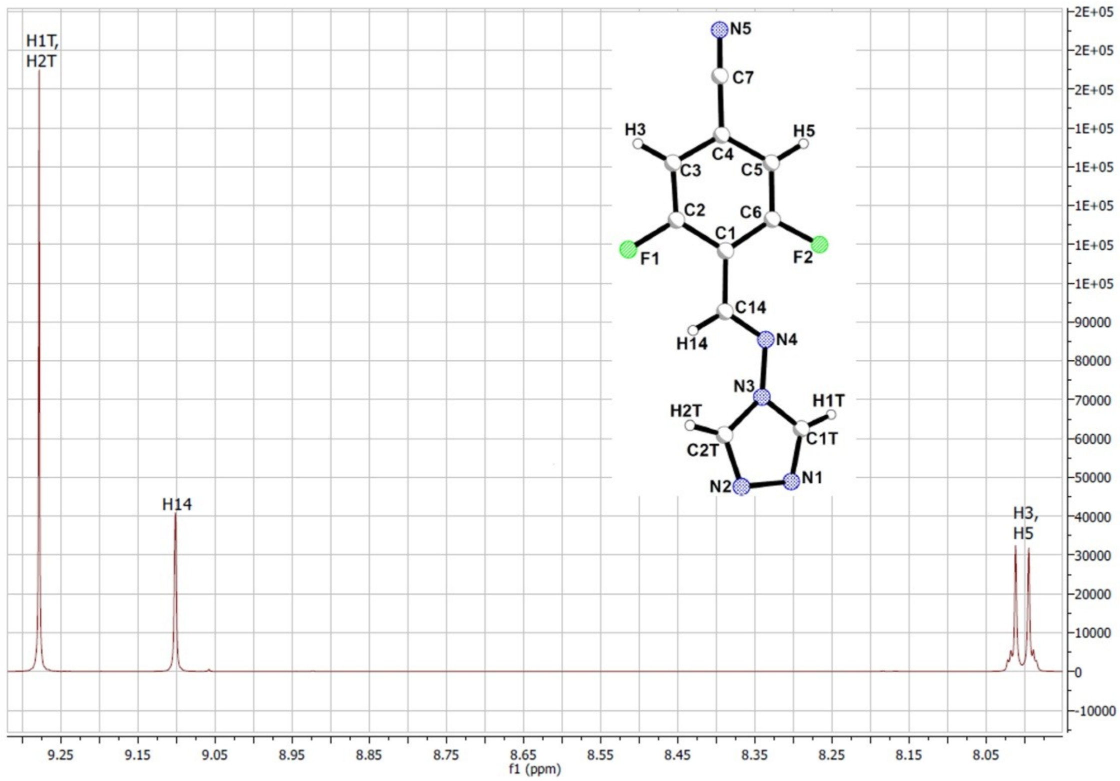

^{13}C-NMR

^{13}C-NMR (125.8 MHz, DMSO, RT): 160.5 (dd, $^{1}J_{C,F}$ = 259.8 Hz, $^{3}J_{C,F}$ = 6.9 Hz, C2, C6), 147.5 (C14), 139.1 (C1T, C2T), 117.2 (dd, $^{2}J_{C,F}$ = 22.6 Hz, $^{4}J_{C,F}$ = 6.3 Hz, C3, C5), 116.3 (t, $^{4}J_{C,F}$ = 3.8 Hz, C7), 115.3 (t, $^{3}J_{C,F}$ = 13.2 Hz, C4), 115.0 (t, $^{2}J_{C,F}$ = 13.8 Hz, C1).

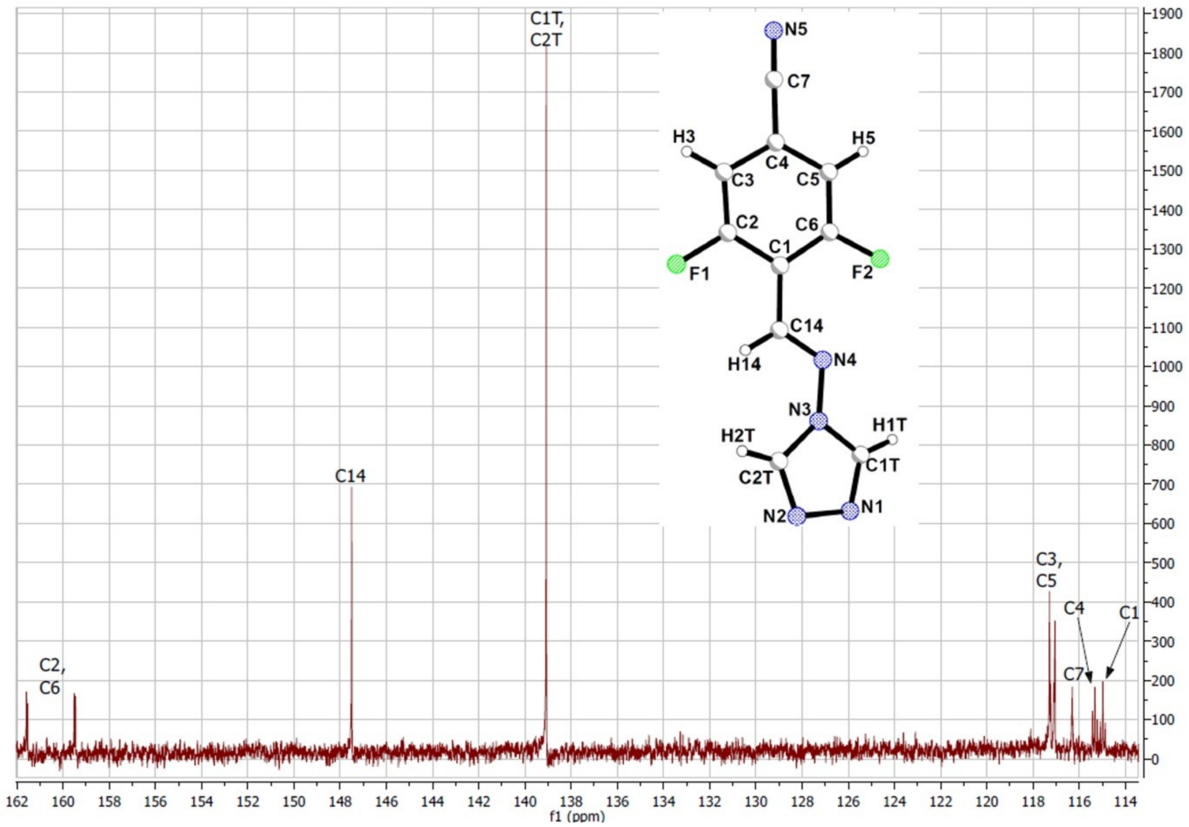

HMQC

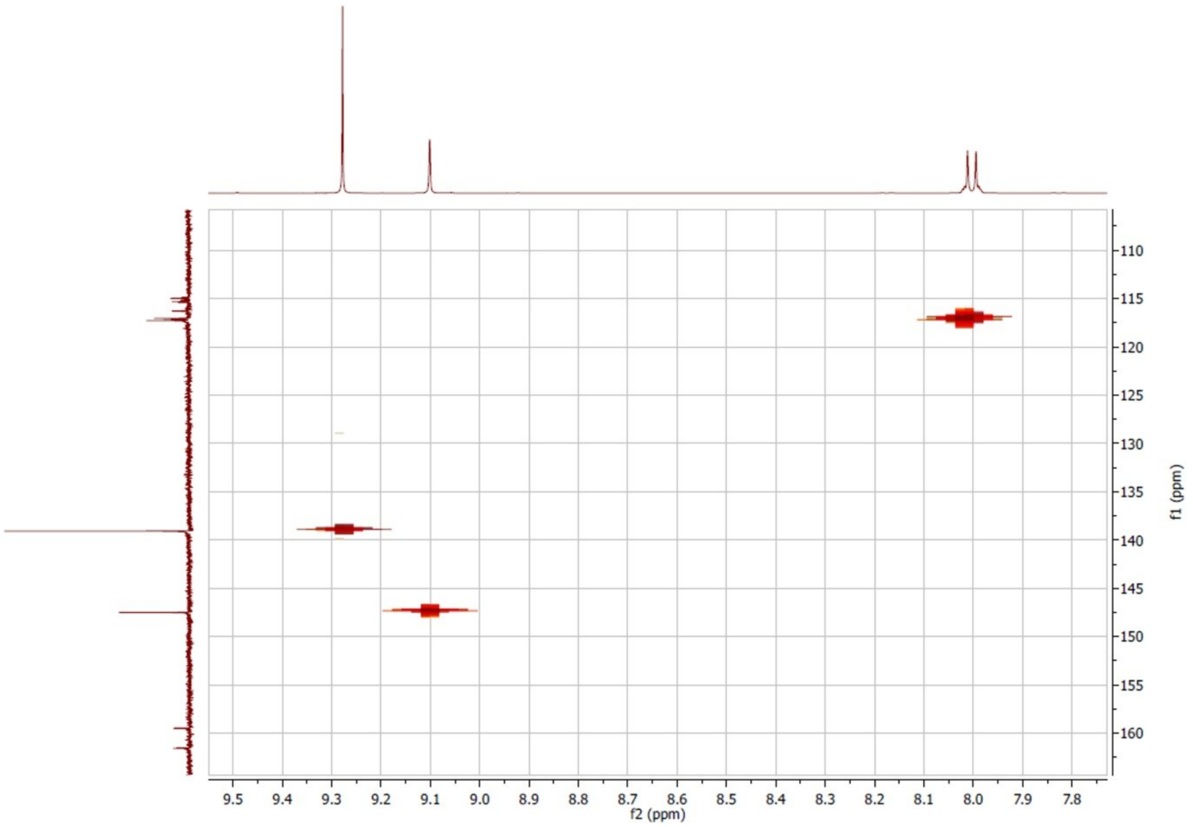

9.5 9.4 9.3 9.2 9.1 9.0 8.9 8.8 8.7 8.6 8.5 8.4 8.3 8.2 8.1 8.0 7.9 7.8
f2 (ppm)
110 115 120 125 130 135 140 145 150 155 160
f1 (ppm)

HMBC

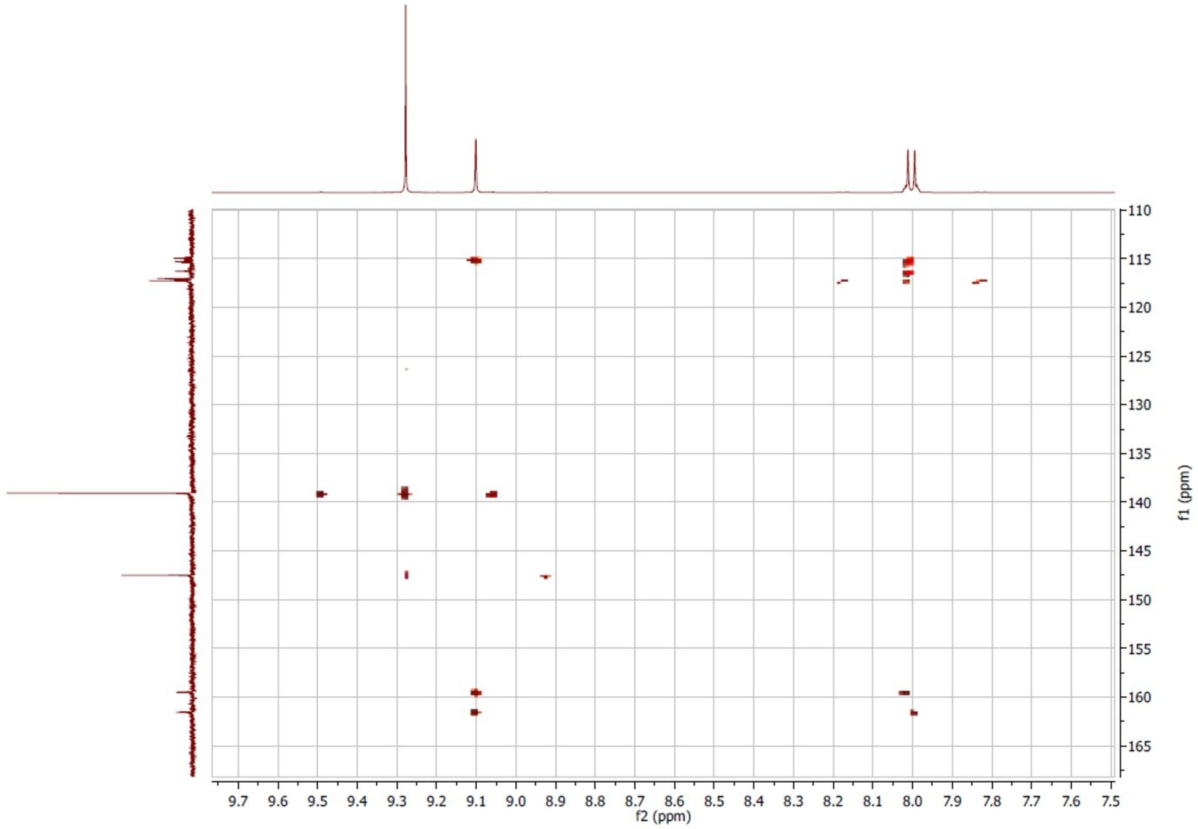

9.7 9.6 9.5 9.4 9.3 9.2 9.1 9.0 8.9 8.8 8.7 8.6 8.5 8.4 8.3 8.2 8.1 8.0 7.9 7.8 7.7 7.6 7.5
f2 (ppm)
110 115 120 125 130 135 140 145 150 155 160 165
f1 (ppm)

3.8.5. IR Spectroscopy

IR (KBr, cm^{-1}): 3369m, 3155m, 3111m, 3081m, 3060m, 2263vw, 2239m, 1780vw, 1717vw, 1627s, 1560s, 1501s, 1469w, 1426vs, 1406m, 1328m, 1296w, 1227w, 1211vs, 1183w, 1164vs, 1062vs, 1042vs, 975w, 962w, 940m, 890s, 869m, 727w, 669vw, 646m, 636s, 619m, 565w, 542m, 495vw, 474m, 467w, 424m, 381vw.

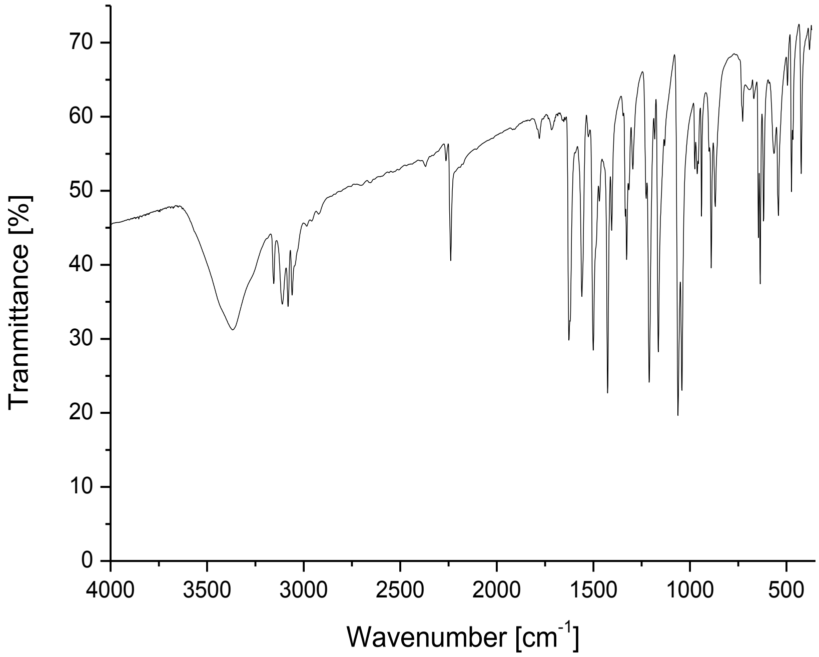

3.8.6. Crystallography

Figure 8. Molecular structure and labelling for 3,5-difluoro-4-[(*E*)-(4*H*-1,2,4-triazol-4-ylimino)methyl]benzonitrile hemihydrate (4s). Displacement ellipsoids are shown at the 50% probability level.

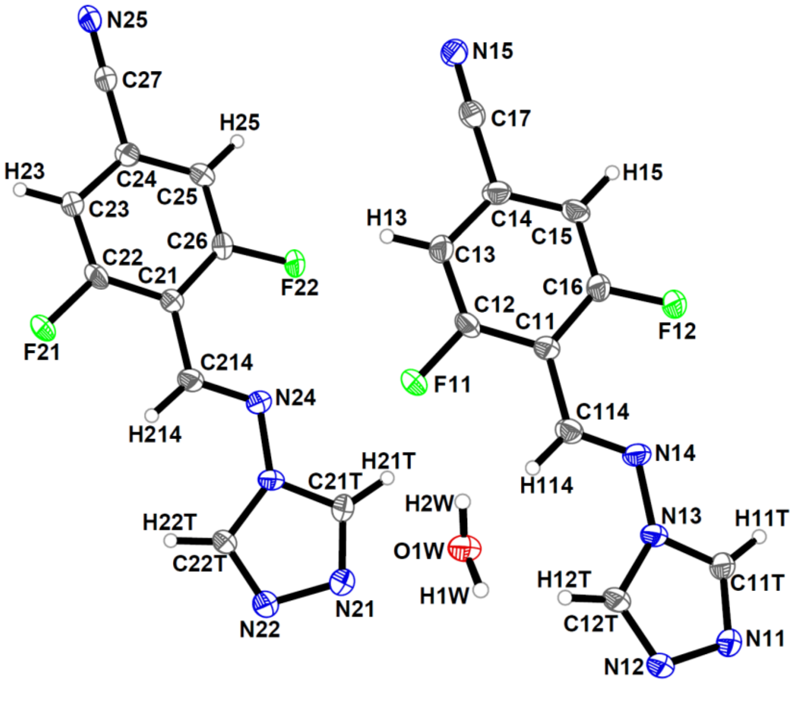

Table 1. Crystal data and structure refinement for 4s.

Identification code	4s	
Empirical formula	C20 H12 F4 N10 O	
Formula weight	484.40	
Temperature	100(2) K	
Wavelength	0.71073 Å	
Crystal system	Monoclinic	
Space group	P 1 21/a 1	
Unit cell dimensions	a = 11.548(3) Å	α = 90°.
	b = 12.346(3) Å	β = 94.98(3)°.
	c = 14.988(4) Å	γ = 90°.
Volume	2128.8(9) $Å^3$	
Z	4	
Density (calculated)	1.511 Mg/m^3	
Absorption coefficient	0.126 mm^{-1}	
F(000)	984	
Crystal size	0.64 × 0.17 × 0.10 mm^3	
Theta range for data collection	2.73 to 28.81°.	
Index ranges	−14 <= h <= 13, −16 <= k <= 7, −20 <= l <= 19	
Reflections collected	8793	
Independent reflections	4799 [R(int) = 0.0464]	
Completeness to theta = 25.00°	99.3%	
Absorption correction	Semi-empirical from equivalents	
Max. and min. transmission	1.00000 and 0.73974	
Refinement method	Full-matrix least-squares on F^2	
Data/restraints/parameters	4799/3/322	
Goodness-of-fit on F^2	1.086	
Final R indices [I > 2sigma(I)]	R1 = 0.0674, wR2 = 0.1634	
R indices (all data)	R1 = 0.1086, wR2 = 0.1966	
Largest diff. peak and hole	0.494 and −0.383 e.$Å^{-3}$	

Table 2. Atomic coordinates ($\times 10^4$) and equivalent isotropic displacement parameters ($Å^2 \times 10^3$) for 4s. U(eq) is defined as one third of the trace of the orthogonalized U^{ij} tensor.

	x	y	z	U(eq)
F(11)	5232(1)	5958(1)	2354(1)	28(1)
F(12)	3866(1)	6428(1)	5201(1)	28(1)
C(11)	4476(2)	6212(2)	3745(2)	20(1)
C(12)	5444(2)	6074(2)	3253(2)	22(1)
C(13)	6579(2)	6043(2)	3618(2)	22(1)
C(14)	6770(2)	6166(2)	4540(2)	23(1)
C(15)	5854(2)	6307(2)	5076(2)	23(1)
C(16)	4734(2)	6315(2)	4670(2)	22(1)
C(17)	7949(2)	6119(2)	4962(2)	24(1)
N(15)	8866(2)	6082(2)	5310(1)	27(1)
C(114)	3309(2)	6258(2)	3283(2)	22(1)
N(14)	2415(2)	6189(2)	3724(1)	23(1)

Table 2. *Cont.*

	x	y	z	U(eq)
N(13)	1338(2)	6246(2)	3233(1)	22(1)
C(11T)	333(2)	6126(2)	3640(2)	23(1)
N(11)	−572(2)	6245(2)	3065(1)	25(1)
N(12)	−146(2)	6456(2)	2233(1)	23(1)
C(12T)	985(2)	6454(2)	2349(2)	24(1)
F(21)	9212(1)	3953(1)	−136(1)	27(1)
F(22)	7823(1)	3763(1)	2727(1)	30(1)
C(21)	8443(2)	3829(2)	1263(2)	20(1)
C(22)	9413(2)	3863(2)	767(2)	22(1)
C(23)	10551(2)	3817(2)	1126(2)	21(1)
C(24)	10733(2)	3725(2)	2054(2)	22(1)
C(25)	9814(2)	3692(2)	2597(2)	24(1)
C(26)	8700(2)	3757(2)	2192(2)	24(1)
C(27)	11920(2)	3714(2)	2474(2)	24(1)
N(25)	12844(2)	3742(2)	2810(1)	29(1)
C(214)	7273(2)	3848(2)	799(2)	23(1)
N(24)	6381(2)	3832(2)	1247(1)	24(1)
N(23)	5304(2)	3816(2)	744(1)	22(1)
C(21T)	4302(2)	3986(2)	1143(2)	26(1)
N(21)	3400(2)	3899(2)	565(1)	27(1)
N(22)	3824(2)	3656(2)	−260(1)	26(1)
C(22T)	4961(2)	3611(2)	−134(2)	23(1)
O(1W)	3014(1)	7452(2)	1215(1)	29(1)

Table 3. Bond lengths [Å] and angles [°] for 4s.

F(11)-C(12)	1.355(3)
F(12)-C(16)	1.341(3)
C(11)-C(16)	1.397(3)
C(11)-C(12)	1.402(3)
C(11)-C(114)	1.463(3)
C(12)-C(13)	1.377(3)
C(13)-C(14)	1.388(3)
C(13)-H(13)	0.9500
C(14)-C(15)	1.394(3)
C(14)-C(17)	1.452(3)
C(15)-C(16)	1.382(3)
C(15)-H(15)	0.9500
C(17)-N(15)	1.140(3)
C(114)-N(14)	1.276(3)
C(114)-H(114)	0.9500
N(14)-N(13)	1.390(3)
N(13)-C(11T)	1.366(3)
N(13)-C(12T)	1.376(3)
C(11T)-N(11)	1.303(3)

Table 3. *Cont.*

C(11T)-H(11T)	0.9500
N(11)-N(12)	1.404(3)
N(12)-C(12T)	1.303(3)
C(12T)-H(12T)	0.9500
F(21)-C(22)	1.358(2)
F(22)-C(26)	1.345(2)
C(21)-C(22)	1.397(3)
C(21)-C(26)	1.401(3)
C(21)-C(214)	1.465(3)
C(22)-C(23)	1.377(3)
C(23)-C(24)	1.394(3)
C(23)-H(23)	0.9500
C(24)-C(25)	1.393(3)
C(24)-C(27)	1.457(3)
C(25)-C(26)	1.376(3)
C(25)-H(25)	0.9500
C(27)-N(25)	1.140(3)
C(214)-N(24)	1.278(3)
C(214)-H(214)	0.9500
N(24)-N(23)	1.397(3)
N(23)-C(22T)	1.364(3)
N(23)-C(21T)	1.365(3)
C(21T)-N(21)	1.301(3)
C(21T)-H(21T)	0.9500
N(21)-N(22)	1.400(3)
N(22)-C(22T)	1.312(3)
C(22T)-H(22T)	0.9500
O(1W)-H(1W)	0.855(10)
O(1W)-H(2W)	0.853(10)
C(16)-C(11)-C(12)	115.0(2)
C(16)-C(11)-C(114)	125.1(2)
C(12)-C(11)-C(114)	119.9(2)
F(11)-C(12)-C(13)	118.4(2)
F(11)-C(12)-C(11)	116.9(2)
C(13)-C(12)-C(11)	124.7(2)
C(12)-C(13)-C(14)	117.1(2)
C(12)-C(13)-H(13)	121.4
C(14)-C(13)-H(13)	121.4
C(13)-C(14)-C(15)	121.7(2)
C(13)-C(14)-C(17)	119.4(2)
C(15)-C(14)-C(17)	118.9(2)
C(16)-C(15)-C(14)	118.3(2)
C(16)-C(15)-H(15)	120.8
C(14)-C(15)-H(15)	120.8
F(12)-C(16)-C(15)	117.3(2)

Table 3. *Cont.*

F(12)-C(16)-C(11)	119.6(2)
C(15)-C(16)-C(11)	123.2(2)
N(15)-C(17)-C(14)	178.6(2)
N(14)-C(114)-C(11)	120.4(2)
N(14)-C(114)-H(114)	119.8
C(11)-C(114)-H(114)	119.8
C(114)-N(14)-N(13)	116.68(19)
C(11T)-N(13)-C(12T)	104.95(19)
C(11T)-N(13)-N(14)	120.93(18)
C(12T)-N(13)-N(14)	134.06(19)
N(11)-C(11T)-N(13)	110.8(2)
N(11)-C(11T)-H(11T)	124.6
N(13)-C(11T)-H(11T)	124.6
C(11T)-N(11)-N(12)	106.62(18)
C(12T)-N(12)-N(11)	107.78(19)
N(12)-C(12T)-N(13)	109.8(2)
N(12)-C(12T)-H(12T)	125.1
N(13)-C(12T)-H(12T)	125.1
C(22)-C(21)-C(26)	114.8(2)
C(22)-C(21)-C(214)	119.8(2)
C(26)-C(21)-C(214)	125.4(2)
F(21)-C(22)-C(23)	117.8(2)
F(21)-C(22)-C(21)	117.2(2)
C(23)-C(22)-C(21)	125.0(2)
C(22)-C(23)-C(24)	116.7(2)
C(22)-C(23)-H(23)	121.6
C(24)-C(23)-H(23)	121.6
C(25)-C(24)-C(23)	121.9(2)
C(25)-C(24)-C(27)	118.9(2)
C(23)-C(24)-C(27)	119.2(2)
C(26)-C(25)-C(24)	118.1(2)
C(26)-C(25)-H(25)	120.9
C(24)-C(25)-H(25)	120.9
F(22)-C(26)-C(25)	117.4(2)
F(22)-C(26)-C(21)	119.1(2)
C(25)-C(26)-C(21)	123.5(2)
N(25)-C(27)-C(24)	177.7(3)
N(24)-C(214)-C(21)	120.2(2)
N(24)-C(214)-H(214)	119.9
C(21)-C(214)-H(214)	119.9
C(214)-N(24)-N(23)	115.94(19)
C(22T)-N(23)-C(21T)	105.38(19)
C(22T)-N(23)-N(24)	133.89(19)
C(21T)-N(23)-N(24)	120.66(19)
N(21)-C(21T)-N(23)	110.8(2)
N(21)-C(21T)-H(21T)	124.6

Table 3. *Cont.*

N(23)-C(21T)-H(21T)	124.6
C(21T)-N(21)-N(22)	106.58(18)
C(22T)-N(22)-N(21)	107.73(19)
N(22)-C(22T)-N(23)	109.5(2)
N(22)-C(22T)-H(22T)	125.2
N(23)-C(22T)-H(22T)	125.2
H(1W)-O(1W)-H(2W)	109(2)

Table 4. Anisotropic displacement parameters ($Å^2 \times 10^3$) for 4s. The anisotropic displacement factor exponent takes the form: $-2\pi^2[h^2a^{*2}U^{11} + ... + 2\ h\ k\ a^*\ b^*\ U^{12}]$.

	**U^{11}**	**U^{22}**	**U^{33}**	**U^{23}**	**U^{13}**	**U^{12}**
F(11)	25(1)	44(1)	14(1)	−3(1)	0(1)	2(1)
F(12)	23(1)	47(1)	16(1)	−1(1)	5(1)	2(1)
C(11)	19(1)	25(1)	16(1)	0(1)	−1(1)	0(1)
C(12)	26(1)	29(1)	13(1)	−2(1)	1(1)	−2(1)
C(13)	20(1)	29(1)	19(1)	2(1)	4(1)	1(1)
C(14)	19(1)	28(1)	20(1)	2(1)	−3(1)	−2(1)
C(15)	23(1)	29(1)	14(1)	2(1)	−3(1)	−2(1)
C(16)	21(1)	27(1)	18(1)	1(1)	5(1)	0(1)
C(17)	26(1)	32(1)	14(1)	4(1)	3(1)	−1(1)
N(15)	20(1)	45(1)	17(1)	4(1)	1(1)	1(1)
C(114)	23(1)	25(1)	17(1)	1(1)	−1(1)	0(1)
N(14)	16(1)	34(1)	18(1)	3(1)	−2(1)	0(1)
N(13)	18(1)	32(1)	16(1)	0(1)	−1(1)	−1(1)
C(11T)	19(1)	32(1)	18(1)	2(1)	4(1)	−1(1)
N(11)	20(1)	34(1)	21(1)	0(1)	4(1)	2(1)
N(12)	21(1)	33(1)	16(1)	0(1)	−1(1)	1(1)
C(12T)	22(1)	34(1)	14(1)	2(1)	−1(1)	0(1)
F(21)	27(1)	42(1)	11(1)	4(1)	0(1)	0(1)
F(22)	22(1)	52(1)	16(1)	−1(1)	7(1)	1(1)
C(21)	19(1)	24(1)	16(1)	0(1)	1(1)	−1(1)
C(22)	28(1)	26(1)	11(1)	2(1)	1(1)	−1(1)
C(23)	20(1)	28(1)	17(1)	−2(1)	3(1)	−1(1)
C(24)	20(1)	27(1)	20(1)	−4(1)	0(1)	1(1)
C(25)	23(1)	31(1)	16(1)	−2(1)	−1(1)	−1(1)
C(26)	22(1)	32(1)	18(1)	−2(1)	7(1)	−1(1)
C(27)	22(1)	33(1)	17(1)	−3(1)	4(1)	0(1)
N(25)	24(1)	45(1)	17(1)	−4(1)	3(1)	2(1)
C(214)	21(1)	30(1)	17(1)	3(1)	−1(1)	−2(1)
N(24)	16(1)	34(1)	20(1)	0(1)	1(1)	−1(1)
N(23)	17(1)	31(1)	17(1)	0(1)	−3(1)	−1(1)
C(21T)	20(1)	37(1)	23(1)	−4(1)	7(1)	−2(1)
N(21)	20(1)	38(1)	23(1)	−2(1)	4(1)	−2(1)
N(22)	23(1)	36(1)	20(1)	2(1)	2(1)	−1(1)
C(22T)	20(1)	34(1)	15(1)	2(1)	1(1)	0(1)
O(1W)	22(1)	41(1)	22(1)	0(1)	−3(1)	−1(1)

Table 5. Hydrogen coordinates (×10^4) and isotropic displacement parameters ($Å^2$ × 10^3) for 4s.

	x	y	z	U(eq)
H(13)	7205	5943	3255	27
H(15)	5997	6394	5706	27
H(114)	3215	6340	2650	26
H(11T)	298	5976	4259	28
H(12T)	1490	6578	1892	28
H(23)	11180	3847	759	26
H(25)	9952	3626	3229	28
H(214)	7178	3873	164	27
H(21T)	4267	4147	1760	31
H(22T)	5468	3459	-585	28
H(1W)	2480(15)	7859(16)	968(16)	43
H(2W)	3568(15)	7847(17)	1446(16)	43

Table 6. Torsion angles [°] for 4s.

C(16)-C(11)-C(12)-F(11)	179.3(2)
C(114)-C(11)-C(12)-F(11)	−1.9(3)
C(16)-C(11)-C(12)-C(13)	−0.3(3)
C(114)-C(11)-C(12)-C(13)	178.5(2)
F(11)-C(12)-C(13)-C(14)	179.9(2)
C(11)-C(12)-C(13)-C(14)	−0.5(4)
C(12)-C(13)-C(14)-C(15)	0.5(4)
C(12)-C(13)-C(14)-C(17)	178.8(2)
C(13)-C(14)-C(15)-C(16)	0.4(4)
C(17)-C(14)-C(15)-C(16)	−178.0(2)
C(14)-C(15)-C(16)-F(12)	178.6(2)
C(14)-C(15)-C(16)-C(11)	−1.3(4)
C(12)-C(11)-C(16)-F(12)	−178.7(2)
C(114)-C(11)-C(16)-F(12)	2.6(4)
C(12)-C(11)-C(16)-C(15)	1.2(4)
C(114)-C(11)-C(16)-C(15)	−177.5(2)
C(16)-C(11)-C(114)-N(14)	−13.8(4)
C(12)-C(11)-C(114)-N(14)	167.5(2)
C(11)-C(114)-N(14)-N(13)	179.7(2)
C(114)-N(14)-N(13)-C(11T)	176.7(2)
C(114)-N(14)-N(13)-C(12T)	−6.5(4)
C(12T)-N(13)-C(11T)-N(11)	0.2(3)
N(14)-N(13)-C(11T)-N(11)	177.7(2)
N(13)-C(11T)-N(11)-N(12)	−0.1(3)
C(11T)-N(11)-N(12)-C(12T)	0.0(3)
N(11)-N(12)-C(12T)-N(13)	0.1(3)
C(11T)-N(13)-C(12T)-N(12)	−0.2(3)
N(14)-N(13)-C(12T)-N(12)	−177.3(2)
C(26)-C(21)-C(22)-F(21)	−178.8(2)
C(214)-C(21)-C(22)-F(21)	2.4(3)

Table 6. *Cont.*

C(26)-C(21)-C(22)-C(23)	0.8(4)
C(214)-C(21)-C(22)-C(23)	−178.0(2)
F(21)-C(22)-C(23)-C(24)	180.0(2)
C(21)-C(22)-C(23)-C(24)	0.4(4)
C(22)-C(23)-C(24)-C(25)	−0.7(3)
C(22)-C(23)-C(24)-C(27)	−177.7(2)
C(23)-C(24)-C(25)-C(26)	−0.2(4)
C(27)-C(24)-C(25)-C(26)	176.8(2)
C(24)-C(25)-C(26)-F(22)	−177.8(2)
C(24)-C(25)-C(26)-C(21)	1.6(4)
C(22)-C(21)-C(26)-F(22)	177.5(2)
C(214)-C(21)-C(26)-F(22)	−3.8(4)
C(22)-C(21)-C(26)-C(25)	−1.8(4)
C(214)-C(21)-C(26)-C(25)	176.9(2)
C(22)-C(21)-C(214)-N(24)	−179.1(2)
C(26)-C(21)-C(214)-N(24)	2.2(4)
C(21)-C(214)-N(24)-N(23)	−177.9(2)
C(214)-N(24)-N(23)-C(22T)	14.7(4)
C(214)-N(24)-N(23)-C(21T)	−168.6(2)
C(22T)-N(23)-C(21T)-N(21)	−0.2(3)
N(24)-N(23)-C(21T)-N(21)	−177.7(2)
N(23)-C(21T)-N(21)-N(22)	0.1(3)
C(21T)-N(21)-N(22)-C(22T)	−0.1(3)
N(21)-N(22)-C(22T)-N(23)	0.0(3)
C(21T)-N(23)-C(22T)-N(22)	0.1(3)
N(24)-N(23)-C(22T)-N(22)	177.2(2)

Table 7. Hydrogen bonds for 4s [Å and °].

D-H...A	d(D-H)	d(H...A)	d(D...A)	<(DHA)
O(1W)-H(1W)...N(22)#1	0.855(10)	2.021(10)	2.870(3)	172(2)
O(1W)-H(2W)...N(12)#2	0.853(10)	2.008(11)	2.846(3)	167(2)
C(22T)-H(22T)...O(1W)#3	0.95	2.35	3.235(3)	155.2
C(12T)-H(12T)...O(1W)	0.95	2.37	3.254(3)	155.3
C(114)-H(114)...O(1W)	0.95	2.55	3.422(3)	153.5
C(11T)-H(11T)...N(15)#4	0.95	2.38	3.143(3)	136.5
C(21T)-H(21T)...N(25)#4	0.95	2.42	3.145(3)	132.6
C(25)-H(25)...N(15)#5	0.95	2.50	3.378(3)	153.0
C(15)-H(15)...N(25)#5	0.95	2.50	3.387(3)	155.4
C(23)-H(23)...N(21)#6	0.95	2.61	3.468(3)	151.1

Symmetry transformations used to generate equivalent atoms: #1 −x + 1/2, y + 1/2, −z; #2 x + 1/2, −y + 3/2,z; #3 −x + 1, −y + 1, −z; #4 x − 1, y, z; #5 −x + 2, −y + 1, −z + 1; #6 x + 1, y, z.

References

1. Cosier, J.; Glazer, A.M. A nitrogen-gas-stream cryostat for general X-ray diffraction studies. *J. Appl. Cryst.* **1986**, *19*, 105–107.
2. *CrysAlis CCD, CrysAlis RED, CrysAlisPRO*; Oxford Diffraction/Agilent Technologies UK Ltd: Yarnton, UK, 2009.
3. Sheldrick, G.M. A short history of *SHELX*. *Acta Crystallogr. Sect. A Found. Crystallogr.* **2008**, *64*, 112–122.
4. *XP—INTERACTIVE MOLECULAR GRAPHICS*, v. 5.1; Bruker Analytical X-ray System: Madison, WI, USA, 1998.
5. Spek, A.L. Single-crystal structure validation with the program *PLATON*. *J. Appl. Cryst.* **2003**, *36*, 7–13.
